# Supplementary material for: MicroRNA Expression Profile in Acute Ischemic Stroke
Source: Int J Mol Sci. 2025 Jan 17;26(2):747. doi: 10.3390/ijms26020747 (PMC11765720; doi:10.3390/ijms26020747)
Supplement: Supplementary file 1 [file ijms-26-00747-s001.zip › ijms-3382111-supplementary.pdf]

microRNA	DOI /PMID	Sample Type	Study Subject	# of subjects	Collection Time	Expression Level	Role	Diagnosis/Prognosis/Therapy
155	https://doi.org/10.1080/21655979.2021.1935066	serum, brain tissue	human, animal, in vitro	134 patients, 30 mice	patient serum collected at admission / collected from mouse model 8 days post-onset	increased	higher recurrence, apoptosis	Diagnosis/Prognosis/Therapy
	https://doi.org/10.1155/2020/6458204	plasma, brain tissue, SH-SY5Y cells	human, animal, in vitro	20 patients, 36 mice	collected 24 hours post-onset	increased	pro-apoptosis, pro-inflammatory	Therapy
	https://doi.org/10.1161/JAHA.118.009244	HBMECS	in vitro	N/A	N/A	*pathobiological study/intervention	decrease endothelial barrier function	Therapy
	https://doi.org/10.1523/JNEUROSCI.1641-15.2015	brain tissue	animal	190	collected 7 and 11 days post-onset	*pathobiological study/intervention	decrease blood brain barrier integrity, increase neuronal loss	Therapy
	https://doi.org/10.1111/jcmm.14358	brain tissue	animal	67	collected 0/12/24/48 hours post-onset	increased	neuroinflammation	Therapy
	https://doi.org/10.1590/0004-282X20190126	brain tissue, blood	animal	50	collected 48 hours post-reperfusion	increased (5 fold)	apoptosis	Prognosis
	https://doi.org/10.1186/s12974-016-0753-x	brain tissue	animal	N/A	collected 7 days post-onset	*pathobiological study/intervention	inflammation	Therapy
	DOI: 10.12659/MSM.898980	brain tissue, BV2 cells	animal, in vitro	N/A	N/A	increased	apoptosis	Therapy
	https://doi.org/10.2147/IJGM.S295939	serum	human	46 (50 control)	collected within 12 hours of admission	increased	inflammation	Diagnostic Biomarker
155-5p	10.26355/eurrev_202002_20198	brain tissue, SH-SY5Y cells	animal, in vitro	24	collected at 6/12/24 hours post-onset	increased	pro-apoptosis, pro-inflammatory, induces cell injury	Therapy
	https://doi.org/10.3390/ijms23010161	serum, brain tissue, spleen, liver	animal	N/A	collected 1/6/12/24/72 hours post-onset	increased in brain / decreased in serum	inflammation	Therapy
	https://doi.org/10.3389/fgene.2019.00814	brain tissue (Day 3 data)	animal	30	collected days 1/3/7/14/28 post-onset	increased (1.631498953 fold)	regulate synapses, cognition, axonogenesis, and ion transmembrane transport	Diagnostic Biomarker
		brain tissue (Day 7 data)	animal	30	collected days 1/3/7/14/28 post-onset	increased (3.513796089 fold)	regulate synapses, cognition, axonogenesis, and ion transmembrane transport	Diagnostic Biomarker
		brain tissue (Day 14 data)	animal	30	collected days 1/3/7/14/28 post-onset	increased (3.784658274 fold)	regulate synapses, cognition, axonogenesis, and ion transmembrane transport	Diagnostic Biomarker
		brain tissue (Day 28 data)	animal	30	collected days 1/3/7/14/28 post-onset	increased (3.679937664 fold)	regulate synapses, cognition, axonogenesis, and ion transmembrane transport	Diagnostic Biomarker
125a-5p	https://doi.org/10.5853%2Fjos.2020.05085	Plasma	Human	260	5 hrs post stroke	Upregulated	NA	Diagnostic biomarker
exosomal 155-5p	https://doi.org/10.1155/2022/8603427	brain tissue, choroid plexus epithelial cells	animal, in vitro	N/A	collected 3 days post-onset	increased	pro-inflammatory, pro-autophagy	Therapy
29	DOI: 10.26355/eurrev_202001_20068	brain tissue	animal	36	collected 24 hours post-onset	increased	anti-apoptosis	Therapy
125b-2	https://doi.org/10.3390%2Fijms15011418	Serum	Human	169	Within 24 hr post stroke	upregulated	NA	Diagnostic Biomarker
93	https://doi.org/10.1016/j.jocn.2018.12.003	Serum, neutrophils	Human	33	Within 6 hr post stroke	Increased	Anti-inflammatory	Therapy
29a	https://doi.org/10.3892/or.2019.6961	brain tissue, astrocytes	animal, in vitro	18	collected 24 or 48 hours post-onset	decreased	anti-apoptosis	Diagnostic Biomarkers/Therapy
	https://doi.org/10.3892/mmr.2018.9000	brain tissue	animal	6	N/A	*pathobiological study/intervention	neuroprotection	Diagnostic Biomarker / Therapy
	https://doi.org/10.3389/fgene.2021.642079	plasma, neutrophils, brain tissue, glial cells	human, animal	40 (27 control)	collected within 6 hours post-onset in patients / 24 hours post-onset in animals	decreased	decrease M1 microglial polarization and glutamate release	Diagnostic Biomarker / Therapy

				patients, 36 rats				
**30a**	https://doi.org/10.1186%2F1471-2377-13-178	Serum	Human	197	Within 24 hr post stroke	Decreased	NA	Diagnostic
**340-5p**	https://doi.org/10.1007/s12035-018-1295-2	Serum	Human	11	Within 24 hr post stroke	downregulated	fine-tuning ARG1 expression	Therapy
**29b**	https://doi.org/10.1038/jcbfm.2015.156	white blood cells, brain tissue	human, animal	58 (59 control), 54 mice	within 72 hours post-onset	decreased	reduce blood brain barrier disruption	Diagnostic Biomarker / Therapy
	https://doi.org/10.1038/jcbfm.2013.68	brain tissue	animal, in vitro	25	collected 48 hours post-onset	decreased	anti-apoptosis	Therapy
	https://doi.org/10.3892/mmr.2018.9000	brain tissue	animal	6	N/A	*pathobiological study/intervention	neuroprotection	Diagnostic Biomarker / Therapy
	DOI: 10.4103/1673-5374.314319	brain tissue, PC12 cells	animal, in vitro	48	N/A	increased	apoptosis, oxidative stress	Therapy
	https://doi.org/10.3892/etm.2017.5410	N2a neuroblastoma cells	in vitro	N/A	collected 0/6/3/12/24/48 hours post-onset	decreased	anti-apoptosis, anti-cytotoxicity	Therapy
	https://doi.org/10.3892/etm.2018.6622	N2a cells	in vitro	N/A	collected 48 hours post-onset	increased	apoptosis	Therapy
**29b-1**	https://doi.org/10.3390/ijms14012072	blood	human	8	collected 2-24 months post-onset	increased	pathogenesis, inflammation, thrombosis	Diagnostic Biomarker/Prognosis
**29b-2**	https://doi.org/10.1371/journal.pone.0083717	brain tissue, neuroblastoma cells	animal, in vitro	N/A	collected 8 hours post-OGD termination	increased	apoptosis	Therapy
**29c**	https://doi.org/10.1371/journal.pone.0058039	brain tissue, PC12 cells	animal, in vitro	N/A	collected at 1 or 3 days of reperfusion	decreased (2.4 fold)	decreases cell death	Therapy
	https://doi.org/10.3892/mmr.2018.9000	brain tissue	animal	6	N/A	*pathobiological study/intervention	neuroprotection	Diagnostic Biomarker / Therapy
**106a**	https://doi.org/10.3389/fmolb.2021.758742	serum	human	152 (71 control)	N/A	decreased	regulate inflammation and oxidative stress	Diagnostic Biomarker / Therapy
**106b**	https://doi.org/10.3390/ijms161024302	brain tissue	animal	N/A	collected 6 hours post-onset	increased (2 fold)	target genes involved in MAPK and PI3K-Akt signaling pathways	Diagnostic Biomarkers/Therapy
**106b-5p**	https://doi.org/10.1007/s12035-016-9842-1	brain tissue, pheochromocytoma cell line	animal, in vitro	72	collected at 26 hours post-onset	*pathobiological study/intervention	increased apoptosis, oxidative stress	Therapy
	https://doi.org/10.1016/j.jstrokecerebrovasdis.2014.06.002	plasma	human	136 (116 control)	collected 0-3/3-6/6-12/12-24 hours post-onset	increased (3.63-23.90 fold)	N/A	Diagnostic Biomarker
	https://doi.org/10.3389/fgene.2019.00814	brain tissue (Day 14 data)	animal	30	collected days 1/3/7/14/28 post-onset	increased (0.884192048 fold)	regulate synapses, cognition, axonogenesis, and ion transmembrane transport	Diagnostic Biomarker
**106b-5P**	10.1016/j.jstrokecerebrovasdis.2014.06.002	Plasma	human	136	0-3 hr	upregulated	NA	Diagnostic Biomarker
**4306**	10.1016/j.jstrokecerebrovasdis.2014.06.002	Plasma	human	136	0-3 hr	upregulated	NA	Diagnostic Biomarker
**exosomal 106b-5p**	https://doi.org/10.1186/s13287-021-02668-0	brain tissue, ADSCs, microglia	animal, in vitro	1-28	collected 7 and 14 days post-onset	*pathobiological study/intervention	promoted M2 polarization of microglia, anti-inflammatory	Therapy
**130a**	https://doi.org/10.1096/fj.201700139RRR	brain tissue, atrocytes, pericytes	animal, in vitro	6-8	collected at 2/4/6 hours, 1/3/7 days post-onset	increased	blood brain barrier dysfunction	Therapy
	https://doi.org/10.1016/j.biopha.2019.109117	brain tissue, PC12 cells	animal, in vitro	N/A	collected at 24 hours post-onset	decreased	anti-apoptosis, increased cell survival	Therapy
	https://doi.org/10.1111/jcmm.15732	brain tissue	animal, in vitro	120	N/A	increased	apoptosis, decrease angiogenesis/viability	Therapy
**130a-3p**	https://doi.org/10.3389/fnins.2021.601850	whole blood, Neuro2A cells	human, animal, in vitro	N/A	collected 24 hours post-reperfusion	decreased	anti-neurite outgrowth	Diagnostic Biomarker
	https://doi.org/10.3389/fgene.2019.00814	brain tissue (Day 7 data)	animal	30	collected days 1/3/7/14/28 post-onset	increased (0.914071974 fold)	regulate synapses, cognition, axonogenesis, and ion transmembrane transport	Diagnostic Biomarker
**200a**	https://doi.org/10.1371/journal.pone.0172178	neural stem cells	in vitro	N/A	N/A	increased	apoptosis	Therapy

**200c**	https://doi.org/10.1161/STROKEAHA.114.007041	brain tissue	animal, in vitro	124	collected at 1/3/24/>24 hours post-onset	*pathobiological study/intervention	oxidative injury, neuronal death	Prognosis
**200b-3p**	https://doi.org/10.1002/brb3.2518	serum	human	189 (106 control)	collected within 24 hours of onset	increased	pathogenesis	Diagnostic Biomarker/Prognosis
	https://doi.org/10.1016/j.neuint.2021.105146	brain tissue	animal	31	collected at 30 min or 2 hours post-reperfusion	increased	neuronal cell death	Therapy
	https://doi.org/10.3389/fgene.2019.00814	brain tissue (Day 3 data)	animal	30	collected days 1/3/7/14/28 post-onset	increased (1.90299797 fold)	regulate synapses, cognition, axonogenesis, and ion transmembrane transport	Diagnostic Biomarker
		brain tissue (Day 28 data)	animal	30	collected days 1/3/7/14/28 post-onset	increased (0.917967505 fold)	regulate synapses, cognition, axonogenesis, and ion transmembrane transport	Diagnostic Biomarker
**exosomal 200b-3p**	https://doi.org/10.1186/s13287-021-02668-0	brain tissue, ADSCs, microglia	animal, in vitro	28	collected 7 and 14 days post-onset	*pathobiological study/intervention	promoted M2 polarization of microglia, anti-inflammatory	Therapy
**210**	https://doi.org/10.1111/cns.12589	whole blood, brain tissue	human, animal	5 (5 control) patients, 124 mice	collected within 48 hours-10 days post-onset	increased (>1.5 fold)	promote angiogenesis/neurogenesis	Prognosis / Therapy
	https://doi.org/10.1016/j.expneurol.2017.10.024	brain tissue	animal	96	collected 24 hours post-onset	increased	proinflammatory	Therapy
	https://doi.org/10.1002/jcla.24073	serum	human	52 (52 control)	collected at admission, 24/48 hours post-onset, at discharge, & 3 months later	decreased	neuroprotection	Diagnostic Biomarker/Prognosis
	https://doi.org/10.1161/JAHA.116.005052	brain tissue, EPCs, HEK293T cells	animal, in vitro	18	collected 1/3/7 days post-onset	increased	neovascularization, NPC accumulation, anti-apoptosis, cell proliferation	Therapy
	https://doi.org/10.1155/2021/4464945	serum	human	76 (64 control)	collected within 6 hours post-onset	decreased	neuroprotection	Diagnostic Biomarker/Prognosis
	10.26355/eurrev_201903_17403	brain tissue	animal, in vitro	40	collected 24 hours post-onset	increased	apoptosis	Therapy
	https://doi.org/10.3390/ijms18071356	brain tissue	animal	N/A	collected 48 hours post-onset	*pathobiological study/intervention	negatively regulates blood brain barrier integrity	Therapy
	https://doi.org/10.1002/brb3.835	serum	human	167 (82 control)	collected within 1-48 hours post-delivery	decreased	anti-apoptosis, angiogenesis, neuroprotection	Diagnostic Biomarker/Prognosis
	https://doi.org/10.1186/s12974-020-02068-w	brain tissue, microglial cells	animal, in vitro	N/A	collected 3/12/24 hours post-onset	increased	inflammation	Diagnostic Biomarker / Therapy
	https://doi.org/10.1038/s41423-019-0257-6	brain tissue, microglial cells, mononuclear cells	animal, in vitro	N/A	collected 6 and 24 hours post-onset	increased	inflammation	Therapy
	PMID: 27906445	peripheral blood	human	150 (50 control)	collected before hospital admission and patient treatment	decreased	anti-apoptosis, angiogenesis, cell proliferation	Diagnostic Biomarker
**210-3p**	https://doi.org/10.1111/jnc.15347	brain tissue	animal, in vitro	109	collected 1/3/24 hours post-onset	*pathobiological study/intervention	apoptosis, cell proliferation	Diagnostic Biomarker / Prognosis/ Therapy
**exosomal 210**	https://doi.org/10.1186/s12951-019-0461-7	mesenchymal stem cells, brain tissue	animal, in vitro	N/A	collected 12 hours after administration	*pathobiological study/intervention	promote angiogensis	Therapy
	https://doi.org/10.1016/j.expneurol.2022.114211	endothelial progenitor cells, SH-SY5Y neuronal cells	in vitro	N/A	collected 30 hours post-onset	*pathobiological study/intervention	anti-apoptosis, increase cell viability	Therapy
**365**	https://doi.org/10.1002/glia.23308	cortical astrocytes, brain tissue	animal	N/A	collected at 1/6/12/24 hours post-onset	increased	inhibit astrocyte to neuron conversion	Therapy
	https://doi.org/10.1007/s12264-019-00371-y	brain tissue	animal, in vitro	N/A	collected at 24 and 72 hours post-onset	increased	increase oxidative damage and neuronal cell death	Therapy
**3473**	https://doi.org/10.3390/ijms23010161	serum, brain tissue, spleen, liver	animal	N/A	collected 1/6/12/24/72 hours post-onset	increased in brain / decreased in serum	inflammation	Therapy

**3473b**	https://doi.org/10.1038/s41419-017-0014-7	brain tissue, microglial cells	animal, in vitro	N/A	collected post 1 hour MCAO & 6 hour reperfusion	increased	promotes neuroinflammation	Therapy
**9**	https://doi.org/10.1007/s12035-015-9605-4	brain tissue	animal, in vitro	60	collected at 0/12/24 hours post-onset	decreased	antineuronal apoptosis	Therapy
	https://doi.org/10.1016/j.jocn.2014.05.042	serum	human	31 (11 control)	collected within 24 hours post-onset	decreased	anti-neuroinflammation	Therapy
	https://doi.org/10.1371/journal.pone.0228825	brain tissue, PC12 cells	animal, in vitro	168	collected 15 days post-onset	increased	inflammation, cell death	Therapy
	https://pubmed.ncbi.nlm.nih.gov/31949675	serum, neuronal cells	human, in vitro	65 (55 control)	collected within 24 hours post-onset	increased	inflammation, apoptosis	Diagnostic Biomarker/Prognosis
	https://doi.org/10.3389/fmolb.2021.758742	serum	human	152 (71 control)	N/A	increased	inflammation, neural damage, apoptosis	Diagnostic Biomarker / Therapy
**exosomal 9**	https://doi.org/10.1371/journal.pone.0163645	serum	human	131	collected at mean 16.5 hours post-onset	increased (16 fold)	inflammation, infarct size	Diagnostic Biomarker/Prognosis
**21**	https://doi.org/10.18632/aging.203530	brain tissue	animal, in vitro	N/A	collected 24 hours post-onset	decreased	anti-apoptosis	Therapy
	https://doi.org/10.1007/s12031-018-1067-5	brain tissue	animal	96	collected 24 hours post-reperfusion	decreased	decrease infarct size, cerebral edema, blood brain barrier permeability	Therapy
	PMID: 27508039	brain tissue	animal, in vitro	N/A	collected 25 hours post-onset	*pathobiological study/intervention	apoptosis	Therapy
	https://doi.org/10.3389/fmolb.2022.914506	serum	human	60 (60 control)	collected with 24 hours of symptom onset	increased (8.2 fold)	apoptosis, inflammation	Diagnostic Biomarker
**424**	https://doi.org/10.1161/STROKEAHA.111.000504	plasma, brain tissue, microglial cells	human, animal, in vitro	11 patients	collected at 4/8/24 hours post-onset	decreased	reduce neuronal apoptosis and microglia activation	Therapy
**424**	https://doi.org/10.14336%2FAD.2017.0602	Serum	Human	40	Within 6 hr post stroke	Increased in neutrophils and lymphocytes Not changed in plasma	Anti-inflammatory	Therapy
	https://doi.org/10.1111/febs.15029	brain tissue, human U87 and U251 cells, astrocytes	animal, in vitro	N/A	collected 1/3/14 days post-reperfusion	*pathobiological study/intervention	suppress reactive astrocytosis, preserve neurons/axons	Prognosis/Therapy
	DOI: 10.26355/eurrev_201803_14490	PC12 cells	in vitro	N/A	N/A	increased	anti-apoptosis, increase cell viability	Therapy
	https://doi.org/10.3892/etm.2021.10888	PC12 cells	in vitro	N/A	N/A	decreased	anti-apoptosis, increase cell viability	Therapy
	https://doi.org/10.14336/AD.2017.0602	lymphocytes, neutrophils and plasma	human	40 (27 control)	collected within 6 hours of symptoms	increased	immunosuppressive	Diagnostic Biomarker
	https://doi.org/10.2147/IJGM.S340586	serum	human	142 (50 control)	collected within 24 hours post-onset	increased	severe neurological impairment	Diagnostic Biomarkers/Prognosis
**exosomal 424**	https://doi.org/10.3390/biomedicines9070786	serum	human	81 (22 control)	collected within 24 hours post-onset	decreased	differentiate stroke subgroups, angiogenesis	Diagnostic Biomarker / Prognosis/ Therapy
**592**	https://doi.org/10.1523/JNEUROSCI.1982-13.2014	brain tissue	animal, in vitro	N/A	collected at 4/8 hours post-onset	decreased	antiapoptotic cell death	Therapy
**191**	https://doi.org/10.18632/aging.101948	plasma	human, animal , in vitro	18 (18 control) patients, 12 rats	collected at 6/24/48 hours post-onset	increased	apoptosis / inhibit angiogenesis	Diagnostic Biomarker / Therapy
**191-5p**	DOI: 10.4103/0028-3886.333459	brain tissue	animal	53	collected at 24 and 48 hours post-onset	increased	disturb angiogenesis	Therapy
	https://doi.org/10.3389/fnins.2021.738576	plasma	animal	3	collected 3 hours post-onset	increased (0.98 fold)	regulated proteolysis and chemokine signaling	Diagnostic Biomarker

**30a**	Down-Regulation of miRNA-30a Alleviates Cerebral Ischemic Injury Through Enhancing Beclin 1-Mediated Autophagy | SpringerLink	brain tissue	animal, in vitro	N/A	collected at 6/12/24/48 hours post-onset	decreased	ischemia-induced neuronal death	Therapy
	https://doi.org/10.1186/1471-2377-13-178	plasma	human	197 (50 control)	collected at 24 hours, 1/4/24/48 weeks post-onset	decreased	N/A	Diagnostic Biomarker
**146a**	https://doi.org/10.1016/j.brainres.2016.07.034	SK-N-SH cells	in vitro	N/A	N/A	decreased	oxygen-glucose deprivation/ reperfusion induced apoptosis	Therapy
	https://doi.org/10.2147/IJGM.S213535	serum	human	44 (22 control)	collected within 24 hours post-onset of symptoms	decreased (1.98 fold)	neuroprotection, anti-apoptosis, proliferation	Diagnostic Biomarker
**146b**	https://doi.org/10.1159/000486916	serum	human	128 (102 control)	collected < or = 24 hours post-onset	increased	pro-inflammatory	Diagnostic Biomarkers/Prognosis
**146b-5p**	https://doi.org/10.3349/ymj.2020.61.8.660	brain tissue, PC12 cells	animal, in vitro	N/A	collected 72 hours post-onset	decreased	anti-inflammatory, antioxidant	Therapy
	https://doi.org/10.3389/fgene.2019.00814	brain tissue (Day 1 data)	animal	30	collected days 1/3/7/14/28 post-onset	increased (0.540268267 fold)	regulate synapses, cognition, axonogenesis, and ion transmembrane transport	Diagnostic Biomarker
		brain tissue (Day 14 data)	animal	30	collected days 1/3/7/14/28 post-onset	increased (0.814333705 fold)	regulate synapses, cognition, axonogenesis, and ion transmembrane transport	Diagnostic Biomarker
**146a/b**	https://doi.org/10.1016/j.kjms.2017.05.010	brain tissue, EPCs	animal, in vitro	80	collected 6/12/24 hours post-onset	decreased	proliferation, migration, angiogenesis	Therapy
**1224**	https://doi.org/10.1186/s12974-021-02181-4	splenic natural killer cells	animal	N/A	collected 3 days post-onset	increased (>2 fold)	inhibit cell proliferation, negative regulator of NK cell activation	Prognosis
	https://doi.org/10.3389/fnmol.2014.00011	brain tissue	animal	N/A	collected at 8 hours post-onset	increased	CD73/PKN2 targets/mechanisms	Prognosis/Therapy
	https://doi.org/10.1371/journal.pone.0163645	serum	human	131	collected at mean 16.5 hours post-onset	increased (4 fold)	increase inflammation, infarct size	Diagnostic Biomarker/Prognosis
**exosomal 124**	https://doi.org/10.1016/j.omtn.2017.04.010	bone marrow mesenchymal stem cells, brain tissue	animal, in vitro	N/A	collected 24 hours post-onset	*pathobiological study/intervention	promote neurogenesis	Therapy
	https://doi.org/10.7150/thno.48761	microglia, brain tissue, astrocytes	animal, in vitro	80	collected 7 or 14 days post-onset	*pathobiological study/intervention	reduce glial scar formation, astrocyte proliferation/migration	Prognosis/Therapy
	doi:10.7150/thno.30879	BV2 cells, brain tissue	animal, in vitro	48	collected 3 days post-onset	*pathobiological study/intervention	promote neuron survival, anti-inflammatory	Therapy
**124**	https://doi.org/10.1177/10760296211035446	serum	human	108 (108 control)	collected 24/48/72 hours post-onset	decreased	anti-inflammatory	Diagnostic Biomarker / Prognosis/ Therapy
	https://doi.org/10.1016/j.jocn.2014.05.042	serum	human	31 (11 control)	collected within 24 hours post-onset	decreased	anti-neuroinflammation	Therapy
	https://doi.org/10.1111/cns.12142	brain tissue	animal, in vitro	N/A	collected at 24 hours post-onset	increased	anti-apoptosis	Therapy
	https://doi.org/10.1007/s11481-016-9700-y	brain tissue	animal	34	collected at 6 and 14 days post-onset	*pathobiological study/intervention	anti-inflammatory	Prognosis/Therapy
	DOI: 10.26355/eurrev_201908_18556	brain tissue	animal	60	collected 24 hours post-onset	decreased	anti-apoptosis	Therapy
	https://doi.org/10.1371/journal.pone.0193609	brain tissue, SVZ cells	animal, in vitro	73	collected 2 and 14 days post-onset	*pathobiological study/intervention	anti-inflammatory	Therapy
	https://doi.org/10.1007/s13205-019-1914-2	serum, neuroblastoma cells	human, in vitro	40 (40 control)	N/A	increased	decrease proliferation, cell differentiation	Diagnostic Biomarker
	https://doi.org/10.3389/fnins.2021.649982	brain tissue	animal, in vitro	N/A	collected 0.5/2/4/6/12/24/48/72 hours post-onset	decreased	decrease neuronal death	Therapy
	https://doi.org/10.3892/etm.2017.4424	PC12 cells	in vitro	N/A	collected 0/3/6/12/24 hours post-onset	*pathobiological study/intervention	anti-apoptosis	Therapy

**15a/16-1 cluster**	https://doi.org/10.1161/circresaha.119.315886	cerebral microvessels, endothelial cells	animal, in vitro	N/A	collected 28 days post-onset	increased	negative regulator of angiogenesis	Therapy
	https://doi.org/10.1161/STROKEAHA.117.017284	brain tissue	animal	N/A	collected 1-7 days post-onset	*pathobiological study/intervention	pro-inflammatory	Therapy
**223**	https://doi.org/10.5114/fn.2018.74659	brain tissue	in vitro	N/A	N/A	increased	promote cell death, inhibit cell proliferation	Prognosis
	https://doi.org/10.1186/1471-2377-14-77	blood, brain tissue	human, animal	79 (75 control) patients, 12 mice	collected within 72 hours post-onset in patients / collected 24 hours post-onset in mice	increased	inflammation	Diagnostic Biomarker/Therapy
**exosomal 223**	https://doi.org/10.3389/fneur.2017.00057	whole blood	human	50 (33 control)	collected within 72 hours post-onset	increased	poor prognosis	Diagnostic Biomarker / Prognosis/ Therapy
**195**	https://doi.org/10.1186/s10020-020-00150-w	brain tissue	animal, in vitro	24	N/A	increased	enhance synaptic plasticity, reduce apoptosis	Therapy
	https://doi.org/10.3892/mmr.2017.7230	brain tissue, HUVECs	animal, in vitro	N/A	collected at 0/1/2/6/12/24 hours post-onset in vitro	decreased	angiogenesis	Therapy
	https://doi.org/10.1016/j.omtm.2018.11.011	brain tissue, SH-SY5Y neural cells, HUVECs	animal, in vitro	N/A	collected 24 hours and 3/5 days post-onset	decreased	anti-apoptosis, neural regeneration, anti-inflammatory	Therapy
**exosomal 195**	https://doi.org/10.1161/STROKEAHA.120.031728	brain tissue, endothelial cells	animal , in vitro	8	N/A	increased	promote axonal growth, homeostasis, and plasticity	Therapy
**195-5p**	https://doi.org/10.3390/jcm8020130	serum	human	36 (20 control)	collected at 0/24/48 hours after admission	increased	anri-angiogenesis	Diagnostic Biomarker / Prognosis/ Therapy
	https://doi.org/10.3390/ijms21207615	serum	human	78 (20 control)	collected at admission, 24/72 hours post-onset	increased	anti-angiogenesis, neuronal damage	Diagnostic Biomarker/Prognosis
**145**	https://doi.org/10.1016/j.brainresbull.2018.08.023	brain tissue, neural stem cells	animal, in vitro	100	N/A	increased	promote cell proliferation, inhibit cell apoptosis	Therapy
	PMID: 26722607	brain tissue, endothelial progenitor cells	animal, in vitro	N/A	N/A	increased	cell proliferation and migration, recanalization of arterial thrombosis	Therapy
	https://doi.org/10.4238/2012.january.27.1	peripheral whole blood	human	32 (14 control)	N/A	increased	stages of vascular reendothelialization	Diagnostic Biomarkers/Therapy
	https://doi.org/10.1155/2017/9530951	astrocytes	in vitro	N/A	N/A	decreased	anti-apoptosis	Therapy
	https://doi.org/10.1042/BSR20201154	blood	human	24 (24 control)	N/A	increased	cell growth, differentiation, apoptosis, angiogenesis	Diagnostic Biomarker/Therapy
	https://doi.org/10.1002/cbf.3116	serum	human	146 (96 control)	collected within 24 hours post-onset	increased	inflammation	Diagnostic Biomarker/Therapy
	https://doi.org/10.1016/j.jstrokecerebrovasdis.2016.03.023	peripheral blood	human, in vitro	5 (5 control)	N/A	increased	apoptosis	Diagnostic Biomarker / Prognosis/ Therapy
**339**	https://doi.org/10.3389/fneur.2020.00436	PC12 cells	in vitro	N/A	N/A	increased	inhibit cell proliferation, induce apoptosis	Therapy
	https://doi.org/10.1042/BSR20201154	blood	human	24 (24 control)	N/A	increased	cell growth, differentiation, apoptosis, angiogenesis	Diagnostic Biomarker/Therapy
**exosomal 339**	https://doi.org/10.3390/biomedicines9070786	serum	human	81 (22 control)	collected within 24 hours post-onset	decreased	differentiate stroke subgroups	Diagnostic Biomarker / Prognosis/ Therapy
**410**	https://doi.org/10.1016/j.brainresbull.2018.09.009	serum, brain tissue	animal	45	collected 48 hours post-onset	decreased	cell survival, inhibit apoptosis, infarct size	Therapy
	https://doi.org/10.1002/brb3.2293	serum, PC12 and SH-SY5Y cells	human, in vitro	102 (60 control)	collected within 6 hours after birth	decreased	anti-apoptosis, anti-inflammatory, promote cell viability	Diagnostic Biomarker
**128**	https://doi.org/10.1002/acn3.51379	peripheral blood, brain tissue, astrocytes	human, animal, in vitro	15 (15 control) patients, 72 rats	N/A	*pathobiological study/intervention	pro-apoptosis	Therapy

	https://doi.org/10.1177/0963689719846848	plasma, lymphocytes, neutrophils, brain tissue	human, animal, in vitro	40 (25 control) patients	collected within 72 hours post-onset	increased	inflammation, increase neuronal injury	Diagnostic Biomarker / Prognosis/ Therapy
	https://doi.org/10.1007/s12035-020-02018-w	whole blood, plasma, SH-SY5Y cells	human, animal, in vitro	48 patients, 10 (10 control) piglets	collected within 3 hours post-delivery in patients / collected at 0/1/2/8/72 hours post-onset in animals	increased (resolves after 1 hour)	neuronal injury	Diagnostic Biomarker
**128-3p**	https://doi.org/10.1007/s12031-016-0871-z	brain tissue, embryonic kidney cells,SH-SY5Y cells	animal, in vitro	N/A	collected at 0/20/40/60 mins /2/4/ and 8 hours post-onset	increased	protection against ischemia-induced neuronal cell death	Therapy
	https://doi.org/10.6061/clinics/2021/e2958	serum	human	88 (88 control)	collected 12-14 hours after diagnosis (diagnosis within 6 hours post-onset)	increased	pro-apoptosis	Diagnostic Biomarker/Prognosis
	https://doi.org/10.1002/iub.2357	brain tissue	animal, in vitro	24	collected at 9/12/18 hours post-onset	increased	pro-apoptosis	Therapy
	https://doi.org/10.1186/s40364-017-0104-9	CSF	human	21 (21 control)	collected 3 days post-onset	increased	apoptosis, inflammation	Diagnostic Biomarker
	https://doi.org/10.3389/fgene.2019.00814	brain tissue (Day 1 data)	animal	30	collected days 1/3/7/14/28 post-onset	increased (0.813698845 fold)	regulate synapses, cognition, axonogenesis, and ion transmembrane transport	Diagnostic Biomarker
**exosomal 128-3p**	https://doi.org/10.1186/s13287-021-02668-0	brain tissue, ADSCs, microglia	animal, in vitro	28	collected 7 and 14 days post-onset	*pathobiological study/intervention	promoted M2 polarization of microglia, anti-inflammatory	Therapy
	https://doi.org/10.3389/fnmol.2022.874903	serum, brain tissue	human, animal	40 (33 control) patients	collected within 18.5 hours post-admission in patients / collected 1 day post-onset in animals	increased	blood brain barrier disruption	Diagnostic Biomarker/Prognosis
**122**	10.2147/NDT.S271320	serum, brain tissue, neuroblastoma cells	human, animal, in vitro	81 (81 control) patients, 24 mice	N/A	decreased	decrease apoptosis, autophagy	Therapy
	https://doi.org/10.1371/journal.pone.0099283	peripheral blood	human	24 (24 control)	collected at 72 hours post-onset	decreased (2.29 fold)	inflammation, immune regulation, cell proliferation	Diagnostic Biomarker/Prognosis
	DOI: 10.12659/MSM.915825	brain tissue	animal, in vitro	N/A	collected at 6/12/24 hours post-onset	increased	apoptosis	Therapy
	https://doi.org/10.1042/BSR20201154	blood	human	24 (24 control)	N/A	decreased	cell growth, differentiation, apoptosis, angiogenesis	Diagnostic Biomarker/Therapy
	https://doi.org/10.1177/0271678X15610786	whole blood	animal	54	collected 24 hours post-onset	decreased	anti-inflammatory, maintain vessel integrity	Therapy
	https://doi.org/10.1016/j.jstrokecerebrovasdis.2016.03.023	peripheral blood	human, in vitro	5 (5 control) patients	N/A	decreased	neuroprotection, immune activation	Diagnostic Biomarker / Prognosis/ Therapy
	https://doi.org/10.3389/fnins.2018.00767	brain tissue	animal	24	collected 1 day post-onset	*pathobiological study/intervention	anti-inflammatory	Therapy
	https://doi.org/10.3892/etm.2021.10048	brain tissue	animal, in vitro	60	collected 24 hours post-onset	increased	neuroprotection, anti-oxidative	Therapy
**298**	https://doi.org/10.1159/000491810	brain tissue, N2a cells	animal, in vitro	N/A	collected 24 hours post-reperfusion	decreased	enhance cell apoptosis, autophagy	Therapy
**exosomal 298**	https://doi.org/10.1161/STROKEAHA.120.031728	brain tissue, endothelial cells	animal , in vitro	8	N/A	increased	promote axonal growth, homeostasis, and plasticity	Therapy
**199a**	DOI: 10.26355/eurrev_202006_21532	brain tissue	animal	36	collected 72 hours post-onset	increased	pro-inflammatory, autophagy	Prognosis/Therapy
**137**	https://doi.org/10.18632/aging.103301	brain tissue, astrocytes	animal	190	collected 7 days post-onset	decreased	anti-inflammatory, antiapoptotic	Therapy

	https://doi.org/10.1155/2021/4464945	serum	human	76 (64 control)	collected within 6 hours post-onset	decreased	neuroprotection	Diagnostic Biomarker/Prognosis
**exosomal 137**	https://doi.org/10.18632%2Faging.202373	BV2 cells, brain tissue	animal , in vitro	36	collected 3 days post-onset	*pathobiological study/intervention	anti-apoptosis	Therapy
**221-3p**	https://doi.org/10.1016/j.jstrokecerebrovasdis.2016.12.019	Serum	Human	78	Within 20 hr post stroke	Downregulated	NA	Diagnostic biomarker
**382-5p**	https://doi.org/10.1016/j.jstrokecerebrovasdis.2016.12.019	Serum	Human	78	Within 20 hr post stroke	Downregulated	NA	Diagnostic biomarker
**22**	https://doi.org/10.3892/mmr.2019.10269	brain tissue, peripheral blood, PC12 cells	animal , in vitro	16	N/A	decreased	anti-inflammatory	Therapy
	https://doi.org/10.3389/fnins.2021.738576	plasma	animal	3	collected 3 hours post-onset	decreased (2.07 fold)	regulated proteolysis and chemokine signaling	Diagnostic Biomarker
**let 7b**	https://doi.org/10.1186/1471-2377-13-178	plasma	human	197 (50 control)	collected at 24 hours, 1/4/24/48 weeks post-onset	increased (normal at 48 weeks)	pro-inflammatory	Diagnostic Biomarker
**let 7b-3p**	10.21307/ane-2019-018	blood, brain tissue	animal	24	collected at 4 hours post-onset	decreased (>1.5 fold)	diagnostic of hyperacute stage	Diagnostic Biomarker
**exosomal let 7b-5p**	https://doi.org/10.7150%2Fthno.56367	brain tissue, ReN cells, BV2 cells, microglia	animal , in vitro	N/A	collected 24 hours post-reperfusion	increased	anti-inflammatory	Therapy
**let-7g**	https://doi.org/10.3389/fcell.2020.00632	brain tissue	animal	N/A	collected 72 hours post-onset	decreased	anti-inflammatory	Prognosis
	https://doi.org/10.1016/j.bbi.2020.01.026	brain tissue, microvessels, BMVECs	animal , in vitro	N/A	collected 96 hours post-onset in vivo/ 24/48/72/96 hours in vitro	decreased	anti-inflammatory, preserves blood brain barrier, anti-neuronal death	Therapy
**exosomal let-7g**	doi:10.7150/thno.56367	BV2 microglial cells, ReN cells, brain tissue	animal , in vitro	16	collected at 24 hours post-onset	*pathobiological study/intervention	anti-inflammatory	Therapy
**let-7g-5p**	https://doi.org/10.3389/fnins.2021.738576	plasma	animal	3	collected 3 hours post-onset	increased (0.80 fold)	regulated proteolysis and chemokine signaling	Diagnostic Biomarker
**exosomal let-7g-5p**	https://doi.org/10.7150%2Fthno.56367	brain tissue, ReN cells, BV2 cells, microglia	animal , in vitro	N/A	collected 24 hours post-reperfusion	increased	anti-inflammatory	Therapy
**222**	10.1007/s10072-018-3499-7	Serum	human	148	Within 24 hr post stroke	upregulated	NA	Prognostic/Therapy
**218**	10.1007/s10072-018-3499-7	Serum	human	148	Within 24 hr post stroke	upregulated	NA	Prognostic/Therapy
**185**	10.1007/s10072-018-3499-7	Serum	human	148	Within 24 hr post stroke	upregulated	NA	Prognostic/Therapy
**126**	10.1007/s10072-018-3499-7	Serum	human	148	Within 24 hr post stroke	Downregulated	NA	Prognostic/Therapy
**130a**	10.1007/s10072-018-3499-7	Serum	human	148	Within 24 hr post stroke	Downregulated	NA	Prognostic/Therapy
**exosomal let-7i**	doi:10.7150/thno.56367	BV2 microglial cells, ReN cells, brain tissue	animal , in vitro	16	collected at 24 hours post-onset	*pathobiological study/intervention	anti-inflammatory	Therapy
**let-7i**	https://doi.org/10.1371/journal.pone.0099283	peripheral blood	human	24 (24 control)	collected at 72 hours post-onset	decreased (2.07 fold)	inflammation, immune regulation, cell proliferation	Diagnostic Biomarker/Prognosis
	https://doi.org/10.1073/pnas.1803384115	brain tissue, astrocytes	animal , in vitro	N/A	collected at 24 hours post-onset	increased	anti-synaptogenesis, decrease cell viability	Therapy
**Let-7i**	https://doi.org/10.1212%2FWNL.0000000000003354	Leukocytes	Human	106	Within 24 hr post stroke	downregulated	targets HMGB1, CD86, and CXCL8 which promote inflammation and tissue injury	Therapy
**exosomal let 7i-5p**	https://doi.org/10.7150%2Fthno.56367	brain tissue, ReN cells, BV2 cells, microglia	animal , in vitro	N/A	collected 24 hours post-reperfusion	increased	anti-inflammatory	Therapy
**exosomal 21-5p**	https://doi.org/10.7150%2Fthno.56367	brain tissue, ReN cells, BV2 cells, microglia	animal , in vitro	N/A	collected 24 hours post-reperfusion	increased	anti-inflammatory	Therapy

**exosomal 98-5p**	https://doi.org/10.3390/biom12070883	brain tissue, bone mesenchymal stem cells, HUVECs	animal , in vitro	N/A	collected 3 and 14 days post-onset	increased	angiogenesis	Therapy
	https://doi.org/10.7150%2Fthno.56367	brain tissue, ReN cells, BV2 cells, microglia	animal , in vitro	N/A	collected 24 hours post-reperfusion	increased	anti-inflammatory	Therapy
	https://doi.org/10.3389/fnmol.2022.874903	serum, brain tissue	human, animal	40 (33 control) patients	collected within 18.5 hours post-admission in patients / collected 1 day post-onset in animals	increased	blood brain barrier disruption	Diagnostic Biomarker/Prognosis
**exosomal 99a**	https://doi.org/10.7150%2Fthno.56367	BV2 microglial cells, ReN cells, brain tissue	animal , in vitro	16	collected at 24 hours post-onset	*pathobiological study/intervention	anti-inflammatory	Therapy
**99a-5p**	DOI: 10.4103/bc.bc_1_17	plasma, neutrophils, lymphocytes	human	21 (8 control)	collected within 6 hours post-onset	increased (neutrophils)	regulate immune cell proliferation and coagulation/fibrinolysis	Diagnostic Biomarker
	https://doi.org/10.3389/fgene.2019.00814	brain tissue (Day 1 data)	animal	30	collected days 1/3/7/14/28 post-onset	increased (0.476054207 fold)	regulate synapses, cognition, axonogenesis, and ion transmembrane transport	Diagnostic Biomarker
		brain tissue (Day 7 data)	animal	30	collected days 1/3/7/14/28 post-onset	increased (0.88169521 fold)	regulate synapses, cognition, axonogenesis, and ion transmembrane transport	Diagnostic Biomarker
**exosomal 99a-5p**	https://doi.org/10.7150%2Fthno.56367	brain tissue, ReN cells, BV2 cells, microglia	animal , in vitro	N/A	collected 24 hours post-reperfusion	increased	anti-inflammatory	Therapy
**7i-5p**	10.1016/j.thromres.2017.09.00	Serum	Human	86	Within 24 hr post stroke	Increased	NA	Therapy
**371b-5p**	10.1016/j.thromres.2017.09.00	Serum	Human	86	Within 24 hr post stroke	Increased	NA	Therapy
**4693-3p**	10.1016/j.thromres.2017.09.00	Serum	Human	86	Within 24 hr post stroke	Decreased	NA	Therapy
**4666a-5p**	10.1016/j.thromres.2017.09.00	Serum	Human	86	Within 24 hr post stroke	Decreased	NA	Therapy
**3154**	10.1016/j.thromres.2017.09.00	Serum	Human	86	Within 24 hr post stroke	Decreased	NA	Therapy
**4768-3p**	10.1016/j.thromres.2017.09.00	Serum	Human	86	Within 24 hr post stroke	Decreased	NA	Therapy
**550a-5p**	10.1016/j.thromres.2017.09.00	Serum	Human	86	Within 24 hr post stroke	Decreased	NA	Therapy
**3685**	10.1016/j.thromres.2017.09.00	Serum	Human	86	Within 24 hr post stroke	Decreased	NA	Therapy
**5191**	10.1016/j.thromres.2017.09.00	Serum	Human	86	Within 24 hr post stroke	Decreased	NA	Therapy
**3186-5p**	10.1016/j.thromres.2017.09.00	Serum	Human	86	Within 24 hr post stroke	Decreased	NA	Therapy
**15a-5p**	10.1016/j.thromres.2017.09.00	Serum	Human	86	Within 24 hr post stroke	Decreased	NA	Therapy
**4773**	10.1016/j.thromres.2017.09.00	Serum	Human	86	Within 24 hr post stroke	Decreased	NA	Therapy
**4510**	10.1016/j.thromres.2017.09.00	Serum	Human	86	Within 24 hr post stroke	Decreased	NA	Therapy
**411-5p**	10.1016/j.thromres.2017.09.00	Serum	Human	86	Within 24 hr post stroke	Decreased	NA	Therapy
**106a-5p**	10.1016/j.thromres.2017.09.00	Serum	Human	86	Within 24 hr post stroke	Decreased	NA	Therapy
**exosomal 139**	https://doi.org/10.7150%2Fthno.56367	BV2 microglial cells, ReN cells, brain tissue	animal , in vitro	16	collected at 24 hours post-onset	*pathobiological study/intervention	anti-inflammatory	Therapy
**exosomal 139-5p**	https://doi.org/10.7150%2Fthno.56367	brain tissue, ReN cells, BV2 cells, microglia	animal , in vitro	N/A	collected 24 hours post-reperfusion	increased	anti-inflammatory	Therapy
**126**	https://doi.org/10.1016/j.omtn.2019.02.002	brain tissue, HUVECs	animal , in vitro	60	N/A	decreased	promote angiogenesis/neurogenesis	Therapy
	https://doi.org/10.1186/1471-2377-13-178	plasma	human	197 (50 control)	collected at 24 hours, 1/4/24/48 weeks post-onset	decreased	N/A	Diagnostic Biomarker
	DOI: 10.4103/0028-3886.310100	serum	human	106 (80 control)	collected 3/6/12 hours post-onset	decreased	angiogenesis, anti-inflammatory	Diagnostic Biomarker
	DOI: 10.26355/eurrev_201802_14394	brain tissue, EPCs	animal	30	N/A	*pathobiological study/intervention	angiogenesis	Therapy

	https://doi.org/10.1590/0004-282X20190126	brain tissue, blood	animal	50	collected 48 hours post-reperfusion	increased (>2 fold)	apoptosis	Prognosis
	https://doi.org/10.1155/2018/2912347	brain tissue, EPCs	animal , in vitro	40	collected 3 days post-onset	*pathobiological study/intervention	anti-oxidative, angiogenesis	Therapy
**126-3p**	https://doi.org/10.1161/STROKEAHA.119.027531	brain tissue	animal	N/A	collected at 1 and 3 days post-onset	decreased	decrease blood brain barrier disruption, leukocyte infiltration, anti-inflammatory	Therapy
**exosomal 126**	PMID: 30899379	ADSCs, plasma, brain tissue, microglia	human,animal, in vitro	13 (17 control)	collected at 4/12/24 hours post-onset	decreased	enhance neurogenesis, inhibit neuroinflammation	Therapy
	https://doi.org/10.1186%2F1471-2377-13-178	Serum	human	197	Within 24 hr post stroke	Decreased	NA	Diagnostic Biomarker
**126**	https://doi.org/10.1007/s12975-015-0429-3	serum, brain endothelial cells	animal , in vitro	59	collected 0/3/24 hours post-onset	decreased (normalized at 24 hours)	pathogenesis	Diagnostic Biomarker / Prognosis/ Therapy
	https://doi.org/10.1016/j.omtn.2020.04.008	serum, SH-SY5Y neuronal cells	human, in vitro	4	N/A	*pathobiological study/intervention	neuroprotection, increase tolerance	Therapy
**493**	https://doi.org/10.1111/febs.13697	brain tissue	animal	5	collected 7 days post-onset	decreased (in recovery phase)	anti angiogenesis	Therapy
**26a**	10.26355/eurrev_201806_15175	brain tissue, BMECs	animal , in vitro	48	collected 1/3/7 days post-onset in vivo / 2/4/6 hours post-onset in vitro	increased	promote angiogenesis	Therapy
	https://doi.org/10.1080%2F21623945.2021.1938829	adipose mesenchymal stem cells, brain tissue	animal , in vitro	N/A	collected 72 hours post-reperfusion	*pathobiological study/intervention	anti-apoptosis, neurite regeneration	Therapy
**exosomal 26a**	DOI: 10.1111/jcmm.14774	brain tissue, neural stem cells, urine-derived stem cells	animal , in vitro	40	collected 14 days post-onset	*pathobiological study/intervention	proliferation, neuronal differentiation	Therapy
**381-3p**	https://doi.org/10.1007/s10571-020-00815-4	brain tissue, endothelial progenitor cells, serum	animal , in vitro	20	collected 50 hours post-onset	decreased	anti-inflammatory, promote angiogenesis/cell proliferation	Therapy
	https://doi.org/10.3389/fnins.2021.738576	plasma	animal	3	collected 3 hours post-onset	decreased (3.53 fold)	regulated proteolysis and chemokine signaling	Diagnostic Biomarker
**exosomal 22-3p**	https://doi.org/10.1186/s13287-020-02091-x	adipose tissue, brain tissue	animal , in vitro	N/A	N/A	*pathobiological study/intervention	anti-apoptosis	Therapy
**222**	10.1007/s10072-017-3071-x	Plasma	Human	106	Without 24 hr post stroke	Increased	NA	Diagnostic and prognostic
**25**	https://doi.org/10.3390/ijms14012072	blood	human	8	collected 2-24 months post-onset	decreased	pathogenesis	Diagnostic Biomarker/Prognosis
**218**	10.1007/s10072-017-3071-x	Plasma	Human	106	Without 24 hr post stroke	Increased	NA	Diagnostic and prognostic
**185**	10.1007/s10072-017-3071-x	Plasma	Human	106	Without 24 hr post stroke	Increased	NA	Diagnostic and prognostic
**206**	10.1007/s10072-017-3071-x	Plasma	Human	106	Without 24 hr post stroke	Increased	NA	Diagnostic and prognostic
**126**	10.1007/s10072-017-3071-x	Plasma	Human	106	Without 24 hr post stroke	decreased	NA	Diagnostic and prognostic
**130a**	10.1007/s10072-017-3071-x	Plasma	Human	106	Without 24 hr post stroke	decreased	NA	Diagnostic and prognostic
**378**	10.1007/s10072-017-3071- x	Plasma	Human	106	Without 24 hr post stroke	decreased	NA	Diagnostic and prognostic
**101**	10.1007/s10072-017-3071-x	Plasma	Human	106	Without 24 hr post stroke	decreased	NA	Diagnostic and prognostic
**exosomal 25**	https://doi.org/10.1002/jev2.12024	adipose mesenchymal stem cells, brain tissue	animal , in vitro	30	collected 1 day post-onset	*pathobiological study/intervention	anti-autophagy	Therapy
**149**	https://doi.org/10.1080%2F15384101.2020.1743912	human umbilical cord mesenchymal stem cells, microglia	animal	124	collected post 24 hours of reperfusion	*pathobiological study/intervention	anti-apoptosis, anti-inflammatory	Diagnostic Biomarker/Therapy
	https://doi.org/10.3390/ijms14012072	blood	human	8	collected 2-24 months post-onset	increased	pathogenesis, inflammation, thrombosis	Diagnostic Biomarker/Prognosis

**exosomal 31**	https://doi.org/10.1016/j.expneurol.2021.113611	adiposed derived stem cells, brain tissue	animal , in vitro	60	collected 1/3/7/14 days post-onset	*pathobiological study/intervention	anti-apoptosis	Therapy
**exosomal 34c**	https://doi.org/10.1016/j.brainresbull.2020.07.013	astrocytes, brain tissue	animal , in vitro	140	collected post 24 hours of reperfusion	*pathobiological study/intervention	anti-apoptosis, promote cell proliferation	Therapy
**92b**	https://doi.org/10.1111/jcmm.16537	brain tissue, BMECs	animal , in vitro	127	collected 0/4/6/8/10 hours post-onset	decreased	promote integrity of blood brain barrier	Therapy
**92b-3p**	https://doi.org/10.1155/2022/3494262	brain tissue, plasma, SH-SY5Y cells	animal , in vitro	60	collected at 24 hours post-reperfusion	decreased	inhibit apoptosis, oxidative stress	Therapy
	https://doi.org/10.1113/EP088708	PC12 cells	in vitro	N/A	N/A	decreased	anti-apoptosis, anti-inflammation, decrease mitochondrial dysfunction	Therapy
	https://doi.org/10.3389/fnins.2021.738576	plasma	animal	3	collected 3 hours post-onset	increased (0.85 fold)	regulated proteolysis and chemokine signaling	Diagnostic Biomarker
	https://doi.org/10.3389/fgene.2019.00814	brain tissue (Day 1 data)	animal	30	collected days 1/3/7/14/28 post-onset	decreased (-0.734214924 fold)	regulate inflammation, immune responses, and angiogenesis	Diagnostic Biomarker
		brain tissue (Day 28 data)	animal	30	collected days 1/3/7/14/28 post-onset	increased (1.859484489 fold)	regulate synapses, cognition, axonogenesis, and ion transmembrane transport	Diagnostic Biomarker
**exosomal 92b-3p**	https://doi.org/10.1016/j.brainres.2019.04.009	astrocytes, brain tissue	in vitro	N/A	N/A	*pathobiological study/intervention	anti-apoptosis	Therapy
**98**	https://doi.org/10.3389/fcell.2020.00632	brain tissue	animal	N/A	collected 72 hours post-onset	decreased	anti-inflammatory	Prognosis
**exosomal 98**	https://doi.org/10.1038/s41419-020-03310-2	serum, brain tissue	human, animal, in vitro	10 patients	collected 1/3/5 days, 1/2/3/4/5 weeks post-onset in animals / collected 0.5/1/3/5 hours post-onset in vitro	decreased	decrease microglia phagocytosis and neuronal death, anti-inflammatory	Diagnostic Biomarker/Prognosis/Therapy
	doi:10.7150/thno.56367	BV2 microglial cells, ReN cells, brain tissue	animal , in vitro	16	collected at 24 hours post-onset	*pathobiological study/intervention	anti-inflammatory	Therapy
**124-3p**	https://doi.org/10.1016/j.gendis.2019.01.002	brain tissue	animal	N/A	collected 1/3/6/12/24 hours post-onset	decreased (hour 1/6/12/24)	neuroprotection	Therapy
	https://doi.org/10.2147/IJGM.S327594	whole blood, plasma, mononuclear cells	human	118 (74 control)	N/A	increased	inflammation and immune response	Diagnostic Biomarker/Therapy
	https://doi.org/10.3389/fgene.2019.00814	brain tissue (Day 14 data)	animal	30	collected days 1/3/7/14/28 post-onset	decreased (-0.417519555 fold)	regulate inflammation, immune responses, and angiogenesis	Diagnostic Biomarker
		brain tissue (Day 28 data)	animal	30	collected days 1/3/7/14/28 post-onset	decreased (-0.626141475 fold)	regulate inflammation, immune responses, and angiogenesis	Diagnostic Biomarker
	https://doi.org/10.1016/j.cca.2014.03.007	plasma	humnan	74	0–6 hr after onset	decreased	NA	Diagnostic Biomarker
**exosomal 124-3p**	https://doi.org/10.3389/fmolb.2021.685088	serum, microglial cell line	human, in vitro	10	collected 2/4/6 hours post-onset	decreased	anti-apoptosis, anti-inflammatory	Diagnostic Biomarker/Prognosis
	https://doi.org/10.3389/fnmol.2022.874903	serum, brain tissue	human, animal	40 (33 control) patients	collected within 18.5 hours post-admission in patients / collected 1 day post-onset in animals	increased	blood brain barrier disruption	Diagnostic Biomarker/Prognosis
**132/212 cluster**	https://doi.org/10.1038/s41420-021-00773-w	brain tissue	animal , in vitro	N/A	collected 24 hours post-reperfusion onset	increased	enhance blood brain barrier integrity	Therapy
**exosomal 132**	10.3389/fcell.2020.568304	bone mesenchymal stem cells, brain tissue	animal	96	collected 1 week post-onset	*pathobiological study/intervention	anti-apoptosis	Therapy
**exosomal 134**	https://doi.org/10.1186/s12883-018-1196-z	serum	human	100	collected within 24 hours post-onset	increased	pro-inflammatory	Diagnostic Biomarker/Prognosis

**exosomal 135a-5p**	https://doi.org/10.1038/s41374-021-00545-1	microglia, brain tissue	animal , in vitro	60	N/A	*pathobiological study/intervention	anti-apoptosis, anti-autophagy, promote cell proliferation	Therapy
**135b**	https://doi.org/10.3892/etm.2020.8628	serum, PC12 cells	human, in vitro	76 (60 control) patients	collected on day of and 14 days after admission	increased	cell death, regulate TRPC6 overexpression	Diagnostic Biomarker/Prognosis/Therapy
**138a-5p**	https://doi.org/10.2147/IJGM.S327594	whole blood, plasma, mononuclear cells	human	118 (74 control)	N/A	increased	inflammation and immune response	Diagnostic Biomarker/Therapy
**exosomal 138-5p**	https://doi.org/10.1186/s13036-019-0193-0	bone marrow mesenchymal stem cells, brain tissue, astrocytes	animal , in vitro	40	collected 4 weeks post-treatment	*pathobiological study/intervention	anti-apoptosis, anti-inflammatory, promote cell proliferation	Therapy
**exosomal 146a-5p**	https://doi.org/10.18632%2Faging.202466	umbilical cord mesenchymal stem cells, brain tissue	animal , in vitro	36	collected 72 hours post-reperfusion	*pathobiological study/intervention	anti-inflammatory	Therapy
	https://doi.org/10.1186/s13287-021-02668-0	brain tissue, ADSCs, microglia	animal, in vitro	28	collected 7 and 14 days post-onset	*pathobiological study/intervention	promoted M2 polarization of microglia, anti-inflammatory	Therapy
**223-3p**	https://doi.org/10.3390/ijms23010161	serum, brain tissue, spleen, liver	animal	N/A	collected 1/6/12/24/72 hours post-onset	increased	inflammation	Therapy
	DOI: 10.21307/ane-2019-018	blood, brain tissue	animal	24	collected at 4 hours post-onset	increased (>1.5 fold)	diagnostic of hyperacute stage	Diagnostic Biomarker
	https://doi.org/10.3389/fgene.2019.00814	brain tissue (Day 1 data)	animal	30	collected days 1/3/7/14/28 post-onset	increased (1.14233685 fold)	regulate synapses, cognition, axonogenesis, and ion transmembrane transport	Diagnostic Biomarker
		brain tissue (Day 3 data)	animal	30	collected days 1/3/7/14/28 post-onset	increased (1.756268911 fold)	regulate synapses, cognition, axonogenesis, and ion transmembrane transport	Diagnostic Biomarker
		brain tissue (Day 7 data)	animal	30	collected days 1/3/7/14/28 post-onset	increased (2.79246928 fold)	regulate synapses, cognition, axonogenesis, and ion transmembrane transport	Diagnostic Biomarker
		brain tissue (Day 14 data)	animal	30	collected days 1/3/7/14/28 post-onset	increased (2.204677733 fold)	regulate synapses, cognition, axonogenesis, and ion transmembrane transport	Diagnostic Biomarker
		brain tissue (Day 28 data)	animal	30	collected days 1/3/7/14/28 post-onset	increased (1.810494592 fold)	regulate synapses, cognition, axonogenesis, and ion transmembrane transport	Diagnostic Biomarker
**exosomal 223-3p**	https://doi.org/10.1016/j.lfs.2020.118403	mesenchymal stem cells, brain tissue	animal , in vitro	24	collected 28 days post-reperfusion	*pathobiological study/intervention	anti-inflammatory	Prognosis
	https://doi.org/10.1186/s13287-021-02668-0	brain tissue, ADSCs, microglia	animal, in vitro	28	collected 7 and 14 days post-onset	*pathobiological study/intervention	promoted M2 polarization of microglia, anti-inflammatory	Therapy
**exosomal 1290**	https://doi.org/10.1038/s41419-019-2100-5	brain tissue, HUVECs	animal , in vitro	N/A	N/A	*pathobiological study/intervention	anti-apoptosis	Therapy
**exosomal 27-3p**	https://doi.org/10.1007/s10753-020-01399-3	serum, brain tissue, microglia	human, animal, in vitro	43 (43 control) patients, 72 rats	collected 2 days post-onset in animals	*pathobiological study/intervention	pro-inflammatory	Diagnostic Biomarker
**27a-3p**	https://doi.org/10.18632/aging.202866	brain tissue, HT22 cells	animal, in vitro	36	N/A	decreased	anti-apoptosis	Therapy
**27a**	https://doi.org/10.3390/ijms15011418	peripheral blood	human, animal	169 (24 control)	collected at 0-7 days post-onset	increased (5.37 fold)	neurogenesis, acute phase determinant	Diagnostic Biomarker
**exosomal 27a**	https://doi.org/10.1161/STROKEAHA.120.031728	brain tissue, endothelial cells	animal , in vitro	8	N/A	increased	promote axonal growth, homeostasis, and plasticity	Therapy
**181b**	https://doi.org/10.1007/s12035-020-02018-w	whole blood, plasma, SH-SY5Y cells	human, animal, in vitro	48 patients, 10 (10	collected within 3 hours post-delivery in patients / collected at 0/1/2/8/72 hours post-onset in animals	increased (resolves after 1 hour)	neuronal injury	Diagnostic Biomarker

				control) piglets				
**181b-5p**	https://doi.org/10.1177/0271678X19858637	brain tissue	animal , in vitro	N/A	collected at 24 hours post-onset	decreased (>1.5-fold)	anti-neuroinflammation	Therapy
	https://doi.org/10.3389/fgene.2019.00814	brain tissue (Day 7 data)	animal	30	collected days 1/3/7/14/28 post-onset	increased (0.659051598 fold)	regulate synapses, cognition, axonogenesis, and ion transmembrane transport	Diagnostic Biomarker
**exosomal 133b**	https://doi.org/10.1002/stem.1129	mesenchymal stromal cells, brain tissue, astrocytes	animal , in vitro	18	collected 4 days post-onset	*pathobiological study/intervention	promotes neurite outgrowth	Therapy
	https://doi.org/10.1002/stem.1409	mesenchymal stromal cells, brain tissue	animal	51	collected 14 days post-onset	*pathobiological study/intervention	promote axon plasticity, neurite remodeling	Therapy
**34a**	https://doi.org/10.1038/s41598-020-59997-y	brain tissue, primary cerebrovascular endothelial cells	animal	165	collected 6 and 24 hours post-onset	increased	disrupts blood brain barrier permeability	Therapy
	https://doi.org/10.1016/j.neuint.2018.10.019	serum, brain tissue	human, animal, in vitro	13 (15 control)	collected within 6/24 hours/5/30/90 days post-onset of symptoms	increased	disrupts blood brain barrier permeability, pro-apoptosis	Therapy
	DOI: 10.26355/eurrev_201909_19021	brain tissue	animal	60	collected 2 days post-onset	increased	apoptosis	Prognosis
**34a-5p**	DOI: 10.12659/MSM.900237	blood, brain tissue	human, animal	102 (97 control) patients, 20 mice	collected within 72 hours post-onset	increased	apoptosis	Diagnostic Biomarker
	https://doi.org/10.3389/fgene.2019.00814	brain tissue (Day 1 data)	animal	30	collected days 1/3/7/14/28 post-onset	decreased (-0.540725336 fold)	regulate inflammation, immune responses, and angiogenesis	Diagnostic Biomarker
**451a**	https://doi.org/10.3389/fimmu.2020.00759	peripheral blood, PBMCs, natural killer cells	human, in vitro	28	collected 3/7-10 days post-onset	increased (>2fold)	impair NK-cell mediated immune defense	Diagnostic Biomarker
	https://doi.org/10.3390/ijms23063387	serum	human	95 (46 control)	collected within 24 hours of diagnosis	increased (2.27 fold)	neuroinflammation	Diagnostic Biomarker/Prognosis
	https://doi.org/10.3390/ijms21207615	serum	human	78 (20 control)	collected at admission, 24/72 hours post-onset	increased	anti-angiogenesis, neuronal damage	Diagnostic Biomarker/Prognosis
	https://doi.org/10.3390/jcm8020130	serum	human	36 (20 control)	collected at 0/24/48 hours after admission	increased	anri-angiogenesis	Diagnostic Biomarker / Prognosis/ Therapy
	https://doi.org/10.3389/fgene.2019.00814	brain tissue (Day 1 data)	animal	30	collected days 1/3/7/14/28 post-onset	increased (3.599711257 fold)	regulate synapses, cognition, axonogenesis, and ion transmembrane transport	Diagnostic Biomarker
		brain tissue (Day 3 data)	animal	30	collected days 1/3/7/14/28 post-onset	increased (2.995155921 fold)	regulate synapses, cognition, axonogenesis, and ion transmembrane transport	Diagnostic Biomarker
		brain tissue (Day 28 data)	animal	30	collected days 1/3/7/14/28 post-onset	decreased (-5.2311144 fold)	regulate inflammation, immune responses, and angiogenesis	Diagnostic Biomarker
**exosomal 451a**	https://doi.org/10.1111/cns.13612	plasma, Neuro-2a cells, brain tissue	animal , in vitro	N/A	collected 24 hours post-onset	*pathobiological study/intervention	antioxidant, anti-inflammatory, and anti-apoptotic	Therapy
	https://doi.org/10.1186/s13287-021-02668-0	brain tissue, ADSCs, microglia	animal, in vitro	28	collected 7 and 14 days post-onset	*pathobiological study/intervention	promoted M2 polarization of microglia, anti-inflammatory	Therapy
**122-5p**	https://doi.org/10.3389/fimmu.2020.00759	peripheral blood, PBMCs, natural killer cells	human, in vitro	28	collected 3/7-10 days post-onset	increased (>2fold)	impair NK-cell mediated immune defense	Diagnostic Biomarker
	https://doi.org/10.1093/hmg/ddy136	serum, brain tissue, lymphoblastoid &	human, animal, in vitro	44 (21 control) patients	N/A	increased	pathogenesis	Diagnostic Biomarker

		neuroblastoma cell lines						
**exosomal 17-92 cluster**	https://doi.org/10.1161/strokeaha.116.015204	mesenchymal stromal cells , brain tissue	animal , in vitro	N/A	collected 28 days post-onset	*pathobiological study/intervention	promote neurogenesis, oligodendrogenesis, neurite remodeling	Therapy
**422a**	https://doi.org/10.3390/ijms15011418	peripheral blood	human, animal	169 (24 control)	collected at 0-7 days post-onset	increased (1.52 fold)	acute phase determinant	Diagnostic Biomarker
**exosomal 15a**	https://doi.org/10.3390/biomedicines9070786	serum	human	81 (22 control)	collected within 24 hours post-onset	decreased	differentiate stroke subgroups, angiogenesis	Diagnostic Biomarker/Prognosis/Therapy
**exosomal 100**		serum	human	81 (22 control)	collected within 24 hours post-onset	decreased	differentiate stroke subgroups, angiogenesis	Diagnostic Biomarker/Prognosis/Therapy
**3552**	https://doi.org/10.1155/2020/4501393	blood, brain tissue	animal	14	collected 24 hours post-reperfusion	decreased	regulate apoptosis	Diagnostic Biomarker
	https://doi.org/10.3390/ijms18112335	brain tissue, whole blood	animal	14	collected 24 hours post-reperfusion	increased (1.52 fold)	regulate pathogenesis	Diagnostic Biomarker
	https://doi.org/10.3389/fgene.2019.00814	brain tissue (Day 7 data)	animal	30	collected days 1/3/7/14/28 post-onset	decreased (-2.6507523 fold)	regulate inflammation, immune responses, and angiogenesis	Diagnostic Biomarker
**484**	https://doi.org/10.1080/21655979.2021.1898134	brain tissue	animal	N/A	collected 0/12/24/48 hours post-onset	decreased	anti-apoptosis, increased cell viability	Therapy
	https://doi.org/10.3389/fgene.2019.00814	brain tissue (Day 1 data)	animal	30	collected days 1/3/7/14/28 post-onset	decreased (-1.614488459 fold)	regulate inflammation, immune responses, and angiogenesis	Diagnostic Biomarker
**497**	https://doi.org/10.1080/21655979.2021.1940073	serum, brain tissue	human, animal	89 (39 control) patients, 30 rats	collected at admission and discharge in patients / 24 hours post-onset in animals	decreased	anti-apoptosis	Diagnostic Biomarker/Prognosis/Therapy
**374**	https://doi.org/10.1538/expanim.20-0034	brain tissue	animal	186	collected 6/24/72 hours post-onset	decreased	anti-apoptosis, decreased edema & blood brain barrier disruption	Therapy
**320b**	https://doi.org/10.1186/s12881-020-00994-3	plasma	human	31 (28 control)	N/A	increased	pathogenesis through neurotrophin signaling	Diagnostic Biomarker
**320d**	https://doi.org/10.1186/s12881-020-00994-3	plasma	human	32 (28 control)	N/A	increased	pathogenesis through neurotrophin signaling	Diagnostic Biomarker
	https://doi.org/10.1016/j.jstrokecerebrovasdis.2014.06.002	plasma	human	136 (116 control)	collected 0-3/3-6/6-12/12-24 hours post-onset	decreased (0.07-0.23 fold)	N/A	Diagnostic Biomarker
	https://doi.org/10.1371/journal.pone.0099283	peripheral blood	human	24 (24 control)	collected at 72 hours post-onset	decreased (1.70 fold)	inflammation, immune regulation, cell proliferation	Diagnostic Biomarker/Prognosis
**320e**	https://doi.org/10.1016/j.jstrokecerebrovasdis.2014.06.002	plasma	human	136 (116 control)	collected 0-3/3-6/6-12/12-24 hours post-onset	decreased (0.13-0.33 fold)	N/A	Diagnostic Biomarker
**186-5p**	https://doi.org/10.7150/ijbs.25352	serum, SH-SY5Y cells	human, in vitro	23 (23 control)	collected within 5 days of onset	increased	pro-apoptosis	Diagnostic Biomarker/Therapy
**186-5p**	https://doi.org/10.1007/s12035-018-1295-2	Serum	Human	11	Within 24 hr post stroke	decreased	NA	Therapy
	https://doi.org/10.3389/fgene.2019.00814	brain tissue (Day 1 data)	animal	30	collected days 1/3/7/14/28 post-onset	increased (0.612769922 fold)	regulate synapses, cognition, axonogenesis, and ion transmembrane transport	Diagnostic Biomarker
**367-3p**	https://doi.org/10.1177/0271678X19858637	brain tissue	animal , in vitro	N/A	collected at 24 hours post-onset	decreased (10.73 fold)	anti-neuroinflammation	Therapy
**302b-3p**	https://doi.org/10.1177/0271678X19858637	brain tissue	animal , in vitro	N/A	collected at 24 hours post-onset	decreased (1.87 fold)	anti-neuroinflammation	Therapy
**302e**	https://doi.org/10.3390/ijms14012072	blood	human	8 (4 control)	collected 2-24 months post-onset	increased	pathogenesis, inflammation, thrombosis	Diagnostic Biomarker/Prognosis
**296-5p**	https://doi.org/10.3390/ijms14012072	brain tissue	animal , in vitro	N/A	collected at 24 hours post-onset	decreased (2.54 fold)	anti-neuroinflammation	Therapy
	https://doi.org/10.3389/fnmol.2014.00011	brain tissue	animal	N/A	collected at 8 hours post-onset	decreased	CD73/PKN2 targets/mechanisms	Prognosis/Therapy

	https://doi.org/10.3389/fgene.2019.00814	brain tissue (Day 1 data)	animal	30	collected days 1/3/7/14/28 post-onset	decreased (-1.545178897 fold)	regulate inflammation, immune responses, and angiogenesis	Diagnostic Biomarker
**489-3p**	https://doi.org/10.3390/ijms14012072	brain tissue	animal , in vitro	N/A	collected at 24 hours post-onset	decreased (2.47 fold)	anti-neuroinflammation	Therapy
**224-5p**	https://doi.org/10.3390/ijms14012072	brain tissue	animal , in vitro	N/A	collected at 24 hours post-onset	decreased (3.47 fold)	anti-neuroinflammation	Therapy
	https://doi.org/10.3389/fnins.2020.00613	brain tissue	animal , in vitro	N/A	collected at 12/24/48 hours post-onset	increased	apoptosis	Therapy
	https://doi.org/10.3389/fgene.2019.00814	brain tissue (Day 3 data)	animal	30	collected days 1/3/7/14/28 post-onset	increased (4.700175298 fold)	regulate synapses, cognition, axonogenesis, and ion transmembrane transport	Diagnostic Biomarker
**409-3p**	https://doi.org/10.1002/kjm2.12327	serum, PC12 cells	human, in vitro	80 (30 control)	collected at 9 hours post-onset	increased	pro-apoptosis	Diagnostic Biomarker/Prognosis
	https://doi.org/10.3389/fgene.2019.00814	brain tissue (Day 7 data)	animal	30	collected days 1/3/7/14/28 post-onset	decreased (-0.857074132 fold)	regulate inflammation, immune responses, and angiogenesis	Diagnostic Biomarker
**125b-2**	https://doi.org/10.3390/ijms15011418	peripheral blood	human, animal	169 (24 control)	collected at 0-7 days post-onset	increased (1.56 fold)	acute phase determinant	Diagnostic Biomarker
**125a-5p**	https://doi.org/10.5114/fn.2021.107109	BV2 microglial cells, brain tissue	animal , in vitro	N/A	collected at 24 hours post-onset	increased	pro-apoptosis	Therapy
	https://doi.org/10.3390/ijms14012072	blood	human	8 (4 control)	collected 2-24 months post-onset	increased	pathogenesis, inflammation, thrombosis	Diagnostic Biomarker/Prognosis
	https://doi.org/10.3389/fgene.2022.833545	whole blood, HT22 cells	human, in vitro	59 (44 control)	N/A	decreased	cell viability, angiogenesis	Diagnostic Biomarker
	https://doi.org/10.3892/mmr.2020.11143	peripheral blood mononuclear cells, peripheral blood (male data)	human	260 (160 control)	N/A	*pathobiological study/intervention	inflammation, apoptosis	Diagnostic Biomarker/Therapy
	https://doi.org/10.3389/fgene.2019.00814	brain tissue (Day 7 data)	animal	30	collected days 1/3/7/14/28 post-onset	decreased (-1.376252017 fold)	regulate inflammation, immune responses, and angiogenesis	Diagnostic Biomarker
**150**	https://doi.org/10.1096/fj.201500126	BMECs, brain tissue	animal , in vitro	N/A	collected 72 hours post-onset	*pathobiological study/intervention	increase permeability of blood brain barrier, decrease cell survival rate	Therapy
	https://doi.org/10.1111/cns.12525	brain tissue, serum, BMVECs, astrocytes	animal , in vitro	N/A	collected 1/3/7 days post-onset	decreased	anti-angiogenesis	Therapy
**539**	https://doi.org/10.1007/s11064-018-2646-0	bEND.3 cells, brain tissue	animal , in vitro	12	collected at 26 hours post-onset	decreased	decrease permeability of blood brain barrier	Prognosis/Therapy
	https://doi.org/10.1155/2021/5699025	peripheral blood, brain tissue, brain endothelial cells	human, animal, in vitro	48(48 control) patients, 24 rats	N/A	decreased	blood brain barrier permeability	Diagnostic Biomarker
**149-5p**	https://doi.org/10.1096/fj.201701121R	pericytes, brain tissue, peripheral blood	animal , in vitro	N/A	collected from 12 hours to 7 days post-onset	decreased (recovered at day 5)	decrease permeability of blood brain barrier, pericyte migration	Therapy
	https://doi.org/10.1016/j.brainresbull.2021.01.013	brain tissue	animal	N/A	collected 24 hours post-onset	*pathobiological study/intervention	reduce tissue damage/edema, increase blood brain barrier integrity	Therapy
	https://doi.org/10.52547/ibj.3759	brain tissue	animal	108	collected 24 hours post-onset	decreased	anti-inflammatory and antioxidant	Therapy
	https://doi.org/10.1080/15384101.2020.1731649	brain tissue, pericytes	animal	60	collected 12 hours/1/3/5/7 days post-onset	*pathobiological study/intervention	decrease blood brain barrier permeability	Therapy
**668**	https://doi.org/10.5114/fn.2020.94003	brain tissue	animal	N/A	collected at 24 hours post-onset	*pathobiological study/intervention	pro-apoptosis, pro-inflammatory, increase	Therapy

							permeability of blood brain barrier	
**182**	https://doi.org/10.1096/fj.201903092R	bEND.3 cells, brain tissue, astrocytes, pericytes, N2a/BV2 cells	animal , in vitro	250	collected at 2/4/6/12/24/72 hours post-onset	increased	pro-apoptosis, promote blood brain barrier damage	Therapy
	https://doi.org/10.3892/mmr.2019.10073	brain tissue	animal	20	N/A	increased	involved in development of ischemic stroke	Diagnostic Biomarker
**503**	https://doi.org/10.1007/s12975-020-00794-0	plasma, brain tissue, endothelial cells	human, animal, in vitro	132 (81 control) patients, 348 mice	collected at <6/6-24 hours/24 hours-2 weeks/ >2 weeks post-onset	increased	pro-apoptosis, promote blood brain barrier disruption	Diagnostic Biomarker/Therapy
	https://doi.org/10.1007/s12035-016-0347-8	peripheral blood mononuclear cells	human	20 (19 control)	collected within 48 hours of onset	increased (+/- 1.5 fold)	pro-inflammatory	Diagnostic Biomarker/Prognosis
**let-7f**	https://doi.org/10.1016/j.brainres.2021.147662	bEND.3 cells	in vitro	N/A	collected 48-54 hours post-onset	*pathobiological study/intervention	decrease blood brain barrier permeability, anti-apoptosis	Therapy
	https://doi.org/10.1371/journal.pone.0032662	brain tissue, microglia	animal , in vitro	74	collected 5 days post-onset	*pathobiological study/intervention	anti-neuroprotection	Therapy
**141-3p**	https://doi.org/10.3390/cells10051011	brain tissue	animal	26	collected 48-72 hours post-onset	*pathobiological study/intervention	pro-inflammatory, increase infarct injury	Therapy
	https://doi.org/10.3389/fgene.2019.00814	brain tissue (Day 3 data)	animal	30	collected days 1/3/7/14/28 post-onset	increased (4.820480247 fold)	regulate synapses, cognition, axonogenesis, and ion transmembrane transport	Diagnostic Biomarker
		brain tissue (Day 28 data)	animal	30	collected days 1/3/7/14/28 post-onset	increased (1.66368214 fold)	regulate synapses, cognition, axonogenesis, and ion transmembrane transport	Diagnostic Biomarker
**183**	https://doi.org/10.3892/etm.2019.7827	brain tissue	animal	36	N/A	decreased	anti-inflammatory, regulate microglia activation	Therapy
**183-5p**	https://doi.org/10.3892/mmr.2020.11493	brain tissue, neuroblastoma cells	animal , in vitro	112	collected 25 hours post-onset	decreased	anti-apoptosis	Therapy
	https://doi.org/10.3892/mmr.2019.10073	brain tissue	animal	20	N/A	increased	involved in development of ischemic stroke	Diagnostic Biomarker
	https://doi.org/10.3389/fgene.2019.00814	brain tissue (Day 3 data)	animal	30	collected days 1/3/7/14/28 post-onset	increased (2.208502773 fold)	regulate synapses, cognition, axonogenesis, and ion transmembrane transport	Diagnostic Biomarker
		brain tissue (Day 7 data)	animal	30	collected days 1/3/7/14/28 post-onset	increased (3.799079779 fold)	regulate synapses, cognition, axonogenesis, and ion transmembrane transport	Diagnostic Biomarker
**196a**	DOI: 10.26355/eurrev_201901_16888	brain tissue	animal , in vitro	N/A	collected 25 hours post-onset	increased	pro-apoptosis	Therapy
**1258**	https://doi.org/10.3390/ijms14012072	blood	human	8 (4 control)	collected 2-24 months post-onset	increased	pathogenesis, inflammation, thrombosis	Diagnostic Biomarker/Prognosis
**1260**		blood	human	8 (4 control)	collected 2-24 months post-onset	increased	pathogenesis, inflammation, thrombosis	Diagnostic Biomarker/Prognosis
**1273**		blood	human	8 (4 control)	collected 2-24 months post-onset	increased	pathogenesis, inflammation, thrombosis	Diagnostic Biomarker/Prognosis
**220b**		blood	human	8 (4 control)	collected 2-24 months post-onset	increased	pathogenesis, inflammation, thrombosis	Diagnostic Biomarker/Prognosis
**23a**		blood	human	8 (4 control)	collected 2-24 months post-onset	increased	pathogenesis, inflammation, thrombosis	Diagnostic Biomarker/Prognosis
**23b**	doi:10.7150/ijbs.61399	blood, brain tissue	human, animal, in vitro	40 (25 control) patients, 105 mice	collected within 24-48 hours post-onset	increased	pro-apoptosis	Therapy
**exosomal 23a-5p**	doi:10.7150/thno.68895	brain tissue, microglia	animal, in vitro	122	collected at 14 and 28 days post-onset	*pathobiological study/intervention	promote oligodendrogenesis, white matter remodeling	Therapy

**26b**	https://doi.org/10.3390/ijms14012072	blood	human	8 (4 control)	collected 2-24 months post-onset	increased	pathogenesis, inflammation, thrombosis	Diagnostic Biomarker/Prognosis
**exosomal 26b-5p**	https://doi.org/10.1080/15384101.2020.1743912	brain tissue, hUCMSCs,microglia	animal, in vitro	124	collected 24 hours post-reperfusion	decreased	anti-apoptosis, anti-inflammatory	Therapy
**488**	https://doi.org/10.3390/ijms14012072	blood	human	8 (4 control)	collected 2-24 months post-onset	increased	pathogenesis, inflammation, thrombosis	Diagnostic Biomarker/Prognosis
	https://doi.org/10.3390/ijms15011418	peripheral blood	human, animal	169 (24 control) patients	collected at 0-7 days post-onset	increased (1.36 fold)	acute phase determinant	Diagnostic Biomarker
**490-3p**	https://doi.org/10.3390/ijms14012072	blood	human	8 (4 control)	collected 2-24 months post-onset	increased	pathogenesis, inflammation, thrombosis	Diagnostic Biomarker/Prognosis
**506**		blood	human	8 (4 control)	collected 2-24 months post-onset	increased	pathogenesis, inflammation, thrombosis	Diagnostic Biomarker/Prognosis
**659**		blood	human	8 (4 control)	collected 2-24 months post-onset	increased	pathogenesis, inflammation, thrombosis	Diagnostic Biomarker/Prognosis
**890**		blood	human	8 (4 control)	collected 2-24 months post-onset	increased	pathogenesis, inflammation, thrombosis	Diagnostic Biomarker/Prognosis
**920**		blood	human	8 (4 control)	collected 2-24 months post-onset	increased	pathogenesis, inflammation, thrombosis	Diagnostic Biomarker/Prognosis
**934**		blood	human	8 (4 control)	collected 2-24 months post-onset	increased	pathogenesis, inflammation, thrombosis	Diagnostic Biomarker/Prognosis
**34b**		blood	human	8 (4 control)	collected 2-24 months post-onset	decreased	pathogenesis	Diagnostic Biomarker/Prognosis
**483-5p**	https://doi.org/10.3390/ijms14012072	blood	human	8 (4 control)	collected 2-24 months post-onset	decreased	pathogenesis	Diagnostic Biomarker/Prognosis
	https://doi.org/10.3389/fgene.2019.00814	brain tissue (Day 14 data)	animal	30	collected days 1/3/7/14/28 post-onset	increased (1.543098279 fold)	regulate synapses, cognition, axonogenesis, and ion transmembrane transport	Diagnostic Biomarker
		brain tissue (Day 28 data)	animal	30	collected days 1/3/7/14/28 post-onset	increased (2.040625645 fold)	regulate synapses, cognition, axonogenesis, and ion transmembrane transport	Diagnostic Biomarker
**498**	https://doi.org/10.3390/ijms14012072	blood	human	8 (4 control)	collected 2-24 months post-onset	decreased	pathogenesis	Diagnostic Biomarker/Prognosis
**4306**	https://doi.org/10.1016/j.jstrokecerebrovasdis.2014.06.002	plasma	human	136 (116 control)	collected 0-3/3-6/6-12/12-24 hours post-onset	increased (3.19-5.30 fold)	N/A	Diagnostic Biomarker
**627**	https://doi.org/10.3390/ijms15011418	peripheral blood	human, animal	169 (24 control) patients	collected at 0-7 days post-onset	increased (8.53 fold)	acute phase determinant	Diagnostic Biomarker
**363**	https://doi.org/10.1371/journal.pone.0099283	peripheral blood	human	24 (24 control)	collected at 72 hours post-onset	increased (3.61 fold)	inflammation, immune regulation, cell proliferation	Diagnostic Biomarker/Prognosis
**363-3p**	https://doi.org/10.1016/j.neuint.2016.10.008	brain tissue, astrocytes, serum	animal	129	collected 48 hours and 5 days post-onset	increased	anti-apoptosis	Therapy
	https://doi.org/10.3389/fnagi.2020.586362	brain tissue	animal	30	N/A	*pathobiological study/intervention	neuroprotection, reduce infarct volume	Therapy
	https://doi.org/10.3389/fgene.2019.00814	brain tissue (Day 28 data)	animal	30	collected days 1/3/7/14/28 post-onset	increased (1.677346752 fold)	regulate synapses, cognition, axonogenesis, and ion transmembrane transport	Diagnostic Biomarker
**exosomal 363-3p**	https://doi.org/10.1186/s13287-021-02668-0	brain tissue, ADSCs, microglia	animal, in vitro	28	collected 7 and 14 days post-onset	*pathobiological study/intervention	promoted M2 polarization of microglia, anti-inflammatory	Therapy
**487b**	https://doi.org/10.1371/journal.pone.0099283	peripheral blood	human	24 (24 control)	collected at 72 hours post-onset	increased (2.66 fold)	inflammation, immune regulation, cell proliferation	Diagnostic Biomarker/Prognosis
**148a**	https://doi.org/10.1371/journal.pone.0099283	peripheral blood	human	24 (24 control)	collected at 72 hours post-onset	decreased (2.05 fold)	inflammation, immune regulation, cell proliferation	Diagnostic Biomarker/Prognosis
	https://doi.org/10.1007/s12035-020-02018-w	whole blood, plasma, SH-SY5Y cells	human, animal, in vitro	48 patients, 10 (10	collected within 3 hours post-delivery in patients /	increased (resolves after 1 hour)	neuronal injury	Diagnostic Biomarker

				control) piglets	collected at 0/1/2/8/72 hours post-onset in animals			
**19a**	https://doi.org/10.1371/journal.pone.0099283	peripheral blood	human	24 (24 control)	collected at 72 hours post-onset	decreased (1.66 fold)	inflammation, immune regulation, cell proliferation	Diagnostic Biomarker/Prognosis
**exosomal 19a**	https://doi.org/10.1161/STROKEAHA.120.031728	brain tissue, endothelial cells	animal , in vitro	8	N/A	increased	promote axonal growth, homeostasis, and plasticity	Therapy
**19a-3p**	https://doi.org/10.1186/s40659-020-00280-9	brain tissue, neuroblastoma cells	animal, in vitro	18	collected 72 hours post-onset	*pathobiological study/intervention	inflammatory, apoptosis	Therapy
	https://doi.org/10.1186/s11658-019-0160-2	brain tissue, astrocytes	animal, in vitro	N/A	collected at 24 hours post-onset	increased	apoptosis	Therapy
**20a-3p**	https://doi.org/10.1007/s12975-021-00945-x	astrocytes, brain tissue	animal, in vitro	143	collected 48 hours post-onset	*pathobiological study/intervention	neuroprotection	Therapy
**20b**	https://doi.org/10.5607/en20046	brain tissue	animal, in vitro	12	collected 24 hours post-reperfusion	decreased	anti-apoptosis	Therapy
**211-5p**	https://doi.org/10.3892/mmr.2019.10073	brain tissue	animal	20	N/A	increased	involved in development of ischemic stroke	Diagnostic Biomarker
	https://doi.org/10.1093/hmg/ddy136	serum, brain tissue, lymphoblastoid & neuroblastoma cell lines	human, animal, in vitro	44 (21 control) patients	N/A	increased	pathogenesis	Diagnostic Biomarker
	https://doi.org/10.3389/fgene.2019.00814	brain tissue (Day 28 data)	animal	30	collected days 1/3/7/14/28 post-onset	increased (1.814737588 fold)	regulate synapses, cognition, axonogenesis, and ion transmembrane transport	Diagnostic Biomarker
**10b-3p**	https://doi.org/10.3892/mmr.2019.10073	brain tissue	animal	20	N/A	decreased	involved in development of ischemic stroke	Diagnostic Biomarker
**217-5p**		brain tissue	animal	20	N/A	decreased	involved in development of ischemic stroke	Diagnostic Biomarker
**96-5p**	https://doi.org/10.3892/mmr.2019.10073	brain tissue	animal	20	N/A	increased	involved in development of ischemic stroke	Diagnostic Biomarker
	https://doi.org/10.3389/fgene.2019.00814	brain tissue (Day 7 data)	animal	30	collected days 1/3/7/14/28 post-onset	increased (4.518069416 fold)	regulate synapses, cognition, axonogenesis, and ion transmembrane transport	Diagnostic Biomarker
		brain tissue (Day 28 data)	animal	30	collected days 1/3/7/14/28 post-onset	increased (1.590731003 fold)	regulate synapses, cognition, axonogenesis, and ion transmembrane transport	Diagnostic Biomarker
**544**	10.26355/eurrev_201809_15825	plasma, brain tissue, BV2 cells	human, animal, in vitro	50 (50 control) patients, 24 mice	collected > 24 hours post-onset	decreased	anti-inflammation, anti-apoptosis	Diagnostic Biomarker/Therapy
**574-5p**	https://doi.org/10.3390/ijms23063387	serum	human	95 (46 control)	collected within 24 hours of diagnosis	decreased	neuroprotection	Diagnostic Biomarker/Prognosis
	https://doi.org/10.3389/fgene.2019.00814	brain tissue (Day 14 data)	animal	30	collected days 1/3/7/14/28 post-onset	increased (0.956807736 fold)	regulate synapses, cognition, axonogenesis, and ion transmembrane transport	Diagnostic Biomarker
		brain tissue (Day 28 data)	animal	30	collected days 1/3/7/14/28 post-onset	increased (1.231927192 fold)	regulate synapses, cognition, axonogenesis, and ion transmembrane transport	Diagnostic Biomarker
**142-3p**	https://doi.org/10.3390/ijms23063387	serum	human	95 (46 control)	collected within 24 hours of diagnosis	decreased	neuroprotection	Diagnostic Biomarker/Prognosis
**411-5p**	https://doi.org/10.3390/ijms23063387	serum	human	95 (46 control)	collected within 24 hours of diagnosis	decreased	neuroprotection	Diagnostic Biomarker/Prognosis
	https://doi.org/10.3389/fnins.2021.738576	plasma	animal	3	collected 3 hours post-onset	decreased (0.86 fold)	regulated proteolysis and chemokine signaling	Diagnostic Biomarker
**379-5p**	https://doi.org/10.3390/ijms23063387	serum	human	95 (46 control)	collected within 24 hours of diagnosis	decreased	neuroprotection	Diagnostic Biomarker/Prognosis

		brain tissue (Day 28 data)	animal	30	collected days 1/3/7/14/28 post-onset	decreased (-0.864275043 fold)	regulate inflammation, immune responses, and angiogenesis	Diagnostic Biomarker
**676-3p**	https://doi.org/10.3390/ijms23063387	serum	human	95 (46 control)	collected within 24 hours of diagnosis	decreased	neuroprotection	Diagnostic Biomarker/Prognosis
**4446-3p**		serum	human	95 (46 control)	collected within 24 hours of diagnosis	decreased	neuroprotection	Diagnostic Biomarker/Prognosis
**6721-5p**		serum	human	95 (46 control)	collected within 24 hours of diagnosis	decreased	neuroprotection	Diagnostic Biomarker/Prognosis
**485-3p**	https://doi.org/10.3390/ijms23063387	serum	human	95 (46 control)	collected within 24 hours of diagnosis	decreased	neuroprotection	Diagnostic Biomarker/Prognosis
	doi:10.7150/thno.48135	brain tissue	animal	100	collected at 24 hours post-onset	increased	prevented by remote limb postconditioning	Therapy
	https://doi.org/10.3389/fgene.2019.00814	brain tissue (Day 1 data)	animal	30	collected days 1/3/7/14/28 post-onset	decreased (-0.781146801 fold)	regulate inflammation, immune responses, and angiogenesis	Diagnostic Biomarker
		brain tissue (Day 7 data)	animal	30	collected days 1/3/7/14/28 post-onset	decreased (-0.985219025 fold)	regulate inflammation, immune responses, and angiogenesis	Diagnostic Biomarker
		brain tissue (Day 14 data)	animal	30	collected days 1/3/7/14/28 post-onset	decreased (-0.762173954 fold)	regulate inflammation, immune responses, and angiogenesis	Diagnostic Biomarker
**exosomal 30d-5p**	https://doi.org/10.1159/000490078	peripheral blood, brain tissue, ADSCs	human, animal, in vitro	70 (35 control)	collected within 48 hours of hospitalization / harvested 3 hours post-onset	*pathobiological study/intervention	anti-inflammatory, anti-autophagy	Therapy
**338**	https://doi.org/10.1016/j.mito.2021.04.013	brain tissue, astrocytes	animal, in vitro	132	collected at 3hours and 1/3/5 days post-onset	increased (baseline at 3 days)	promotes cell death	Therapy
	https://doi.org/10.1038/pr.2014.104	brain tissue	animal	N/A	collected 24/48/72 hours and 7 days post-onset	increase	regulate oligodendroglial progenitor cells differentiation	Prognosis
**686**	https://doi.org/10.3389/fnmol.2014.00011	brain tissue	animal	N/A	collected at 8 hours post-onset	increased	CD73/PKN2 targets/mechanisms	Prognosis/Therapy
**187-3p**	https://doi.org/10.1016/j.neulet.2021.135947	brain tissue, PV12 cells	animal, in vitro	42	collected at 24 hours post-onset	increased	induces apoptosis	Therapy
	https://doi.org/10.3892/ijmm.2020.4642	PC12 cells, brain tissue	animal, in vitro	54	collected 24 hours post-reperfusion	increased (>2fold)	apoptosis	Therapy
	https://doi.org/10.3389/fgene.2019.00814	brain tissue (Day 14 data)	animal	30	collected days 1/3/7/14/28 post-onset	decreased (-0.605403941 fold)	regulate inflammation, immune responses, and angiogenesis	Diagnostic Biomarker
		brain tissue (Day 28 data)	animal	30	collected days 1/3/7/14/28 post-onset	decreased (-0.894852122 fold)	regulate inflammation, immune responses, and angiogenesis	Diagnostic Biomarker
**370**	https://doi.org/10.1002/kjm2.12219	brain tissue, SH-SY5Y cells	animal, in vitro	20	N/A	increased	apoptosis, decrease cell viability	Therapy
**532-3p**	https://doi.org/10.3892/mmr.2020.11325	brain tissue, plasma, SH-SY5Y cells	animal, in vitro	16	collected 26 hours post-onset	decreased	anti-apoptosis	Diagnostic Biomarker/Therapy
	https://doi.org/10.3389/fnins.2021.738576	plasma	animal	3	collected 3 hours post-onset	increased (1.07 fold)	regulated proteolysis and chemokine signaling	Diagnostic Biomarker
	https://doi.org/10.3389/fgene.2019.00814	brain tissue (Day 1 data)	animal	30	collected days 1/3/7/14/28 post-onset	decreased (-0.8921404 fold)	regulate inflammation, immune responses, and angiogenesis	Diagnostic Biomarker
**532-5p**	https://doi.org/10.18632/aging.202846	brain tissue, SH-SY5Y cells	animal, in vitro	48	collected 72 hours post-onset	decreased	anti-apoptosis, anti-inflammation	Therapy
	https://doi.org/10.1186/s12920-019-0566-8	whole blood, mononuclear cells	human	107 patients	collected within 3 hours post-onset	decreased	anoxia, inflammation, cell death	Diagnostic Biomarker/Therapy

				and controls				
	https://doi.org/10.3389/fnins.2021.738576	plasma	animal	3	collected 3 hours post-onset	decreased (1.26 fold)	regulated proteolysis and chemokine signaling	Diagnostic Biomarker
	https://doi.org/10.3389/fgene.2019.00814	brain tissue (Day 3 data)	animal	30	collected days 1/3/7/14/28 post-onset	increased (1.033401091 fold)	regulate synapses, cognition, axonogenesis, and ion transmembrane transport	Diagnostic Biomarker
**9-5p**	https://doi.org/10.6061/clinics/2021/e2958	serum	human	88 (88 control)	collected 12-14 hours after diagnosis (diagnosis within 6 hours post-onset)	increased	anti-apoptosis	Diagnostic Biomarker/Prognosis
	https://doi.org/10.1002/iub.2357	brain tissue	animal, in vitro	24	collected at 9/12/18 hours post-onset	increased	apoptosis	Therapy
	https://doi.org/10.1186/s40364-017-0104-9	CSF	human	21 (21 control)	collected 3 days post-onset	increased	apoptosis, inflammation	Diagnostic Biomarker
	https://doi.org/10.3389/fgene.2019.00814	brain tissue (Day 1 data)	animal	30	collected days 1/3/7/14/28 post-onset	increased (0.552066738 fold)	regulate synapses, cognition, axonogenesis, and ion transmembrane transport	Diagnostic Biomarker
		brain tissue (Day 3 data)	animal	30	collected days 1/3/7/14/28 post-onset	increased (0.992968133 fold)	regulate synapses, cognition, axonogenesis, and ion transmembrane transport	Diagnostic Biomarker
**429**	https://doi.org/10.1155/2021/6753926	serum, HBMECs	human, in vitro	N/A	collected 12/24/48 hours post-onset	increased	inhibit cell viability, migration, and tube formation	Therapy
**107**	https://doi.org/10.2169/internalmedicine.55.5925	plasma	human	114 (58 control)	collected within 24 hours of hospital admission	increased (2.78 fold)	positive correlation with stroke severity	Diagnostic Biomarker
**128b**	https://doi.org/10.2169/internalmedicine.55.5925	plasma	human	114 (58 control)	collected within 24 hours of hospital admission	increased (2.13 fold)	positive correlation with stroke severity	Diagnostic Biomarker
**153**	https://doi.org/10.2169/internalmedicine.55.5925	plasma	human	114 (58 control)	collected within 24 hours of hospital admission	increased (1.83 fold)	positive correlation with stroke severity	Diagnostic Biomarker
	DOI: 10.26355/eurrev_201901_16887	brain tissue	animal	40	collected at 24 hours post-onset	decreased	promote angiogenesis	Therapy
	https://doi.org/10.1155/2021/4464945	serum	human	76 (64 control)	collected within 6 hours post-onset	increased	neurological damage	Diagnostic Biomarker/Prognosis
**153-5p**	https://doi.org/10.5114/fn.2021.112127	plasma, brain tissue, Neuro-2a cells	human, animal, in vitro	68 (68 control) patients, 50 mice	collected within 6 hours of onset in patients / collected at 3/4.5 hours post-onset in animals	decreased	anti-apoptosis, enhance cell viability, anti-inflammatory	Therapy
**874**	https://doi.org/10.1007/s12035-016-0347-8	peripheral blood mononuclear cells	human	20 (19 control)	collected within 48 hours of onset	decreased (+/- 1.5 fold)	inflammation	Diagnostic Biomarker/Prognosis
**874-3p**	https://doi.org/10.1152/ajpcell.00001.2020	serum, brain tissue, HUVECs	human, animal, in vitro	28 (28 control) patients	collected 2-6 days post-onset / mouse model 24 or 48 hrs post-onset	decreased	anti-inflammatory, promotes angiogenesis	Therapy
**448-5p**	https://doi.org/10.3390/ijms23010161	serum, brain tissue, spleen, liver	animal	N/A	collected 1/6/12/24/72 hours post-onset	increased	inflammation	Therapy
**let 7a-5p**	doi:10.7150/thno.48135	brain tissue	animal	100	collected at 24 hours post-onset	increased	prevent neuroprotection	Therapy
**451-5p**	doi:10.7150/thno.48135	brain tissue	animal	100	collected at 24 hours post-onset	increased	prevented by remote limb postconditioning	Therapy
**143**	https://doi.org/10.1042/BSR20170216	brain tissue, astrocytes	animal, in vitro	N/A	collected 24/36/48 hours post-onset	increased	apoptosis	Therapy
**143-3p**	doi:10.7150/thno.48135	brain tissue	animal	100	collected at 24 hours post-onset	increased	prevent neuroprotection	Therapy
**143-3p**	https://doi.org/10.1161/circresaha.117.311572	Plasma	Human	260	5 hr post stroke	upregulated	NA	Diagnostic Biomarker
	https://doi.org/10.3389/fgene.2022.833545	whole blood, HT22 cells	human, in vitro	59 (44 control) patients	N/A	decreased	cell viability, angiogenesis	Diagnostic Biomarker

	https://doi.org/10.3892/mmr.2020.11143	peripheral blood mononuclear cells, peripheral blood (male data)	human	260 (160 control)	N/A	*pathobiological study/intervention	inflammation, apoptosis	Diagnostic Biomarker/Therapy
	https://doi.org/10.3389/fgene.2019.00814	brain tissue (Day 14 data)	animal	30	collected days 1/3/7/14/28 post-onset	increased (0.605689617 fold)	regulate synapses, cognition, axonogenesis, and ion transmembrane transport	Diagnostic Biomarker
**exosomal 190b**	https://doi.org/10.1080/15384101.2020.1731649	brain tissue, astrocytes	in vitro	N/A	N/A	*pathobiological study/intervention	anti-apoptosis and anti-autophagy	Therapy
**335**	https://doi.org/10.1177/0300060516665707	plasma	human	168 (104 control)	collected within 24 hours post-onset	decreased	regulator of pathophysiological process / suppress CaM protein expression	Diagnostic Biomarker
	https://doi.org/10.3892/ijmm.2019.4073	brain tissue, PC12 cells	animal, in vitro	108	collected 0/6/12/2436 hours post-reperfusion	decreased	anti-apoptosis, promotes stress granule formation	Therapy
	https://doi.org/10.1371/journal.pone.0128432	brain tissue	animal, in vitro	54	collected 0/3/6/12/24/48/72/120/168 hours post-onset	decreased	maintain cell viability and blood brain barrier integrity	Therapy
	https://doi.org/10.3389/fmolb.2022.914506	serum	human	60 (60 control)	collected with 24 hours of symptom onset	decreased (0.24 fold)	anti-apoptosis	Diagnostic Biomarker
**3p-57664**	https://doi.org/10.1093/hmg/ddy136	serum, brain tissue, lymphoblastoid & neuroblastoma cell lines	human, animal, in vitro	44 (21 control) patients	N/A	increased	pathogenesis	Diagnostic Biomarker
**5p-12969**	https://doi.org/10.1093/hmg/ddy136	serum, brain tissue, lymphoblastoid & neuroblastoma cell lines	human, animal, in vitro	44 (21 control) patients	N/A	increased	pathogenesis	Diagnostic Biomarker
**148b**	https://doi.org/10.3389/fncel.2017.00329	brain tissue, neural stem cells	animal, in vitro	30	collected 14 days post-onset	increased	decrease proliferation/differentiation	Therapy
**148b-3p**	https://doi.org/10.1042/BSR20181033	serum	human	77 (42 control)	collected within 30 hours post-onset	decreased	positive correlation with blood glucose	Diagnostic Biomarker
	https://doi.org/10.3389/fnins.2021.738576	plasma	animal	3	collected 3 hours post-onset	decreased (1.95 fold)	regulated proteolysis and chemokine signaling	Diagnostic Biomarker
	https://doi.org/10.3389/fgene.2022.833545	whole blood, HT22 cells	human, in vitro	59 (44 control)	N/A	decreased	cell viability	Diagnostic Biomarker
	https://doi.org/10.3389/fgene.2019.00814	brain tissue (Day 28 data)	animal	30	collected days 1/3/7/14/28 post-onset	decreased (-0.919732455 fold)	regulate inflammation, immune responses, and angiogenesis	Diagnostic Biomarker
**151b**	https://doi.org/10.1042/BSR20181033	serum	human	77 (42 control)	collected within 30 hours post-onset	increased	negative correalation with IGF-1/IGFBP3, brain development and growth	Diagnostic Biomarker
**27b**	https://doi.org/10.1002/2211-5463.12614	brain tissue, neural stem cells	animal, in vitro	45	collected 28 days post-onset	*pathobiological study/intervention	anti-neurogenesis	Therapy
**27b-3p**	https://doi.org/10.1042/BSR20181033	serum	human	77 (42 control)	collected within 30 hours post-onset	increased	negative correalation with IGF-1/IGFBP3, brain development and growth	Diagnostic Biomarker
	https://doi.org/10.3389/fgene.2019.00814	brain tissue (Day 1 data)	animal	30	collected days 1/3/7/14/28 post-onset	increased (0.446709443 fold)	regulate synapses, cognition, axonogenesis, and ion transmembrane transport	Diagnostic Biomarker
		brain tissue (Day 14 data)	animal	30	collected days 1/3/7/14/28 post-onset	increased (0.510030457 fold)	regulate synapses, cognition, axonogenesis, and ion transmembrane transport	Diagnostic Biomarker
**19b**	https://doi.org/10.1371/journal.pone.0083717	brain tissue, neuroblastoma cells	animal, in vitro	N/A	collected 8 hours post-OGD termination	increased	apoptosis	Therapy

**339-5p**		brain tissue, neuroblastoma cells	animal, in vitro	N/A	collected 8 hours post-OGD termination	increased	apoptosis	Therapy
**16**	https://doi.org/10.1371/journal.pone.0166688	plasma	human	40 (30 control)	collected < 6 hours post-onset	increased	apoptosis	Diagnostic Biomarker/Prognosis
**32-5p**	https://doi.org/10.1007/s12035-018-1295-2	Plasma	Human	11	Within 24 hr post stroke	downregulated	NA	Therapy
**579-3p**	https://doi.org/10.1007/s12035-018-1295-2	Plasma	Human	11	Within 24 hr post stroke	downregulated	NA	Therapy
**4656**	https://doi.org/10.1007/s12035-016-0347-8	peripheral blood mononuclear cells	human	20 (19 control)	collected within 48 hours of onset	increased (+/- 1.5 fold)	inflammatory	Diagnostic Biomarker/Prognosis
**432**	https://doi.org/10.1007/s12035-016-0347-8	peripheral blood mononuclear cells	human	20 (19 control)	collected within 48 hours of onset	increased (+/- 1.5 fold)	inflammatory	Diagnostic Biomarker/Prognosis
**181**	https://doi.org/10.1016/j.expneurol.2014.11.007	brain tissue	animal	220	collected 24 or 48 hours post-onset	*pathobiological study/intervention	inflammatory	Therapy
**181a**	https://doi.org/10.1038/jcbfm.2013.157	brain tissue, N2a cells	animal, in vitro	97	collected 5 or 24 hours/1 and 3 days/7days post-onset	increased	neuronal loss, astrocyte dysfunction	Therapy
	https://doi.org/10.1016/j.mcn.2017.05.004	brain tissue, astrocytes	animal, in vitro	140	collected 24 hours post-onset	*pathobiological study/intervention	increase cell death	Therapy
	https://doi.org/10.1523/ENEURO.0002-19.2019	brain tissue, neural precursor cells	animal, in vitro	N/A	collected 7/14/21/28/70/91 days post-onset	*pathobiological study/intervention	anti-neurogenesis	Therapy
	https://doi.org/10.1016/j.nbd.2011.09.012	brain tissue, astrocytes	animal, in vitro	N/A	collected 24 hours post-onset	increased (core), decreased (penumbra)	apoptosis	Therapy
	https://doi.org/10.1007/s12035-020-02018-w	whole blood, plasma, SH-SY5Y cells	human, animal, in vitro	48 patients, 10 (10 control) piglets	collected within 3 hours post-delivery in patients / collected at 0/1/2/8/72 hours post-onset in animals	increased (resolves after 1 hour)	neuronal injury	Diagnostic Biomarker
	https://doi.org/10.3390/ijms23136885	brain tissue	animal	34	collected 48 hours post-onset	*pathobiological study/intervention	anti-autophagy	Therapy
**181c**	https://doi.org/10.1186/1742-2094-9-211	brain tissue, BV2 cells, microglia	animal, in vitro	N/A	collected 3 days post-onset	decreased	anti-apoptosis	Therapy
	https://doi.org/10.14336/AD.2016.0320	plasma, brain tissue, Neuro-2a cells, microglia, lymphocytes	human, animal, in vitro	10 (7 control) patients	collected within 72 hours of symptom onset in patients / collected 24 hours post-reperfusion in animals	decreased	apoptosis	Diagnostic Biomarker
**181d**	https://doi.org/10.3390/ijms161024302	brain tissue	animal	N/A	collected 6 hours post-onset	decreased (0.5 fold)	target genes involved in MAPK and Neurotrophin signaling pathways	Diagnostic Biomarkers/Therapy
**872**	https://doi.org/10.3390/ijms161024302	brain tissue	animal	N/A	collected 6 hours post-onset	decreased (0.5 fold)	target genes involved in MAPK and Neurotrophin signaling pathways	Diagnostic Biomarkers/Therapy
**344**		brain tissue	animal	N/A	collected 6 hours post-onset	increased (2 fold)	target genes involved in MAPK and PI3K-Akt signaling pathways	Diagnostic Biomarkers/Therapy
**669c-3p**	https://doi.org/10.1186/s12974-020-01870-w	brain tissue, microglia, astrocytes, N2a cell lines	animal, in vitro	69	collected 1 or 3 days post-onset	increased	anti-inflammatory, neuroprotective	Therapy
**129-2-3p**	https://doi.org/10.1111/jcmm.13901	whole blood, THP-1, U937, HEK293T, HUVEC cell lines	human, in vitro	270 (270 control)	collected 12 hours post-admission with symptoms occurring >24 hours	decreased	platelet activation	Therapy
	https://doi.org/10.3389/fgene.2019.00814	brain tissue (Day 7 data)	animal	30	collected days 1/3/7/14/28 post-onset	decreased (-0.808240308 fold)	regulate inflammation, immune responses, and angiogenesis	Diagnostic Biomarker
**652**	https://doi.org/10.1016/j.biopha.2020.109860	brain tissue, plasma, SH-SY5Y cells	animal, in vitro	32	collected 26 hours post-onset	decreased	anti-apoptosis	Therapy

**378**	https://doi.org/10.1074/jbc.RA119.010946	brain tissue	animal, in vitro	40	collected 3 days post-onset	decreased	inhibit neuronal death and autophagy	Therapy
	https://doi.org/10.3390/ijms17091427	brain tissue, N2A cells	animal, in vitro	N/A	collected at 24 or 72 hours post-reperfusion in animals / 0/6/12/24/48 hours post-onset in vitro	decreased	anti-apoptosis	Therapy
**1181**	https://doi.org/10.1186/s12920-019-0566-8	whole blood, mononuclear cells	human	107 patients and controls	collected within 3 hours post-onset	decreased	anoxia, inflammation, cell death	Diagnostic Biomarkers/Therapy
**1207-3p**		whole blood, mononuclear cells	human	107 patients and controls	collected within 3 hours post-onset	decreased	anoxia, inflammation, cell death	Diagnostic Biomarkers/Therapy
**1246**		whole blood, mononuclear cells	human	107 patients and controls	collected within 3 hours post-onset	increased	anoxia, inflammation, cell death	Diagnostic Biomarkers/Therapy
**3180**		whole blood, mononuclear cells	human	107 patients and controls	collected within 3 hours post-onset	decreased	anoxia, inflammation, cell death	Diagnostic Biomarkers/Therapy
**3180-3p**		whole blood, mononuclear cells	human	107 patients and controls	collected within 3 hours post-onset	decreased	anoxia, inflammation, cell death	Diagnostic Biomarkers/Therapy
**3960**		whole blood, mononuclear cells	human	107 patients and controls	collected within 3 hours post-onset	increased	anoxia, inflammation, cell death	Diagnostic Biomarkers/Therapy
**4436a**		whole blood, mononuclear cells	human	107 patients and controls	collected within 3 hours post-onset	decreased	anoxia, inflammation, cell death	Diagnostic Biomarkers/Therapy
**517a-3p**		whole blood, mononuclear cells	human	107 patients and controls	collected within 3 hours post-onset	decreased	anoxia, inflammation, cell death	Diagnostic Biomarkers/Therapy
**517b-3p**		whole blood, mononuclear cells	human	107 patients and controls	collected within 3 hours post-onset	decreased	anoxia, inflammation, cell death	Diagnostic Biomarkers/Therapy
**1229-3p**		whole blood, mononuclear cells	human	107 patients and controls	collected within 3 hours post-onset	decreased	anoxia, inflammation, cell death	Diagnostic Biomarkers/Therapy
**1262**		whole blood, mononuclear cells	human	107 patients and controls	collected within 3 hours post-onset	decreased	anoxia, inflammation, cell death	Diagnostic Biomarkers/Therapy
**138-2-3p**		whole blood, mononuclear cells	human	107 patients and controls	collected within 3 hours post-onset	decreased	anoxia, inflammation, cell death	Diagnostic Biomarkers/Therapy
**1909-5p**		whole blood, mononuclear cells	human	107 patients	collected within 3 hours post-onset	decreased	anoxia, inflammation, cell death	Diagnostic Biomarkers/Therapy

				and controls				
**199a-5p**	https://doi.org/10.1186/s12920-019-0566-8	whole blood, mononuclear cells	human	107 patients and controls	collected within 3 hours post-onset	increased	anoxia, inflammation, cell death	Diagnostic Biomarkers/Therapy
	https://pubmed.ncbi.nlm.nih.gov/33194051	brain tissue	animal	120	collected after 3 days post-onset	increased	apoptosis	Therapy
	https://doi.org/10.3389/fgene.2019.00814	brain tissue (Day 7 data)	animal	30	collected days 1/3/7/14/28 post-onset	increased (3.01612983 fold)	regulate synapses, cognition, axonogenesis, and ion transmembrane transport	Diagnostic Biomarker
		brain tissue (Day 14 data)	animal	30	collected days 1/3/7/14/28 post-onset	increased (2.490917784 fold)	regulate synapses, cognition, axonogenesis, and ion transmembrane transport	Diagnostic Biomarker
		brain tissue (Day 28 data)	animal	30	collected days 1/3/7/14/28 post-onset	increased (2.173281932 fold)	regulate synapses, cognition, axonogenesis, and ion transmembrane transport	Diagnostic Biomarker
**29c-5p**	https://doi.org/10.1186/s12920-019-0566-8	whole blood, mononuclear cells	human	107 patients and controls	collected within 3 hours post-onset	decreased	anoxia, inflammation, cell death	Diagnostic Biomarkers/Therapy
	https://doi.org/10.1515/med-2022-0438	blood, astrocytes, BMECs	human, in vitro	20 (20 control) patients	N/A	increased	inflammation	Therapy
	https://doi.org/10.3389/fgene.2019.00814	brain tissue (Day 3 data)	animal	30	collected days 1/3/7/14/28 post-onset	increased (1.196368062 fold)	regulate synapses, cognition, axonogenesis, and ion transmembrane transport	Diagnostic Biomarker
		brain tissue (Day 14 data)	animal	30	collected days 1/3/7/14/28 post-onset	decreased (-0.603095452 fold)	regulate inflammation, immune responses, and angiogenesis	Diagnostic Biomarker
**3129-5p**	https://doi.org/10.1186/s12920-019-0566-8	whole blood, mononuclear cells	human	107 patients and controls	collected within 3 hours post-onset	decreased	anoxia, inflammation, cell death	Diagnostic Biomarkers/Therapy
**3612**		whole blood, mononuclear cells	human	107 patients and controls	collected within 3 hours post-onset	decreased	anoxia, inflammation, cell death	Diagnostic Biomarkers/Therapy
**3620-3p**		whole blood, mononuclear cells	human	107 patients and controls	collected within 3 hours post-onset	decreased	anoxia, inflammation, cell death	Diagnostic Biomarkers/Therapy
**3657**		whole blood, mononuclear cells	human	107 patients and controls	collected within 3 hours post-onset	decreased	anoxia, inflammation, cell death	Diagnostic Biomarkers/Therapy
**371a-3p**		whole blood, mononuclear cells	human	107 patients and controls	collected within 3 hours post-onset	decreased	anoxia, inflammation, cell death	Diagnostic Biomarkers/Therapy
**4259**		whole blood, mononuclear cells	human	107 patients and controls	collected within 3 hours post-onset	decreased	anoxia, inflammation, cell death	Diagnostic Biomarkers/Therapy
**4725-5p**		whole blood, mononuclear cells	human	107 patients	collected within 3 hours post-onset	increased	anoxia, inflammation, cell death	Diagnostic Biomarkers/Therapy

				and controls				
**520a-3p**		whole blood, mononuclear cells	human	107 patients and controls	collected within 3 hours post-onset	decreased	anoxia, inflammation, cell death	Diagnostic Biomarkers/Therapy
**548n**		whole blood, mononuclear cells	human	107 patients and controls	collected within 3 hours post-onset	decreased	anoxia, inflammation, cell death	Diagnostic Biomarkers/Therapy
**551b-3p**	https://doi.org/10.1186/s12920-019-0566-8	whole blood, mononuclear cells	human	107 patients and controls	collected within 3 hours post-onset	decreased	anoxia, inflammation, cell death	Diagnostic Biomarkers/Therapy
	https://doi.org/10.3389/fgene.2019.00814	brain tissue (Day 3 data)	animal	30	collected days 1/3/7/14/28 post-onset	increased (1.643489723 fold)	regulate synapses, cognition, axonogenesis, and ion transmembrane transport	Diagnostic Biomarker
**5587-3p**	https://doi.org/10.1186/s12920-019-0566-8	whole blood, mononuclear cells	human	107 patients and controls	collected within 3 hours post-onset	decreased	anoxia, inflammation, cell death	Diagnostic Biomarkers/Therapy
**5588-5p**		whole blood, mononuclear cells	human	107 patients and controls	collected within 3 hours post-onset	decreased	anoxia, inflammation, cell death	Diagnostic Biomarkers/Therapy
**607**		whole blood, mononuclear cells	human	107 patients and controls	collected within 3 hours post-onset	decreased	anoxia, inflammation, cell death	Diagnostic Biomarkers/Therapy
**615-5p**		whole blood, mononuclear cells	human	107 patients and controls	collected within 3 hours post-onset	decreased	anoxia, inflammation, cell death	Diagnostic Biomarkers/Therapy
**150-5p**	10.26355/eurrev_202004_20854	plasma, brain tissue	human, animal	15 (15 control) patients, 60 rats	collected 3 days post-onset in animal model	increased	cell proliferation, apoptosis	Therapy
	https://doi.org/10.5853/jos.2017.00423	plasma	human	329	collected within 72 hours of symptom onset	*pathobiological study/intervention	neuroprotection, regulate inflammation	Prognosis
	https://doi.org/10.3389/fgene.2019.00814	brain tissue (Day 7 data)	animal	30	collected days 1/3/7/14/28 post-onset	decreased (-0.851298636 fold)	regulate inflammation, immune responses, and angiogenesis	Diagnostic Biomarker
**494**	https://doi.org/10.1177/0271678X19875201	plasma, brain tissue	human, animal, in vitro	76 (52 control) patients, 234 mice	collected within 6 hours post-onset /collected 45 min/1day/3days post-reperfusion in animal model	increased	anti-apoptosis, promote axonal plasticity	Diagnostic Biomarkers/Therapy
	https://doi.org/10.1111/bph.14852	blood, lymphocytes, brain tissue	human, animal, in vitro	50 (45 control) patients	collected within 6 hours post-onset in patients / collected 72 hours post-onset in mice	increased	apoptosis, inflammation	Diagnostic Biomarker/Prognosis/Therapy
**103**	https://doi.org/10.22038/ijbms.2018.27267.6657	brain tissue, HUVECs	animal, in vitro	40	N/A	decreased	anti-angiogenesis	Therapy
**103-1**	https://doi.org/10.1038/mt.2014.113	brain tissue, BHK cells, PC12 cells	animal, in vitro	103	collected 24 or 48 hours post-onset	increased	apoptosis	Diagnostic Biomarker/Therapy

**202-3p**	https://doi.org/10.18632/aging.202889	brain tissue, BMSCs, SH-SY5Y cells	animal, in vitro	40	N/A	*pathobiological study/intervention	anti-inflammatory, promotes angiogenesis	Therapy
**202-5p**	https://doi.org/10.1016/j.mcp.2019.101497	brain tissue, N2a cells	animal, in vitro	N/A	collected 3/7/11/15 hours post-onset in vitro	decreased	anti-apoptosis, increase proliferation, decrease autophagy	Therapy
**340-5p**	https://doi.org/10.3390/biom12010115	PC12 cells	in vitro	N/A	N/A	decreased	anti-inflammatory, anti-apoptosis	Therapy
	https://doi.org/10.3389/fgene.2019.00814	brain tissue (Day 3 data)	animal	30	collected days 1/3/7/14/28 post-onset	increased (1.304939009 fold)	regulate synapses, cognition, axonogenesis, and ion transmembrane transport	Diagnostic Biomarker
**99b**	https://doi.org/10.1042/BSR20201154	blood	human	24 (24 control)	N/A	increased	cell growth, differentiation, apoptosis, angiogenesis	Diagnostic Biomarker/Therapy
	https://doi.org/10.1002/jcla.23093	plasma	human	112 (122 control)	N/A	decreased	angiogenesis	Diagnostic Biomarker/Prognosis
**3130-1**	https://doi.org/10.1042/BSR20201154	blood	human	24 (24 control)	N/A	increased	cell growth, differentiation, apoptosis, angiogenesis	Diagnostic Biomarker/Therapy
**4532**	https://doi.org/10.1161/JAHA.116.005363	serum	human	60	collected at day 7 after Subarachnoid Hemorrhage	decreased	neurogenesis	Therapy
**4463**		serum	human	60	collected at day 7 after Subarachnoid Hemorrhage	decreased	neurogenesis	Therapy
**1290**		serum	human	60	collected at day 7 after Subarachnoid Hemorrhage	decreased	neurogenesis	Therapy
**4793**		serum	human	60	collected at day 7 after Subarachnoid Hemorrhage	decreased	neurogenesis	Therapy
**218-5p**	DOI: 10.12659/MSM.920101	PC12 cells	in vitro	N/A	N/A	increased	pro-inflammatory, pro-apoptosis, oxidative stress	Diagnostic Biomarker/Prognosis
**let-7e-5p**	https://doi.org/10.1371/journal.pone.0163951	whole blood, U937 cells	human	346 (346 control)	collected within 12 hours of hospital admission with stroke onset for > 24 hours	increased	apoptosis	Diagnostic Biomarker
**Let-7e-5p**	https://doi.org/10.1186%2F1471-2377-13-178	Serum	Human	197	Within 24 hr post stroke	Increased	NA	Diagnostic Biomarker
**Let-7e**	https://doi.org/10.1007/s12035-018-1295-2	Serum	Human	11	Within 24 hr post stroke	increased	NA	Diagnostic Biomarker
**383**	https://doi.org/10.1159/000447838	brain tissue, astrocytes	animal, in vitro	N/A	collected within 24 hours post-onset	decreased	inflammation	Therapy
**140-3p**	https://doi.org/10.1016/j.brainresbull.2022.11.007	N2a cells	in vitro	N/A	N/A	increased	apoptosis	Therapy
**1264-3p**	https://doi.org/10.1161/STROKEAHA.116.013942	blood, brain tissue	animal	125	collected 3 hours post-onset	increased	brain remodeling and neural regeneration	Diagnostic Biomarker/Prognosis
	https://doi.org/10.3389/fgene.2019.00814	brain tissue (Day 1 data)	animal	30	collected days 1/3/7/14/28 post-onset	increased (0.946819591 fold)	regulate synapses, cognition, axonogenesis, and ion transmembrane transport	Diagnostic Biomarker
**1298-5p**	https://doi.org/10.1161/STROKEAHA.116.013942	blood, brain tissue	animal	125	collected 3 hours post-onset	increased	brain remodeling and neural regeneration	Diagnostic Biomarker/Prognosis
**448-3p**		blood, brain tissue	animal	125	collected 3 hours post-onset	increased	brain remodeling and neural regeneration	Diagnostic Biomarker/Prognosis
**1296-5p**	https://doi.org/10.1016/j.ygeno.2020.03.011	peripheral blood	human	3 (3 control)	collected immediately post-admission	increased	autophagy	Diagnostic Biomarker
**1**	https://doi.org/10.1155/2021/9988534	brain tissue	animal	40	collected 3 days post-onset	increased	apoptosis	Therapy
**138**	https://doi.org/10.1038/pr.2014.104	brain tissue	animal	N/A	collected 24/48/72 hours and 7 days post-onset	increased	regulate oligodendroglial progenitor cells differentiation	Prognosis
**24**	10.5137/1019-5149.JTN.23127-18.2	brain tissue	animal	50	collected 24 hours post-onset	decreased	anti-apoptosis	Diagnostic Biomarker/Therapy
	PMID: 27508039	brain tissue	animal, in vitro	N/A	collected 25 hours post-onset	*pathobiological study/intervention	apoptosis, decrease cell viability	Therapy
**375**	https://doi.org/10.1042/BSR20171242	brain tissue, PC12 cells	animal, in vitro	25	collected 24 hours post-reperfusion	decreased	anti-apoptosis	Therapy

**1906**	https://doi.org/10.1523/JNEUROSCI.1139-17.2017	brain tissue, astrocytes, microglia	animal, in vitro	N/A	collected 2/4/6/12/24 post-onset	increased	anti-inflammatory	Therapy
**324-3p**	DOI: 10.4103/1673-5374.339009	plasma, brain tissue, PC12 cells	human, animal, in vitro	18 (13 control) patients, 74 rats	collected 11.0 ± 9.1 hours post-onset in patients /collected 24 hours post-onset in animals	decreased	apoptosis	Therapy
**324-5p**	https://doi.org/10.3892/etm.2019.8249	blood, brain tissue	human, animal, in vitro	80 (80 control) patients	collected 12/24/48 hours post-onset in vitro	decreased	apoptosis	Diagnostic Biomarker/Prognosis
	https://doi.org/10.3389/fgene.2019.00814	brain tissue (Day 1 data)	animal	30	collected days 1/3/7/14/28 post-onset	decreased (-1.110723142 fold)	regulate inflammation, immune responses, and angiogenesis	Diagnostic Biomarker
		brain tissue (Day 14 data)	animal	30	collected days 1/3/7/14/28 post-onset	decreased (-0.639471929 fold)	regulate inflammation, immune responses, and angiogenesis	Diagnostic Biomarker
		brain tissue (Day 28 data)	animal	30	collected days 1/3/7/14/28 post-onset	decreased (-1.321519518 fold)	regulate inflammation, immune responses, and angiogenesis	Diagnostic Biomarker
**15a**	PMID: 26885038	serum	human	106 (120 control)	N/A	increased (8.3 fold)	apoptosis, anti-angiogenesis, disrupt blood brain barrier	Diagnostic Biomarker
**16**	PMID: 26885038	serum	human	106 (120 control)	N/A	increased (42 fold)	apoptosis, anti-angiogenesis	Diagnostic Biomarker
**17-5p**	PMID: 26885038	serum	human	106 (120 control)	N/A	increased (9.9 fold)	neurogenesis	Diagnostic Biomarker
	https://doi.org/10.1016/j.neulet.2022.136475	brain tissue	animal	N/A	collected 28 hours post-onset	decreased	neuroprotection, anti-apoptosis	Therapy
	https://doi.org/10.3892/mmr.2020.11143	peripheral blood mononuclear cells, peripheral blood (female data)	human	260 (160 control)	N/A	*pathobiological study/intervention	inflammation, apoptosis	Diagnostic Biomarker/Therapy
		peripheral blood mononuclear cells, peripheral blood (male data)	human	260 (160 control)	N/A	*pathobiological study/intervention	inflammation, apoptosis	Diagnostic Biomarker/Therapy
	https://doi.org/10.3389/fgene.2019.00814	brain tissue (Day 7 data)	animal	30	collected days 1/3/7/14/28 post-onset	increased (1.107733915 fold)	regulate synapses, cognition, axonogenesis, and ion transmembrane transport	Diagnostic Biomarker
		brain tissue (Day 14 data)	animal	30	collected days 1/3/7/14/28 post-onset	increased (1.103012744 fold)	regulate synapses, cognition, axonogenesis, and ion transmembrane transport	Diagnostic Biomarker
**151a**	https://doi.org/10.1007/s12035-020-02018-w	whole blood, plasma, SH-SY5Y cells	human, animal, in vitro	48 patients, 10 (10 control) piglets	collected within 3 hours post-delivery in patients / collected at 0/1/2/8/72 hours post-onset in animals	increased (resolves after 1 hour)	neuronal injury	Diagnostic Biomarker
**421**	https://doi.org/10.3389/fnins.2021.738576	plasma	animal	3	collected 3 hours post-onset	increased (0.69 fold)	regulated proteolysis and chemokine signaling	Diagnostic Biomarker
**409-5p**		plasma	animal	3	collected 3 hours post-onset	increased (1.10 fold)	regulated proteolysis and chemokine signaling	Diagnostic Biomarker
**128a-5p_R − 2**		plasma	animal	3	collected 3 hours post-onset	decreased (1.03 fold)	regulated proteolysis and chemokine signaling	Diagnostic Biomarker
**431_R − 1**		plasma	animal	3	collected 3 hours post-onset	decreased (0.92 fold)	regulated proteolysis and chemokine signaling	Diagnostic Biomarker
**let-7g-3p_1ss22CT**		plasma	animal	3	collected 3 hours post-onset	decreased (3.07 fold)	regulated proteolysis and chemokine signaling	Diagnostic Biomarker/Therapy

**1197**	plasma	animal	3	collected 3 hours post-onset	decreased (1.54 fold)	regulated proteolysis and chemokine signaling	Diagnostic Biomarker
**299-3p**	plasma	animal	3	collected 3 hours post-onset	decreased (2.32 fold)	regulated proteolysis and chemokine signaling	Diagnostic Biomarker
**7174-5p**	plasma	animal	3	collected 3 hours post-onset	decreased (2.81 fold)	regulated proteolysis and chemokine signaling	Diagnostic Biomarker
**423-3p**	plasma	animal	3	collected 3 hours post-onset	decreased (1.63 fold)	regulated proteolysis and chemokine signaling	Diagnostic Biomarker
**PC-5p-15652_85**	plasma	animal	3	collected 3 hours post-onset	decreased (3.38 fold)	regulated proteolysis and chemokine signaling	Diagnostic Biomarker
**154-5p**	plasma	animal	3	collected 3 hours post-onset	decreased (1.60 fold)	regulated proteolysis and chemokine signaling	Diagnostic Biomarker
**380-5p**	plasma	animal	3	collected 3 hours post-onset	decreased (1.81 fold)	regulated proteolysis and chemokine signaling	Diagnostic Biomarker
**361-3p**	plasma	animal	3	collected 3 hours post-onset	decreased (0.73 fold)	regulated proteolysis and chemokine signaling	Diagnostic Biomarker
**154-5p_R+1**	plasma	animal	3	collected 3 hours post-onset	decreased (2.34 fold)	regulated proteolysis and chemokine signaling	Diagnostic Biomarker
**665_R-2**	plasma	animal	3	collected 3 hours post-onset	decreased (0.65 fold)	regulated proteolysis and chemokine signaling	Diagnostic Biomarker
**185-3p**	plasma	animal	3	collected 3 hours post-onset	decreased (2.56 fold)	regulated proteolysis and chemokine signaling	Diagnostic Biomarker
**1285-p3_1ss16CT**	plasma	animal	3	collected 3 hours post-onset	decreased (1.6 fold)	regulated proteolysis and chemokine signaling	Diagnostic Biomarker
**410-3p**	plasma	animal	3	collected 3 hours post-onset	decreased (2.53 fold)	regulated proteolysis and chemokine signaling	Diagnostic Biomarker
**7172-5p_R+1**	plasma	animal	3	collected 3 hours post-onset	decreased (2.16 fold)	regulated proteolysis and chemokine signaling	Diagnostic Biomarker
**376b-3p**	plasma	animal	3	collected 3 hours post-onset	decreased (4.24 fold)	regulated proteolysis and chemokine signaling	Diagnostic Biomarker
**660-3p**	plasma	animal	3	collected 3 hours post-onset	decreased (3.69 fold)	regulated proteolysis and chemokine signaling	Diagnostic Biomarker
**136-3p**	plasma	animal	3	collected 3 hours post-onset	decreased (1.29 fold)	regulated proteolysis and chemokine signaling	Diagnostic Biomarker
**107-3p_R-2**	plasma	animal	3	collected 3 hours post-onset	decreased (0.59 fold)	regulated proteolysis and chemokine signaling	Diagnostic Biomarker
**369-5p_R-1**	plasma	animal	3	collected 3 hours post-onset	decreased (0.94 fold)	regulated proteolysis and chemokine signaling	Diagnostic Biomarker
**127-5p_L-1**	plasma	animal	3	collected 3 hours post-onset	decreased (3.40 fold)	regulated proteolysis and chemokine signaling	Diagnostic Biomarker
**369-3p**	plasma	animal	3	collected 3 hours post-onset	decreased (1.05 fold)	regulated proteolysis and chemokine signaling	Diagnostic Biomarker
**339a_R+1_1ss22CT**	plasma	animal	3	collected 3 hours post-onset	decreased (2.45 fold)	regulated proteolysis and chemokine signaling	Diagnostic Biomarker
**655-3p**	plasma	animal	3	collected 3 hours post-onset	decreased (1.27 fold)	regulated proteolysis and chemokine signaling	Diagnostic Biomarker
**1468_R+1**	plasma	animal	3	collected 3 hours post-onset	decreased (2.70 fold)	regulated proteolysis and chemokine signaling	Diagnostic Biomarker
**1185-3p_L+2R+1**	plasma	animal	3	collected 3 hours post-onset	decreased (2.18 fold)	regulated proteolysis and chemokine signaling	Diagnostic Biomarker
**432-5p_1ss23GT**	plasma	animal	3	collected 3 hours post-onset	decreased (0.75 fold)	regulated proteolysis and chemokine signaling	Diagnostic Biomarker
**181c-5p_R+1**	plasma	animal	3	collected 3 hours post-onset	decreased (1.42 fold)	regulated proteolysis and chemokine signaling	Diagnostic Biomarker
**539_1ss21CT**	plasma	animal	3	collected 3 hours post-onset	decreased (1.42 fold)	regulated proteolysis and chemokine signaling	Diagnostic Biomarker

**382-3p_R-1**	plasma	animal	3	collected 3 hours post-onset	decreased (1.00 fold)	regulated proteolysis and chemokine signaling	Diagnostic Biomarker
**let-7j_R-2**	plasma	animal	3	collected 3 hours post-onset	decreased (2.66 fold)	regulated proteolysis and chemokine signaling	Diagnostic Biomarker
**204-3p_L-1**	plasma	animal	3	collected 3 hours post-onset	decreased (1.25 fold)	regulated proteolysis and chemokine signaling	Diagnostic Biomarker
**548x-3p_R+1**	plasma	animal	3	collected 3 hours post-onset	decreased (2.40 fold)	regulated proteolysis and chemokine signaling	Diagnostic Biomarker
**301a-5p_L+2**	plasma	animal	3	collected 3 hours post-onset	decreased (1.58 fold)	regulated proteolysis and chemokine signaling	Diagnostic Biomarker
**1296-5p_R-3**	plasma	animal	3	collected 3 hours post-onset	decreased (0.9 fold)	regulated proteolysis and chemokine signaling	Diagnostic Biomarker
**PC-5p-10049_140**	plasma	animal	3	collected 3 hours post-onset	decreased (3.19 fold)	regulated proteolysis and chemokine signaling	Diagnostic Biomarker
**7180-5p**	plasma	animal	3	collected 3 hours post-onset	decreased (0.87 fold)	regulated proteolysis and chemokine signaling	Diagnostic Biomarker
**1185-5p**	plasma	animal	3	collected 3 hours post-onset	decreased (0.98 fold)	regulated proteolysis and chemokine signaling	Diagnostic Biomarker
**28-3p**	plasma	animal	3	collected 3 hours post-onset	decreased (0.71 fold)	regulated proteolysis and chemokine signaling	Diagnostic Biomarker
**363-3p_R-1**	plasma	animal	3	collected 3 hours post-onset	decreased (0.46 fold)	regulated proteolysis and chemokine signaling	Diagnostic Biomarker
**PC-5p-16269_82**	plasma	animal	3	collected 3 hours post-onset	decreased (3.10 fold)	regulated proteolysis and chemokine signaling	Diagnostic Biomarker
**541-3p**	plasma	animal	3	collected 3 hours post-onset	decreased (2.17 fold)	regulated proteolysis and chemokine signaling	Diagnostic Biomarker
**323a-5p_R-1**	plasma	animal	3	collected 3 hours post-onset	decreased (0.78 fold)	regulated proteolysis and chemokine signaling	Diagnostic Biomarker
**33a_R+1**	plasma	animal	3	collected 3 hours post-onset	decreased (3.09 fold)	regulated proteolysis and chemokine signaling	Diagnostic Biomarker
**136_R-1**	plasma	animal	3	collected 3 hours post-onset	decreased (1.14 fold)	regulated proteolysis and chemokine signaling	Diagnostic Biomarker
**431_R-1**	plasma	animal	3	collected 3 hours post-onset	decreased (0.92 fold)	regulated proteolysis and chemokine signaling	Diagnostic Biomarker
**190b_R+1**	plasma	animal	3	collected 3 hours post-onset	decreased (0.75 fold)	regulated proteolysis and chemokine signaling	Diagnostic Biomarker
**PC-3p-7925_187**	plasma	animal	3	collected 3 hours post-onset	decreased (0.97 fold)	regulated proteolysis and chemokine signaling	Diagnostic Biomarker
**370-3p**	plasma	animal	3	collected 3 hours post-onset	decreased (2.31 fold)	regulated proteolysis and chemokine signaling	Diagnostic Biomarker
**660-5p_R+1**	plasma	animal	3	collected 3 hours post-onset	decreased (1.02 fold)	regulated proteolysis and chemokine signaling	Diagnostic Biomarker
**2478_L-1_1ss2TA**	plasma	animal	3	collected 3 hours post-onset	increased (1.72 fold)	regulated proteolysis and chemokine signaling	Diagnostic Biomarker
**1304**	plasma	animal	3	collected 3 hours post-onset	increased (1.12 fold)	regulated proteolysis and chemokine signaling	Diagnostic Biomarker
**6236_p5_1ss5CG**	plasma	animal	3	collected 3 hours post-onset	increased (4.14 fold)	regulated proteolysis and chemokine signaling	Diagnostic Biomarker
**6236_p3_1ss5CG**	plasma	animal	3	collected 3 hours post-onset	increased (4.14 fold)	regulated proteolysis and chemokine signaling	Diagnostic Biomarker
**1271-5p**	plasma	animal	3	collected 3 hours post-onset	increased (1.68 fold)	regulated proteolysis and chemokine signaling	Diagnostic Biomarker
**PC-3p-4437_437**	plasma	animal	3	collected 3 hours post-onset	increased (0.99 fold)	regulated proteolysis and chemokine signaling	Diagnostic Biomarker
**PC-5p-4437_437**	plasma	animal	3	collected 3 hours post-onset	increased (0.99 fold)	regulated proteolysis and chemokine signaling	Diagnostic Biomarker

**PC-3p-60777_13**		plasma	animal	3	collected 3 hours post-onset	increased (3.61 fold)	regulated proteolysis and chemokine signaling	Diagnostic Biomarker
**942-5p_L-1R+3**		plasma	animal	3	collected 3 hours post-onset	increased (0.88 fold)	regulated proteolysis and chemokine signaling	Diagnostic Biomarker
**361-5p**		plasma	animal	3	collected 3 hours post-onset	increased (0.64 fold)	regulated proteolysis and chemokine signaling	Diagnostic Biomarker
**377-5p**		plasma	animal	3	collected 3 hours post-onset	increased (0.91 fold)	regulated proteolysis and chemokine signaling	Diagnostic Biomarker
**93-3p_R+1**		plasma	animal	3	collected 3 hours post-onset	increased (1.13 fold)	regulated proteolysis and chemokine signaling	Diagnostic Biomarker
**548f-p5_1ss12CT**		plasma	animal	3	collected 3 hours post-onset	increased (1.83 fold)	regulated proteolysis and chemokine signaling	Diagnostic Biomarker
**654-3p_R-2**		plasma	animal	3	collected 3 hours post-onset	increased (0.96 fold)	regulated proteolysis and chemokine signaling	Diagnostic Biomarker
**374a-5p_R-1**		plasma	animal	3	collected 3 hours post-onset	increased (1.11 fold)	regulated proteolysis and chemokine signaling	Diagnostic Biomarker
**20a-5p_R+1**		plasma	animal	3	collected 3 hours post-onset	increased (0.52 fold)	regulated proteolysis and chemokine signaling	Diagnostic Biomarker
**15b-5p**		plasma	animal	3	collected 3 hours post-onset	increased (0.71 fold)	regulated proteolysis and chemokine signaling	Diagnostic Biomarker
**7184-3p**		plasma	animal	3	collected 3 hours post-onset	increased (1.90 fold)	regulated proteolysis and chemokine signaling	Diagnostic Biomarker
**4454_L+1_1ss3GA**		plasma	animal	3	collected 3 hours post-onset	increased (2.15 fold)	regulated proteolysis and chemokine signaling	Diagnostic Biomarker
**PC-5p-8713_165**		plasma	animal	3	collected 3 hours post-onset	increased (3.05 fold)	regulated proteolysis and chemokine signaling	Diagnostic Biomarker
**PC-5p-17128_77**		plasma	animal	3	collected 3 hours post-onset	increased (1.78 fold)	regulated proteolysis and chemokine signaling	Diagnostic Biomarker
**1306-5p**		plasma	animal	3	collected 3 hours post-onset	increased (1.29 fold)	regulated proteolysis and chemokine signaling	Diagnostic Biomarker
**409-5p**		plasma	animal	3	collected 3 hours post-onset	increased (1.10 fold)	regulated proteolysis and chemokine signaling	Diagnostic Biomarker
**4454_L-2**		plasma	animal	3	collected 3 hours post-onset	increased (1.54 fold)	regulated proteolysis and chemokine signaling	Diagnostic Biomarker
**7977_1ss6AG**		plasma	animal	3	collected 3 hours post-onset	increased (1.72 fold)	regulated proteolysis and chemokine signaling	Diagnostic Biomarker
**PC-3p-37822_29**		plasma	animal	3	collected 3 hours post-onset	increased (1.77 fold)	regulated proteolysis and chemokine signaling	Diagnostic Biomarker
**142-5p_L+2R-2**		plasma	animal	3	collected 3 hours post-onset	increased (1.39 fold)	regulated proteolysis and chemokine signaling	Diagnostic Biomarker
**PC-3p-11325_121**		plasma	animal	3	collected 3 hours post-onset	increased (1.12 fold)	regulated proteolysis and chemokine signaling	Diagnostic Biomarker
**PC-3p-21542_60**		plasma	animal	3	collected 3 hours post-onset	increased (1.31 fold)	regulated proteolysis and chemokine signaling	Diagnostic Biomarker
**146b-5p_R+2**		plasma	animal	3	collected 3 hours post-onset	increased (0.94 fold)	regulated proteolysis and chemokine signaling	Diagnostic Biomarker
**518-5p**	https://doi.org/10.3389/fneur.2022.852013	whole blood, monocytes, brain tissue	human, animal	63 (70 control) patients	N/A	decreased	vascular endothelial dysfunction, inflammation	Diagnostic Biomarker
**3135-b**		whole blood, monocytes, brain tissue	human, animal	63 (70 control) patients	N/A	decreased	vascular endothelial dysfunction, inflammation	Diagnostic Biomarker
**9-3p**	https://doi.org/10.2147/NDT.S290237	brain tissue, HT22 cell	animal, in vitro	12	collected 24 hours post-reperfusion	increased	oxidative stress	Therapy
	https://doi.org/10.2147/IJGM.S327594	whole blood, plasma, mononuclear cells	human	118 (74 control)	N/A	increased	inflammation and immune response	Diagnostic Biomarker/Therapy

	https://doi.org/10.3389/fgene.2019.00814	brain tissue (Day 3 data)	animal	30	collected days 1/3/7/14/28 post-onset	increased (2.02588253 fold)	regulate synapses, cognition, axonogenesis, and ion transmembrane transport	Diagnostic Biomarker
**488-3p**	https://doi.org/10.2147/NDT.S255666	brain tissue	animal, in vitro	N/A	collected 24 hours post-onset	decreased	anti-neuronal cell death	Therapy
	https://doi.org/10.3389/fgene.2019.00814	brain tissue (Day 3 data)	animal	30	collected days 1/3/7/14/28 post-onset	increased (1.482032791 fold)	regulate synapses, cognition, axonogenesis, and ion transmembrane transport	Diagnostic Biomarker
		brain tissue (Day 28 data)	animal	30	collected days 1/3/7/14/28 post-onset	decreased (-0.643956758 fold)	regulate inflammation, immune responses, and angiogenesis	Diagnostic Biomarker
**exosomal 328-3p**	https://doi.org/10.2147/IJGM.S307392	serum, brain tissue	human, animal	39 (20 control) patients	collected 2 and 10 days post-onset	decreased	increase neutrophil infiltration, inflammation	Prognosis
**455**	https://doi.org/10.2147/NDT.S121183	brain tissue	animal, in vitro	N/A	collected 24 hours post-reperfusion	decreased	inhibit neuronal death	Therapy
**455-5p**	DOI: 10.4103/1673-5374.332154	brain tissue, peripheral blood, microglia	animal, in vitro	261	collected 24 hours post-onset	decreased	anti-inflammatory	Diagnostic Biomarker/Therapy
	https://doi.org/10.3389/fgene.2019.00814	brain tissue (Day 7 data)	animal	30	collected days 1/3/7/14/28 post-onset	increased (1.24462378 fold)	regulate synapses, cognition, axonogenesis, and ion transmembrane transport	Diagnostic Biomarker
**491-5p**	https://doi.org/10.3390/brainsci12080999	serum	human	215	collected within 24 hours of hospital admission	*pathobiological study/intervention	anti-inflammatory, anti-apoptosis	Prognosis
**206**	https://doi.org/10.3390/brainsci12080999	serum	human	215	collected within 24 hours of hospital admission	*pathobiological study/intervention	inhibit cell viability, proliferation, angiogenesis	Prognosis
**exosomal 206**	https://doi.org/10.1016/j.jare.2020.05.017	brain tissue, hNSCs	animal, in vitro	38	collected 28 days post-onset	*pathobiological study/intervention	cell survival, anti-oxidant	Therapy
**exosomal 412-5p**	https://doi.org/10.1021/acsomega.1c03248	bEnd.3 cells	in vitro	N/A	N/A	decreased (12.5054 fold)	angiogenesis, cell proliferation, and inflammatory regulation	Therapy
**exosomal 122-5p**		bEnd.3 cells	in vitro	N/A	N/A	decreased (9.1688 fold)	angiogenesis, cell proliferation, and inflammatory regulation	Therapy
**exosomal 369-3p**		bEnd.3 cells	in vitro	N/A	N/A	decreased (10.1621 fold)	angiogenesis, cell proliferation, and inflammatory regulation	Therapy
**exosomal 127-3p**		bEnd.3 cells	in vitro	N/A	N/A	decreased (7.5257 fold)	angiogenesis, cell proliferation, and inflammatory regulation	Therapy
**exosomal 409-3p**		bEnd.3 cells	in vitro	N/A	N/A	decreased (8.4086 fold)	angiogenesis, cell proliferation, and inflammatory regulation	Therapy
**exosomal 379-5p**		bEnd.3 cells	in vitro	N/A	N/A	decreased (9.9317 fold)	angiogenesis, cell proliferation, and inflammatory regulation	Therapy
**exosomal 494-3p**		bEnd.3 cells	in vitro	N/A	N/A	decreased (9.4927 fold)	angiogenesis, cell proliferation, and inflammatory regulation	Therapy
**exosomal 214-3p**		bEnd.3 cells	in vitro	N/A	N/A	decreased (14.8316 fold)	angiogenesis, cell proliferation, and inflammatory regulation	Therapy
**exosomal 299b-3p**		bEnd.3 cells	in vitro	N/A	N/A	decreased (14.7051 fold)	angiogenesis, cell proliferation, and inflammatory regulation	Therapy

**exosomal 8095**		bEnd.3 cells	in vitro	N/A	N/A	decreased (14.8813 fold)	angiogenesis, cell proliferation, and inflammatory regulation	Therapy
**exosomal 15b-5p**	https://doi.org/10.1155/2022/9264555	serum, brain tissue, mononuclear cells	human	242 (1664 control)	N/A	increased	inflammation, regulate macrophage or mast activation	Diagnostic Biomarker/Therapy
**184**	https://doi.org/10.3389/fnmol.2021.613887	brain tissue, SH-SY5Y cells	animal, in vitro	57	collected at 24 hours post-onset of reperfusion	decreased	anti-apoptosis, increased cell viability	Therapy
**exosomal 184**	https://doi.org/10.1155/2022/9264555	serum, brain tissue, mononuclear cells	human	242 (1664 control)	N/A	increased	inflammation, regulate macrophage or mast activation	Diagnostic Biomarker/Therapy
**exosomal 16-5p**	https://doi.org/10.1155/2022/9264555	serum, brain tissue, mononuclear cells	human	242 (1664 control)	N/A	increased	inflammation, regulate macrophage or mast activation	Diagnostic Biomarker/Therapy
**651-5p**	https://doi.org/10.2147/IJGM.S327594	whole blood, plasma, mononuclear cells	human	118 (74 control)	N/A	decreased	inflammation and immune response	Diagnostic Biomarker/Therapy
**7-5p**	https://doi.org/10.2147/IJGM.S327594	whole blood, plasma, mononuclear cells	human	118 (74 control)	N/A	decreased	inflammation and immune response	Diagnostic Biomarker/Therapy
**374a**	https://doi.org/10.1002/brb3.835	serum	human	167 (82 control)	collected within 1-48 hours post-delivery	decreased	regulate inflammation	Diagnostic Biomarker/Prognosis
	https://doi.org/10.1007/s12035-020-02018-w	whole blood, plasma, SH-SY5Y cells	human, animal, in vitro	48 patients, 10 (10 control) piglets	collected within 3 hours post-delivery in patients / collected at 0/1/2/8/72 hours post-onset in animals	increased (resolves after 1 hour)	neuronal injury	Diagnostic Biomarker
**374a-3p**	https://doi.org/10.2147/IJGM.S327594	whole blood, plasma, mononuclear cells	human	118 (74 control)	N/A	decreased	inflammation and immune response	Diagnostic Biomarker/Therapy
**374a-5p**	doi:10.1001/jamaneurol.2018.4182	umbilical cord blood	human	177 (44 control)	collected immediately following delivery of the placenta	decreased	regulate pleuropotency of stem cells	Diagnostic Biomarker
**223-5p**	https://doi.org/10.1016/j.omtn.2019.10.022	brain tissue	animal, in vitro	72	collected at 6/24/48/72 hours of reperfusion	increased	modulating NCKX2 expression, anti-neuroprotection	Therapy
	https://doi.org/10.3389/fgene.2019.00814	brain tissue (Day 1 data)	animal	30	collected days 1/3/7/14/28 post-onset	increased (4.943810993 fold)	regulate synapses, cognition, axonogenesis, and ion transmembrane transport	Diagnostic Biomarker
		brain tissue (Day 3 data)	animal	30	collected days 1/3/7/14/28 post-onset	increased (3.474816393 fold)	regulate synapses, cognition, axonogenesis, and ion transmembrane transport	Diagnostic Biomarker
		brain tissue (Day 7 data)	animal	30	collected days 1/3/7/14/28 post-onset	increased (3.656399062 fold)	regulate synapses, cognition, axonogenesis, and ion transmembrane transport	Diagnostic Biomarker
**exosomal 133a-3p**	https://doi.org/10.1016/j.jare.2020.05.017	brain tissue, hNSCs	animal, in vitro	38	collected 28 days post-onset	*pathobiological study/intervention	cell survival, anti-oxidant	Therapy
**exosomal 3656**		brain tissue, hNSCs	animal, in vitro	38	collected 28 days post-onset	*pathobiological study/intervention	cell survival, anti-oxidant	Therapy
**exosomal 200c-3p**	https://doi.org/10.1186/s13287-021-02668-0	brain tissue, ADSCs, microglia	animal, in vitro	28	collected 7 and 14 days post-onset	*pathobiological study/intervention	promoted M2 polarization of microglia, anti-inflammatory	Therapy
**93-3p**	https://doi.org/10.3389/fnint.2021.638114	plasma	human	34 (34 control)	N/A	increased	regulate pathogenesis	Diagnostic Biomarker/Therapy
**exosomal 93-3p**	https://doi.org/10.1186/s13287-021-02668-0	brain tissue, ADSCs, microglia	animal, in vitro	28	collected 7 and 14 days post-onset	*pathobiological study/intervention	promoted M2 polarization of microglia, anti-inflammatory	Therapy
**200a-3p**	https://doi.org/10.3389/fphys.2022.893102	serum microvesicles, platelets, HUVECs	human, in vitro	53(53 control) patients	N/A	increased	modulate apoptosis, reactive oxygen species	Diagnostic Biomarker/Therapy

	https://doi.org/10.3389/fgene.2019.00814	brain tissue (Day 3 data)	animal	30	collected days 1/3/7/14/28 post-onset	increased (3.121568121 fold)	regulate synapses, cognition, axonogenesis, and ion transmembrane transport	Diagnostic Biomarker
		brain tissue (Day 7 data)	animal	30	collected days 1/3/7/14/28 post-onset	increased (2.794825391 fold)	regulate synapses, cognition, axonogenesis, and ion transmembrane transport	Diagnostic Biomarker
**exosomal 200a-3p**	https://doi.org/10.1186/s13287-021-02668-0	brain tissue, ADSCs, microglia	animal, in vitro	28	collected 7 and 14 days post-onset	*pathobiological study/intervention	promoted M2 polarization of microglia, anti-inflammatory	Therapy
**exosomal 376a-3p**	https://doi.org/10.1186/s13287-021-02668-0	brain tissue, ADSCs, microglia	animal, in vitro	28	collected 7 and 14 days post-onset	*pathobiological study/intervention	promoted M2 polarization of microglia, anti-inflammatory	Therapy
**exosomal 144-3p**	https://doi.org/10.1186/s13287-021-02668-0	brain tissue, ADSCs, microglia	animal, in vitro	28	collected 7 and 14 days post-onset	*pathobiological study/intervention	promoted M2 polarization of microglia, anti-inflammatory	Therapy
**exosomal 345-5p**	https://doi.org/10.1186/s13287-021-02668-0	brain tissue, ADSCs, microglia	animal, in vitro	28	collected 7 and 14 days post-onset	*pathobiological study/intervention	promoted M2 polarization of microglia, anti-inflammatory	Therapy
**exosomal 429-3p**	https://doi.org/10.1186/s13287-021-02668-0	brain tissue, ADSCs, microglia	animal, in vitro	28	collected 7 and 14 days post-onset	*pathobiological study/intervention	promoted M2 polarization of microglia, anti-inflammatory	Therapy
**101**	https://doi.org/10.2147/NDT.S292471	brain tissue	animal, in vitro	N/A	collected after 24 hours of reperfusion	decreased	anti-apoptosis, increase cell viability	Therapy
**221**	https://doi.org/10.3892/etm.2019.8263	serum, HUVECs	human, in vitro	20 (20 control) patients	collected within 72 hours post-onset	decreased	anti-apoptosis, increase cell viability, angiogenesis	Therapy
**exosomal 9-3p**	https://doi.org/10.3389/fnmol.2022.874903	serum, brain tissue	human, animal	40 (33 control) patients	collected within 18.5 hours post-admission in patients / collected 1 day post-onset in animals	increased	blood brain barrier disruption	Diagnostic Biomarker/Prognosis
**exosomal 143-3p**		serum, brain tissue	human, animal	40 (33 control) patients	collected within 18.5 hours post-admission in patients / collected 1 day post-onset in animals	increased	blood brain barrier disruption	Diagnostic Biomarker/Prognosis
**exosomal 93-5p**		serum, brain tissue	human, animal	40 (33 control) patients	collected within 18.5 hours post-admission in patients / collected 1 day post-onset in animals	increased	blood brain barrier disruption	Diagnostic Biomarker/Prognosis
**exosomal 9-5p**		serum, brain tissue	human, animal	40 (33 control) patients	collected within 18.5 hours post-admission in patients / collected 1 day post-onset in animals	increased	blood brain barrier disruption	Diagnostic Biomarker/Prognosis
**exosomal 323-3p**		serum, brain tissue	human, animal	40 (33 control) patients	collected within 18.5 hours post-admission in patients / collected 1 day post-onset in animals	increased	blood brain barrier disruption	Diagnostic Biomarker/Prognosis
**exosomal 219b-5p**		serum, brain tissue	human, animal	40 (33 control) patients	collected within 18.5 hours post-admission in patients / collected 1 day post-onset in animals	increased	blood brain barrier disruption	Diagnostic Biomarker/Prognosis
**exosomal 129b-3p**		serum, brain tissue	human, animal	40 (33 control) patients	collected within 18.5 hours post-admission in patients / collected 1 day post-onset in animals	increased	blood brain barrier disruption	Diagnostic Biomarker/Prognosis
**exosomal 129-5p**		serum, brain tissue	human, animal	40 (33 control) patients	collected within 18.5 hours post-admission in patients / collected 1 day post-onset in animals	increased	blood brain barrier disruption	Diagnostic Biomarker/Prognosis
**exosomal 433-3p**		serum, brain tissue	human, animal	40 (33 control) patients	collected within 18.5 hours post-admission in patients /	increased	blood brain barrier disruption	Diagnostic Biomarker/Prognosis

					collected 1 day post-onset in animals			
**125b-5p**	https://doi.org/10.3389/fgene.2022.833545	whole blood, HT22 cells	human, in vitro	59 (44 control) patients	N/A	decreased	cell viability, angiogenesis	Diagnostic Biomarker
**125b-5p**	https://doi.org/10.1161/circresaha.117.311572	Plasma	human	260	5 hr post stroke	Upregulated	NA	Diagnostic Biomarker
	https://doi.org/10.3389/fgene.2019.00814	brain tissue (Day 1 data)	animal	30	collected days 1/3/7/14/28 post-onset	decreased (-0.756347164 fold)	regulate inflammation, immune responses, and angiogenesis	Diagnostic Biomarker
		brain tissue (Day 7 data)	animal	30	collected days 1/3/7/14/28 post-onset	decreased (-1.111563286 fold)	regulate inflammation, immune responses, and angiogenesis	Diagnostic Biomarker
**185**	https://doi.org/10.2147/IJGM.S340586	serum	human	142 (50 control)	collected within 24 hours post-onset	increased	severe neurological impairment	Diagnostic Biomarkers/Prognosis
**326**	https://doi.org/10.1186/s13041-020-00579-4	serum, CSF, brain tissue, PC12 cells	human, animal, in vitro	10 (10 control) patients	collected 1 day post-birth in patients/ collected 24/48/72 hours post-onset in animals	decreased	apoptosis	Therapy
**126a-5p**	https://doi.org/10.2147/NDT.S293611	brain tissue, SH-SY5Y cells	animal, in vitro	24	collected 26 hours post-onset	decreased	anti-apoptosis, anti-oxidative stress, increase cell-viability	Therapy
	https://doi.org/10.3389/fgene.2019.00814	brain tissue (Day 3 data)	animal	30	collected days 1/3/7/14/28 post-onset	increased (1.296828152 fold)	regulate synapses, cognition, axonogenesis, and ion transmembrane transport	Diagnostic Biomarker
**525-5p**	https://pubmed.ncbi.nlm.nih.gov/26770408	brain tissue	animal, in vitro	N/A	collected 0/12-48 hours post-onset	decreased	regulate neural death, inflammation	Therapy
**499a**	https://doi.org/10.1016/j.ncrna.2021.09.002	astrocytes	in vitro	N/A	N/A	decreased	anti-inflammatory	Therapy
**373**	https://doi.org/10.1155/2021/5553486	serum	human	45 (15 control)	N/A	increased	regulate cell proliferation, migration, apoptosis, invasion, and repairing damaged DNA	Diagnostic Biomarker/Prognosis/Therapy
**384**	https://doi.org/10.1038/s41419-019-1631-0	brain tissue, HT22 cells	animal, in vitro	144	N/A	increased	apoptosis	Therapy
**127**	https://doi.org/10.1111/acel.13287	brain tissue, N2a cells	human, animal, in vitro	6 patients, 60 mice	collected 1 day post-onset in animals	decreased	apoptosis, inflammation	Therapy
**505-5p**	https://doi.org/10.3390/ijms21093107	venous blood	human	23 (22 control)	collected 1-12 hours post-onset	increased	regulate pathogenesis	Diagnostic Biomarker/Therapy
**1255b-5p**		venous blood	human	23 (22 control)	collected 1-12 hours post-onset	increased	regulate pathogenesis	Diagnostic Biomarker/Therapy
**550b-2-5p**		venous blood	human	23 (22 control)	collected 1-12 hours post-onset	increased	anti-apoptosis	Diagnostic Biomarker/Therapy
**4523**		venous blood	human	23 (22 control)	collected 1-12 hours post-onset	decreased	regulate pathogenesis	Diagnostic Biomarker/Therapy
**6795-3p**		venous blood	human	23 (22 control)	collected 1-12 hours post-onset	decreased	regulate pathogenesis	Diagnostic Biomarker/Therapy
**let-7f-5p**	https://doi.org/10.3892/mmr.2020.11143	peripheral blood mononuclear cells, peripheral blood (female data)	human	260 (160 control)	N/A	*pathobiological study/intervention	inflammation, apoptosis	Diagnostic Biomarker/Therapy
		peripheral blood mononuclear cells, peripheral blood (male data)	human	260 (160 control)	N/A	*pathobiological study/intervention	inflammation, apoptosis	Diagnostic Biomarker/Therapy

	https://doi.org/10.3389/fgene.2019.00814	brain tissue (Day 7 data)	animal	30	collected days 1/3/7/14/28 post-onset	increased (0.874499967 fold)	regulate synapses, cognition, axonogenesis, and ion transmembrane transport	Diagnostic Biomarker
**let-7i-5p**	https://doi.org/10.3892/mmr.2020.11143	peripheral blood mononuclear cells, peripheral blood (female data)	human	260 (160 control)	N/A	*pathobiological study/intervention	inflammation, apoptosis	Diagnostic Biomarker/Therapy
		peripheral blood mononuclear cells, peripheral blood (male data)	human	260 (160 control)	N/A	*pathobiological study/intervention	inflammation, apoptosis	Diagnostic Biomarker/Therapy
**20a-5p**	https://doi.org/10.3892/mmr.2020.11143	peripheral blood mononuclear cells, peripheral blood (female data)	human	260 (160 control)	N/A	*pathobiological study/intervention	inflammation, apoptosis	Diagnostic Biomarker/Therapy
		peripheral blood mononuclear cells, peripheral blood (male data)	human	260 (160 control)	N/A	*pathobiological study/intervention	inflammation, apoptosis	Diagnostic Biomarker/Therapy
	https://doi.org/10.3389/fgene.2019.00814	brain tissue (Day 3 data)	animal	30	collected days 1/3/7/14/28 post-onset	increased (1.719936513 fold)	regulate synapses, cognition, axonogenesis, and ion transmembrane transport	Diagnostic Biomarker
		brain tissue (Day 14 data)	animal	30	collected days 1/3/7/14/28 post-onset	increased (0.988200343 fold)	regulate synapses, cognition, axonogenesis, and ion transmembrane transport	Diagnostic Biomarker
**19b-3p**	https://doi.org/10.3892/mmr.2020.11143	peripheral blood mononuclear cells, peripheral blood (female data)	human	260 (160 control)	N/A	*pathobiological study/intervention	inflammation, apoptosis	Diagnostic Biomarker/Therapy
		peripheral blood mononuclear cells, peripheral blood (male data)	human	260 (160 control)	N/A	*pathobiological study/intervention	inflammation, apoptosis	Diagnostic Biomarker/Therapy
	https://doi.org/10.3389/fgene.2019.00814	brain tissue (Day 1 data)	animal	30	collected days 1/3/7/14/28 post-onset	increased (0.818978788 fold)	regulate synapses, cognition, axonogenesis, and ion transmembrane transport	Diagnostic Biomarker
		brain tissue (Day 3 data)	animal	30	collected days 1/3/7/14/28 post-onset	increased (2.811421124 fold)	regulate synapses, cognition, axonogenesis, and ion transmembrane transport	Diagnostic Biomarker
		brain tissue (Day 7 data)	animal	30	collected days 1/3/7/14/28 post-onset	increased (0.872014451 fold)	regulate synapses, cognition, axonogenesis, and ion transmembrane transport	Diagnostic Biomarker
**205-5p**	https://doi.org/10.3892/mmr.2020.11143	peripheral blood mononuclear cells, peripheral blood (female data)	human	260 (160 control)	N/A	*pathobiological study/intervention	inflammation, apoptosis	Diagnostic Biomarker/Therapy
		peripheral blood mononuclear cells, peripheral blood (male data)	human	260 (160 control)	N/A	*pathobiological study/intervention	inflammation, apoptosis	Diagnostic Biomarker/Therapy
**92a-3p**		peripheral blood mononuclear cells, peripheral blood (male data)	human	260 (160 control)	N/A	*pathobiological study/intervention	inflammation, apoptosis	Diagnostic Biomarker/Therapy
**18a-5p**	https://doi.org/10.3892/mmr.2020.11143	peripheral blood mononuclear cells,	human	260 (160 control)	N/A	*pathobiological study/intervention	inflammation, apoptosis	Diagnostic Biomarker/Therapy

		peripheral blood (male data)						
	https://doi.org/10.3389/fgene.2019.00814	brain tissue (Day 3 data)	animal	30	collected days 1/3/7/14/28 post-onset	increased (3.279925712 fold)	regulate synapses, cognition, axonogenesis, and ion transmembrane transport	Diagnostic Biomarker
		brain tissue (Day 14 data)	animal	30	collected days 1/3/7/14/28 post-onset	increased (2.765050612 fold)	regulate synapses, cognition, axonogenesis, and ion transmembrane transport	Diagnostic Biomarker
**185-5p**	https://doi.org/10.3892/mmr.2020.11143	peripheral blood mononuclear cells, peripheral blood (male data)	human	260 (160 control)	N/A	*pathobiological study/intervention	inflammation, apoptosis	Diagnostic Biomarker/Therapy
	https://doi.org/10.3389/fgene.2019.00814	brain tissue (Day 1 data)	animal	30	collected days 1/3/7/14/28 post-onset	decreased (-0.684189415 fold)	regulate inflammation, immune responses, and angiogenesis	Diagnostic Biomarker
**107-5p**	https://doi.org/10.3390/ijms18112335	brain tissue, whole blood	animal	14	collected 24 hours post-reperfusion	increased (1.85 fold)	regulate pathogenesis	Diagnostic Biomarker
	https://doi.org/10.3389/fgene.2019.00814	brain tissue (Day 3 data)	animal	30	collected days 1/3/7/14/28 post-onset	increased (2.062573576 fold)	regulate synapses, cognition, axonogenesis, and ion transmembrane transport	Diagnostic Biomarker
**383-5p**	https://doi.org/10.3390/ijms18112335	brain tissue, whole blood	animal	14	collected 24 hours post-reperfusion	increased (1.83 fold)	regulate pathogenesis	Diagnostic Biomarker
	https://doi.org/10.3389/fgene.2019.00814	brain tissue (Day 7 data)	animal	30	collected days 1/3/7/14/28 post-onset	decreased (-1.508318277 fold)	regulate inflammation, immune responses, and angiogenesis	Diagnostic Biomarker
**24-1-5p**	https://doi.org/10.3390/ijms18112335	brain tissue, whole blood	animal	14	collected 24 hours post-reperfusion	increased (1.59 fold)	regulate pathogenesis	Diagnostic Biomarker
**191b**		brain tissue, whole blood	animal	14	collected 24 hours post-reperfusion	increased (1.57 fold)	regulate pathogenesis	Diagnostic Biomarker
**196b-5p**		brain tissue, whole blood	animal	14	collected 24 hours post-reperfusion	increased (1.53 fold)	regulate immunodepression	Diagnostic Biomarker
**194-1**		brain tissue, whole blood	animal	14	collected 24 hours post-reperfusion	decreased (1.72 fold)	regulate pathogenesis	Diagnostic Biomarker
**3135b**	https://doi.org/10.3389/fnint.2021.638114	plasma	human	34 (34 control)	N/A	decreased	regulate vasculature development	Diagnostic Biomarker/Therapy
**24-3p**	https://doi.org/10.3389/fnint.2021.638114	plasma	human	34 (34 control)	N/A	increased	regulate pathogenesis	Diagnostic Biomarker/Therapy
	https://doi.org/10.3389/fgene.2019.00814	brain tissue (Day 1 data)	animal	30	collected days 1/3/7/14/28 post-onset	decreased (-0.631406401 fold)	regulate inflammation, immune responses, and angiogenesis	Diagnostic Biomarker
		brain tissue (Day 28 data)	animal	30	collected days 1/3/7/14/28 post-onset	decreased (-0.894036576 fold)	regulate inflammation, immune responses, and angiogenesis	Diagnostic Biomarker
**548ac**		plasma	human	34 (34 control)	N/A	increased	regulate apoptosis, inflammation	Diagnostic Biomarker/Therapy
**2137**	https://doi.org/10.3389/fgene.2019.00814	brain tissue (Day 1 data)	animal	30	collected days 1/3/7/14/28 post-onset	increased (9.93175414 fold)	regulate synapses, cognition, axonogenesis, and ion transmembrane transport	Diagnostic Biomarker
		brain tissue (Day 3 data)	animal	30	collected days 1/3/7/14/28 post-onset	increased (5.35935444 fold)	regulate synapses, cognition, axonogenesis, and ion transmembrane transport	Diagnostic Biomarker
		brain tissue (Day 7 data)	animal	30	collected days 1/3/7/14/28 post-onset	increased (3.618938373 fold)	regulate synapses, cognition, axonogenesis, and ion transmembrane transport	Diagnostic Biomarker

		brain tissue (Day 14 data)	animal	30	collected days 1/3/7/14/28 post-onset	increased (2.448706527 fold)	regulate synapses, cognition, axonogenesis, and ion transmembrane transport	Diagnostic Biomarker
**874-5p**		brain tissue (Day 1 data)	animal	30	collected days 1/3/7/14/28 post-onset	increased (1.309719192 fold)	regulate synapses, cognition, axonogenesis, and ion transmembrane transport	Diagnostic Biomarker
**5099**		brain tissue (Day 1 data)	animal	30	collected days 1/3/7/14/28 post-onset	increased (1.72882298 fold)	regulate synapses, cognition, axonogenesis, and ion transmembrane transport	Diagnostic Biomarker
		brain tissue (Day 14 data)	animal	30	collected days 1/3/7/14/28 post-onset	increased (0.688723381 fold)	regulate synapses, cognition, axonogenesis, and ion transmembrane transport	Diagnostic Biomarker
		brain tissue (Day 28 data)	animal	30	collected days 1/3/7/14/28 post-onset	increased (0.888716607 fold)	regulate synapses, cognition, axonogenesis, and ion transmembrane transport	Diagnostic Biomarker
**1291**		brain tissue (Day 1 data)	animal	30	collected days 1/3/7/14/28 post-onset	increased (7.661207159 fold)	regulate synapses, cognition, axonogenesis, and ion transmembrane transport	Diagnostic Biomarker
**5126**		brain tissue (Day 1 data)	animal	30	collected days 1/3/7/14/28 post-onset	increased (7.205824808 fold)	regulate synapses, cognition, axonogenesis, and ion transmembrane transport	Diagnostic Biomarker
		brain tissue (Day 3 data)	animal	30	collected days 1/3/7/14/28 post-onset	increased (3.737934045 fold)	regulate synapses, cognition, axonogenesis, and ion transmembrane transport	Diagnostic Biomarker
		brain tissue (Day 7 data)	animal	30	collected days 1/3/7/14/28 post-onset	increased (3.0007842 fold)	regulate synapses, cognition, axonogenesis, and ion transmembrane transport	Diagnostic Biomarker
**6236**		brain tissue (Day 1 data)	animal	30	collected days 1/3/7/14/28 post-onset	increased (7.10992639 fold)	regulate synapses, cognition, axonogenesis, and ion transmembrane transport	Diagnostic Biomarker
		brain tissue (Day 3 data)	animal	30	collected days 1/3/7/14/28 post-onset	increased (3.090817034 fold)	regulate synapses, cognition, axonogenesis, and ion transmembrane transport	Diagnostic Biomarker
		brain tissue (Day 28 data)	animal	30	collected days 1/3/7/14/28 post-onset	increased (3.974509133 fold)	regulate synapses, cognition, axonogenesis, and ion transmembrane transport	Diagnostic Biomarker
**714**		brain tissue (Day 1 data)	animal	30	collected days 1/3/7/14/28 post-onset	increased (7.090805137 fold)	regulate synapses, cognition, axonogenesis, and ion transmembrane transport	Diagnostic Biomarker
**6240**		brain tissue (Day 1 data)	animal	30	collected days 1/3/7/14/28 post-onset	increased (6.633378379 fold)	regulate synapses, cognition, axonogenesis, and ion transmembrane transport	Diagnostic Biomarker
		brain tissue (Day 3 data)	animal	30	collected days 1/3/7/14/28 post-onset	increased (3.031103152 fold)	regulate synapses, cognition, axonogenesis, and ion transmembrane transport	Diagnostic Biomarker
**6538**		brain tissue (Day 1 data)	animal	30	collected days 1/3/7/14/28 post-onset	increased (6.282590438 fold)	regulate synapses, cognition, axonogenesis, and ion transmembrane transport	Diagnostic Biomarker
**3062-5p**		brain tissue (Day 1 data)	animal	30	collected days 1/3/7/14/28 post-onset	increased (5.862195 fold)	regulate synapses, cognition, axonogenesis, and ion transmembrane transport	Diagnostic Biomarker
**7017-5p**		brain tissue (Day 1 data)	animal	30	collected days 1/3/7/14/28 post-onset	increased (5.522847492 fold)	regulate synapses, cognition, axonogenesis, and ion transmembrane transport	Diagnostic Biomarker

**7019-5p**	brain tissue (Day 1 data)	animal	30	collected days 1/3/7/14/28 post-onset	increased (5.522847492 fold)	regulate synapses, cognition, axonogenesis, and ion transmembrane transport	Diagnostic Biomarker
**709**	brain tissue (Day 1 data)	animal	30	collected days 1/3/7/14/28 post-onset	increased (5.416649455 fold)	regulate synapses, cognition, axonogenesis, and ion transmembrane transport	Diagnostic Biomarker
	brain tissue (Day 3 data)	animal	30	collected days 1/3/7/14/28 post-onset	increased (5.734541141 fold)	regulate synapses, cognition, axonogenesis, and ion transmembrane transport	Diagnostic Biomarker
**5121**	brain tissue (Day 1 data)	animal	30	collected days 1/3/7/14/28 post-onset	increased (5.262565618 fold)	regulate synapses, cognition, axonogenesis, and ion transmembrane transport	Diagnostic Biomarker
	brain tissue (Day 3 data)	animal	30	collected days 1/3/7/14/28 post-onset	increased (6.69133569 fold)	regulate synapses, cognition, axonogenesis, and ion transmembrane transport	Diagnostic Biomarker
**3473e**	brain tissue (Day 1 data)	animal	30	collected days 1/3/7/14/28 post-onset	increased (5.131178285 fold)	regulate synapses, cognition, axonogenesis, and ion transmembrane transport	Diagnostic Biomarker
	brain tissue (Day 28 data)	animal	30	collected days 1/3/7/14/28 post-onset	increased (4.819985251 fold)	regulate synapses, cognition, axonogenesis, and ion transmembrane transport	Diagnostic Biomarker
**192-3p**	brain tissue (Day 1 data)	animal	30	collected days 1/3/7/14/28 post-onset	increased (5.030703481 fold)	regulate synapses, cognition, axonogenesis, and ion transmembrane transport	Diagnostic Biomarker
	brain tissue (Day 3 data)	animal	30	collected days 1/3/7/14/28 post-onset	increased (3.277413307 fold)	regulate synapses, cognition, axonogenesis, and ion transmembrane transport	Diagnostic Biomarker
**12187-3p**	brain tissue (Day 1 data)	animal	30	collected days 1/3/7/14/28 post-onset	increased (4.615970544 fold)	regulate synapses, cognition, axonogenesis, and ion transmembrane transport	Diagnostic Biomarker
**1195**	brain tissue (Day 1 data)	animal	30	collected days 1/3/7/14/28 post-onset	increased (4.598497505 fold)	regulate synapses, cognition, axonogenesis, and ion transmembrane transport	Diagnostic Biomarker
	brain tissue (Day 3 data)	animal	30	collected days 1/3/7/14/28 post-onset	increased (6.211441002 fold)	regulate synapses, cognition, axonogenesis, and ion transmembrane transport	Diagnostic Biomarker
**712-5p**	brain tissue (Day 1 data)	animal	30	collected days 1/3/7/14/28 post-onset	increased (4.328549105 fold)	regulate synapses, cognition, axonogenesis, and ion transmembrane transport	Diagnostic Biomarker
**6238**	brain tissue (Day 1 data)	animal	30	collected days 1/3/7/14/28 post-onset	increased (3.999758129 fold)	regulate synapses, cognition, axonogenesis, and ion transmembrane transport	Diagnostic Biomarker
**7068-5p**	brain tissue (Day 1 data)	animal	30	collected days 1/3/7/14/28 post-onset	increased (3.782362743 fold)	regulate synapses, cognition, axonogenesis, and ion transmembrane transport	Diagnostic Biomarker
**92a-1-5p**	brain tissue (Day 1 data)	animal	30	collected days 1/3/7/14/28 post-onset	increased (3.689427238 fold)	regulate synapses, cognition, axonogenesis, and ion transmembrane transport	Diagnostic Biomarker
	brain tissue (Day 3 data)	animal	30	collected days 1/3/7/14/28 post-onset	increased (2.989080792 fold)	regulate synapses, cognition, axonogenesis, and ion transmembrane transport	Diagnostic Biomarker
	brain tissue (Day 7 data)	animal	30	collected days 1/3/7/14/28 post-onset	increased (2.573013497 fold)	regulate synapses, cognition, axonogenesis, and ion transmembrane transport	Diagnostic Biomarker

**3471**	brain tissue (Day 1 data)	animal	30	collected days 1/3/7/14/28 post-onset	increased (3.467828607 fold)	regulate synapses, cognition, axonogenesis, and ion transmembrane transport	Diagnostic Biomarker
	brain tissue (Day 3 data)	animal	30	collected days 1/3/7/14/28 post-onset	increased (2.067627622 fold)	regulate synapses, cognition, axonogenesis, and ion transmembrane transport	Diagnostic Biomarker
**144-5p**	brain tissue (Day 1 data)	animal	30	collected days 1/3/7/14/28 post-onset	increased (3.139187099 fold)	regulate synapses, cognition, axonogenesis, and ion transmembrane transport	Diagnostic Biomarker
	brain tissue (Day 3 data)	animal	30	collected days 1/3/7/14/28 post-onset	increased (3.446814306 fold)	regulate synapses, cognition, axonogenesis, and ion transmembrane transport	Diagnostic Biomarker
	brain tissue (Day 14 data)	animal	30	collected days 1/3/7/14/28 post-onset	decreased (-4.013753884 fold)	regulate inflammation, immune responses, and angiogenesis	Diagnostic Biomarker
	brain tissue (Day 28 data)	animal	30	collected days 1/3/7/14/28 post-onset	decreased (-6.124489012 fold)	regulate inflammation, immune responses, and angiogenesis	Diagnostic Biomarker
**690**	brain tissue (Day 1 data)	animal	30	collected days 1/3/7/14/28 post-onset	increased (3.102663719 fold)	regulate synapses, cognition, axonogenesis, and ion transmembrane transport	Diagnostic Biomarker
	brain tissue (Day 3 data)	animal	30	collected days 1/3/7/14/28 post-onset	increased (1.910768781 fold)	regulate synapses, cognition, axonogenesis, and ion transmembrane transport	Diagnostic Biomarker
**144-3p**	brain tissue (Day 1 data)	animal	30	collected days 1/3/7/14/28 post-onset	increased (3.083642197 fold)	regulate synapses, cognition, axonogenesis, and ion transmembrane transport	Diagnostic Biomarker
	brain tissue (Day 3 data)	animal	30	collected days 1/3/7/14/28 post-onset	increased (4.079524819 fold)	regulate synapses, cognition, axonogenesis, and ion transmembrane transport	Diagnostic Biomarker
	brain tissue (Day 28 data)	animal	30	collected days 1/3/7/14/28 post-onset	decreased (-4.041274705 fold)	regulate inflammation, immune responses, and angiogenesis	Diagnostic Biomarker
**3470b**	brain tissue (Day 1 data)	animal	30	collected days 1/3/7/14/28 post-onset	increased (3.036634338 fold)	regulate synapses, cognition, axonogenesis, and ion transmembrane transport	Diagnostic Biomarker
	brain tissue (Day 3 data)	animal	30	collected days 1/3/7/14/28 post-onset	increased (1.716279265 fold)	regulate synapses, cognition, axonogenesis, and ion transmembrane transport	Diagnostic Biomarker
**3470a**	brain tissue (Day 1 data)	animal	30	collected days 1/3/7/14/28 post-onset	increased (2.72755704 fold)	regulate synapses, cognition, axonogenesis, and ion transmembrane transport	Diagnostic Biomarker
	brain tissue (Day 3 data)	animal	30	collected days 1/3/7/14/28 post-onset	increased (2.133115062 fold)	regulate synapses, cognition, axonogenesis, and ion transmembrane transport	Diagnostic Biomarker
**199b-5p**	brain tissue (Day 1 data)	animal	30	collected days 1/3/7/14/28 post-onset	increased (2.674023734 fold)	regulate synapses, cognition, axonogenesis, and ion transmembrane transport	Diagnostic Biomarker
	brain tissue (Day 7 data)	animal	30	collected days 1/3/7/14/28 post-onset	increased (2.560541746 fold)	regulate synapses, cognition, axonogenesis, and ion transmembrane transport	Diagnostic Biomarker
**21a-3p**	brain tissue (Day 1 data)	animal	30	collected days 1/3/7/14/28 post-onset	increased (2.517323432 fold)	regulate synapses, cognition, axonogenesis, and ion transmembrane transport	Diagnostic Biomarker

		brain tissue (Day 3 data)	animal	30	collected days 1/3/7/14/28 post-onset	increased (3.714839983 fold)	regulate synapses, cognition, axonogenesis, and ion transmembrane transport	Diagnostic Biomarker
		brain tissue (Day 7 data)	animal	30	collected days 1/3/7/14/28 post-onset	increased (3.680589758 fold)	regulate synapses, cognition, axonogenesis, and ion transmembrane transport	Diagnostic Biomarker
		brain tissue (Day 14 data)	animal	30	collected days 1/3/7/14/28 post-onset	increased (3.44775709 fold)	regulate synapses, cognition, axonogenesis, and ion transmembrane transport	Diagnostic Biomarker
		brain tissue (Day 28 data)	animal	30	collected days 1/3/7/14/28 post-onset	increased (1.861420404 fold)	regulate synapses, cognition, axonogenesis, and ion transmembrane transport	Diagnostic Biomarker
**494-3p**		brain tissue (Day 1 data)	animal	30	collected days 1/3/7/14/28 post-onset	increased (2.278993469 fold)	regulate synapses, cognition, axonogenesis, and ion transmembrane transport	Diagnostic Biomarker
		brain tissue (Day 28 data)	animal	30	collected days 1/3/7/14/28 post-onset	decreased (-1.806548289 fold)	regulate inflammation, immune responses, and angiogenesis	Diagnostic Biomarker
**3085-3p**		brain tissue (Day 1 data)	animal	30	collected days 1/3/7/14/28 post-onset	increased (2.243406461 fold)	regulate synapses, cognition, axonogenesis, and ion transmembrane transport	Diagnostic Biomarker
**299a-5p**		brain tissue (Day 1 data)	animal	30	collected days 1/3/7/14/28 post-onset	increased (2.221442914 fold)	regulate synapses, cognition, axonogenesis, and ion transmembrane transport	Diagnostic Biomarker
		brain tissue (Day 7 data)	animal	30	collected days 1/3/7/14/28 post-onset	decreased (-1.612095539 fold)	regulate inflammation, immune responses, and angiogenesis	Diagnostic Biomarker
		brain tissue (Day 14 data)	animal	30	collected days 1/3/7/14/28 post-onset	decreased (-0.843051539 fold)	regulate inflammation, immune responses, and angiogenesis	Diagnostic Biomarker
		brain tissue (Day 28 data)	animal	30	collected days 1/3/7/14/28 post-onset	decreased (-1.217014769 fold)	regulate inflammation, immune responses, and angiogenesis	Diagnostic Biomarker
**299b-5p**		brain tissue (Day 1 data)	animal	30	collected days 1/3/7/14/28 post-onset	increased (2.220812788 fold)	regulate synapses, cognition, axonogenesis, and ion transmembrane transport	Diagnostic Biomarker
		brain tissue (Day 7 data)	animal	30	collected days 1/3/7/14/28 post-onset	decreased (-1.609543726 fold)	regulate inflammation, immune responses, and angiogenesis	Diagnostic Biomarker
		brain tissue (Day 14 data)	animal	30	collected days 1/3/7/14/28 post-onset	decreased (-0.849328257 fold)	regulate inflammation, immune responses, and angiogenesis	Diagnostic Biomarker
		brain tissue (Day 28 data)	animal	30	collected days 1/3/7/14/28 post-onset	decreased (-1.21258852 fold)	regulate inflammation, immune responses, and angiogenesis	Diagnostic Biomarker
**7667-3p**		brain tissue (Day 1 data)	animal	30	collected days 1/3/7/14/28 post-onset	increased (2.078041023 fold)	regulate synapses, cognition, axonogenesis, and ion transmembrane transport	Diagnostic Biomarker
**107-3p**		brain tissue (Day 1 data)	animal	30	collected days 1/3/7/14/28 post-onset	increased (2.035956423 fold)	regulate synapses, cognition, axonogenesis, and ion transmembrane transport	Diagnostic Biomarker
		brain tissue (Day 3 data)	animal	30	collected days 1/3/7/14/28 post-onset	increased (1.710559664 fold)	regulate synapses, cognition, axonogenesis, and ion transmembrane transport	Diagnostic Biomarker

	brain tissue (Day 7 data)	animal	30	collected days 1/3/7/14/28 post-onset	increased (1.054270389 fold)	regulate synapses, cognition, axonogenesis, and ion transmembrane transport	Diagnostic Biomarker
**6540-3p**	brain tissue (Day 1 data)	animal	30	collected days 1/3/7/14/28 post-onset	increased (1.999426731 fold)	regulate synapses, cognition, axonogenesis, and ion transmembrane transport	Diagnostic Biomarker
**6970-5p**	brain tissue (Day 1 data)	animal	30	collected days 1/3/7/14/28 post-onset	increased (1.979922624 fold)	regulate synapses, cognition, axonogenesis, and ion transmembrane transport	Diagnostic Biomarker
**26a-5p**	brain tissue (Day 1 data)	animal	30	collected days 1/3/7/14/28 post-onset	increased (1.888375564 fold)	regulate synapses, cognition, axonogenesis, and ion transmembrane transport	Diagnostic Biomarker
**6715-5p**	brain tissue (Day 1 data)	animal	30	collected days 1/3/7/14/28 post-onset	increased (1.888298526 fold)	regulate synapses, cognition, axonogenesis, and ion transmembrane transport	Diagnostic Biomarker
**16-1-3p**	brain tissue (Day 1 data)	animal	30	collected days 1/3/7/14/28 post-onset	increased (1.816810751 fold)	regulate synapses, cognition, axonogenesis, and ion transmembrane transport	Diagnostic Biomarker
	brain tissue (Day 3 data)	animal	30	collected days 1/3/7/14/28 post-onset	increased (2.653665902 fold)	regulate synapses, cognition, axonogenesis, and ion transmembrane transport	Diagnostic Biomarker
	brain tissue (Day 14 data)	animal	30	collected days 1/3/7/14/28 post-onset	increased (2.162155868 fold)	regulate synapses, cognition, axonogenesis, and ion transmembrane transport	Diagnostic Biomarker
**1843a-3p**	brain tissue (Day 1 data)	animal	30	collected days 1/3/7/14/28 post-onset	increased (1.720954063 fold)	regulate synapses, cognition, axonogenesis, and ion transmembrane transport	Diagnostic Biomarker
**27a-5p**	brain tissue (Day 1 data)	animal	30	collected days 1/3/7/14/28 post-onset	increased (1.561432513 fold)	regulate synapses, cognition, axonogenesis, and ion transmembrane transport	Diagnostic Biomarker
	brain tissue (Day 3 data)	animal	30	collected days 1/3/7/14/28 post-onset	increased (2.138730569 fold)	regulate synapses, cognition, axonogenesis, and ion transmembrane transport	Diagnostic Biomarker
	brain tissue (Day 14 data)	animal	30	collected days 1/3/7/14/28 post-onset	increased (1.713464434 fold)	regulate synapses, cognition, axonogenesis, and ion transmembrane transport	Diagnostic Biomarker
**1968-5p**	brain tissue (Day 1 data)	animal	30	collected days 1/3/7/14/28 post-onset	increased (1.377089465 fold)	regulate synapses, cognition, axonogenesis, and ion transmembrane transport	Diagnostic Biomarker
**21a-5p**	brain tissue (Day 1 data)	animal	30	collected days 1/3/7/14/28 post-onset	increased (1.277313642 fold)	regulate synapses, cognition, axonogenesis, and ion transmembrane transport	Diagnostic Biomarker
	brain tissue (Day 3 data)	animal	30	collected days 1/3/7/14/28 post-onset	increased (3.098950671 fold)	regulate synapses, cognition, axonogenesis, and ion transmembrane transport	Diagnostic Biomarker
	brain tissue (Day 7 data)	animal	30	collected days 1/3/7/14/28 post-onset	increased (3.231364902 fold)	regulate synapses, cognition, axonogenesis, and ion transmembrane transport	Diagnostic Biomarker
	brain tissue (Day 14 data)	animal	30	collected days 1/3/7/14/28 post-onset	increased (2.681598963 fold)	regulate synapses, cognition, axonogenesis, and ion transmembrane transport	Diagnostic Biomarker
	brain tissue (Day 28 data)	animal	30	collected days 1/3/7/14/28 post-onset	increased (1.44006729 fold)	regulate synapses, cognition, axonogenesis, and ion transmembrane transport	Diagnostic Biomarker

**142a-5p**	brain tissue (Day 1 data)	animal	30	collected days 1/3/7/14/28 post-onset	increased (1.273335119 fold)	regulate synapses, cognition, axonogenesis, and ion transmembrane transport	Diagnostic Biomarker
	brain tissue (Day 3 data)	animal	30	collected days 1/3/7/14/28 post-onset	increased (1.865173496 fold)	regulate synapses, cognition, axonogenesis, and ion transmembrane transport	Diagnostic Biomarker
	brain tissue (Day 7 data)	animal	30	collected days 1/3/7/14/28 post-onset	increased (2.161263898 fold)	regulate synapses, cognition, axonogenesis, and ion transmembrane transport	Diagnostic Biomarker
	brain tissue (Day 14 data)	animal	30	collected days 1/3/7/14/28 post-onset	increased (2.955233597 fold)	regulate synapses, cognition, axonogenesis, and ion transmembrane transport	Diagnostic Biomarker
	brain tissue (Day 28 data)	animal	30	collected days 1/3/7/14/28 post-onset	increased (3.051534263 fold)	regulate synapses, cognition, axonogenesis, and ion transmembrane transport	Diagnostic Biomarker
**215-5p**	brain tissue (Day 1 data)	animal	30	collected days 1/3/7/14/28 post-onset	increased (1.272945456 fold)	regulate synapses, cognition, axonogenesis, and ion transmembrane transport	Diagnostic Biomarker
**187-5p**	brain tissue (Day 1 data)	animal	30	collected days 1/3/7/14/28 post-onset	increased (1.271931871 fold)	regulate synapses, cognition, axonogenesis, and ion transmembrane transport	Diagnostic Biomarker
**1943-5p**	brain tissue (Day 1 data)	animal	30	collected days 1/3/7/14/28 post-onset	increased (1.159880239 fold)	regulate synapses, cognition, axonogenesis, and ion transmembrane transport	Diagnostic Biomarker
	brain tissue (Day 7 data)	animal	30	collected days 1/3/7/14/28 post-onset	increased (1.582539954 fold)	regulate synapses, cognition, axonogenesis, and ion transmembrane transport	Diagnostic Biomarker
**3535**	brain tissue (Day 1 data)	animal	30	collected days 1/3/7/14/28 post-onset	increased (1.120230644 fold)	regulate synapses, cognition, axonogenesis, and ion transmembrane transport	Diagnostic Biomarker
	brain tissue (Day 28 data)	animal	30	collected days 1/3/7/14/28 post-onset	decreased (-1.00403615 fold)	regulate inflammation, immune responses, and angiogenesis	Diagnostic Biomarker
**3083b-3p**	brain tissue (Day 1 data)	animal	30	collected days 1/3/7/14/28 post-onset	increased (1.1112868 fold)	regulate synapses, cognition, axonogenesis, and ion transmembrane transport	Diagnostic Biomarker
**218-1-3p**	brain tissue (Day 1 data)	animal	30	collected days 1/3/7/14/28 post-onset	increased (1.071416926 fold)	regulate synapses, cognition, axonogenesis, and ion transmembrane transport	Diagnostic Biomarker
**374b-5p**	brain tissue (Day 1 data)	animal	30	collected days 1/3/7/14/28 post-onset	increased (1.052879181 fold)	regulate synapses, cognition, axonogenesis, and ion transmembrane transport	Diagnostic Biomarker
	brain tissue (Day 7 data)	animal	30	collected days 1/3/7/14/28 post-onset	increased (1.171004828 fold)	regulate synapses, cognition, axonogenesis, and ion transmembrane transport	Diagnostic Biomarker
**374c-5p**	brain tissue (Day 1 data)	animal	30	collected days 1/3/7/14/28 post-onset	increased (1.052879181 fold)	regulate synapses, cognition, axonogenesis, and ion transmembrane transport	Diagnostic Biomarker
	brain tissue (Day 7 data)	animal	30	collected days 1/3/7/14/28 post-onset	increased (1.171004828 fold)	regulate synapses, cognition, axonogenesis, and ion transmembrane transport	Diagnostic Biomarker
**7235-3p**	brain tissue (Day 1 data)	animal	30	collected days 1/3/7/14/28 post-onset	increased (1.002022597 fold)	regulate synapses, cognition, axonogenesis, and ion transmembrane transport	Diagnostic Biomarker

**10a-5p**	brain tissue (Day 1 data)	animal	30	collected days 1/3/7/14/28 post-onset	increased (0.968012874 fold)	regulate synapses, cognition, axonogenesis, and ion transmembrane transport	Diagnostic Biomarker
	brain tissue (Day 3 data)	animal	30	collected days 1/3/7/14/28 post-onset	increased (1.041105536 fold)	regulate synapses, cognition, axonogenesis, and ion transmembrane transport	Diagnostic Biomarker
	brain tissue (Day 7 data)	animal	30	collected days 1/3/7/14/28 post-onset	increased (4.10013929 fold)	regulate synapses, cognition, axonogenesis, and ion transmembrane transport	Diagnostic Biomarker
	brain tissue (Day 14 data)	animal	30	collected days 1/3/7/14/28 post-onset	increased (3.581457466 fold)	regulate synapses, cognition, axonogenesis, and ion transmembrane transport	Diagnostic Biomarker
	brain tissue (Day 28 data)	animal	30	collected days 1/3/7/14/28 post-onset	increased (3.802171146 fold)	regulate synapses, cognition, axonogenesis, and ion transmembrane transport	Diagnostic Biomarker
**664-5p**	brain tissue (Day 1 data)	animal	30	collected days 1/3/7/14/28 post-onset	increased (0.923616004 fold)	regulate synapses, cognition, axonogenesis, and ion transmembrane transport	Diagnostic Biomarker
	brain tissue (Day 7 data)	animal	30	collected days 1/3/7/14/28 post-onset	increased (0.891110196 fold)	regulate synapses, cognition, axonogenesis, and ion transmembrane transport	Diagnostic Biomarker
	brain tissue (Day 14 data)	animal	30	collected days 1/3/7/14/28 post-onset	increased (0.573939935 fold)	regulate synapses, cognition, axonogenesis, and ion transmembrane transport	Diagnostic Biomarker
**434-3p**	brain tissue (Day 1 data)	animal	30	collected days 1/3/7/14/28 post-onset	increased (0.922206231 fold)	regulate synapses, cognition, axonogenesis, and ion transmembrane transport	Diagnostic Biomarker
**496a-3p**	brain tissue (Day 1 data)	animal	30	collected days 1/3/7/14/28 post-onset	increased (0.907385712 fold)	regulate synapses, cognition, axonogenesis, and ion transmembrane transport	Diagnostic Biomarker
	brain tissue (Day 7 data)	animal	30	collected days 1/3/7/14/28 post-onset	decreased (-0.997484649 fold)	regulate inflammation, immune responses, and angiogenesis	Diagnostic Biomarker
	brain tissue (Day 14 data)	animal	30	collected days 1/3/7/14/28 post-onset	decreased (-0.567076998 fold)	regulate inflammation, immune responses, and angiogenesis	Diagnostic Biomarker
	brain tissue (Day 28 data)	animal	30	collected days 1/3/7/14/28 post-onset	decreased (-0.60994124 fold)	regulate inflammation, immune responses, and angiogenesis	Diagnostic Biomarker
**221-5p**	brain tissue (Day 1 data)	animal	30	collected days 1/3/7/14/28 post-onset	increased (0.877157477 fold)	regulate synapses, cognition, axonogenesis, and ion transmembrane transport	Diagnostic Biomarker
	brain tissue (Day 7 data)	animal	30	collected days 1/3/7/14/28 post-onset	decreased (-0.964491722 fold)	regulate inflammation, immune responses, and angiogenesis	Diagnostic Biomarker
**3057-5p**	brain tissue (Day 1 data)	animal	30	collected days 1/3/7/14/28 post-onset	increased (0.815616871 fold)	regulate synapses, cognition, axonogenesis, and ion transmembrane transport	Diagnostic Biomarker
	brain tissue (Day 28 data)	animal	30	collected days 1/3/7/14/28 post-onset	increased (0.871258104 fold)	regulate synapses, cognition, axonogenesis, and ion transmembrane transport	Diagnostic Biomarker
**3068-3p**	brain tissue (Day 1 data)	animal	30	collected days 1/3/7/14/28 post-onset	increased (0.776016295 fold)	regulate synapses, cognition, axonogenesis, and ion transmembrane transport	Diagnostic Biomarker

**145a-3p**	brain tissue (Day 1 data)	animal	30	collected days 1/3/7/14/28 post-onset	increased (0.752238327 fold)	regulate synapses, cognition, axonogenesis, and ion transmembrane transport	Diagnostic Biomarker
	brain tissue (Day 3 data)	animal	30	collected days 1/3/7/14/28 post-onset	increased (1.215172524 fold)	regulate synapses, cognition, axonogenesis, and ion transmembrane transport	Diagnostic Biomarker
	brain tissue (Day 14 data)	animal	30	collected days 1/3/7/14/28 post-onset	increased (0.918647557 fold)	regulate synapses, cognition, axonogenesis, and ion transmembrane transport	Diagnostic Biomarker
	brain tissue (Day 28 data)	animal	30	collected days 1/3/7/14/28 post-onset	increased (1.10262935 fold)	regulate synapses, cognition, axonogenesis, and ion transmembrane transport	Diagnostic Biomarker
**412-3p**	brain tissue (Day 1 data)	animal	30	collected days 1/3/7/14/28 post-onset	increased (0.751264432 fold)	regulate synapses, cognition, axonogenesis, and ion transmembrane transport	Diagnostic Biomarker
**1983**	brain tissue (Day 1 data)	animal	30	collected days 1/3/7/14/28 post-onset	increased (0.747023525 fold)	regulate synapses, cognition, axonogenesis, and ion transmembrane transport	Diagnostic Biomarker
**6896-5p**	brain tissue (Day 1 data)	animal	30	collected days 1/3/7/14/28 post-onset	increased (0.693038221 fold)	regulate synapses, cognition, axonogenesis, and ion transmembrane transport	Diagnostic Biomarker
**98-5p**	brain tissue (Day 1 data)	animal	30	collected days 1/3/7/14/28 post-onset	increased (0.689833762 fold)	regulate synapses, cognition, axonogenesis, and ion transmembrane transport	Diagnostic Biomarker
	brain tissue (Day 7 data)	animal	30	collected days 1/3/7/14/28 post-onset	increased (0.96527511 fold)	regulate synapses, cognition, axonogenesis, and ion transmembrane transport	Diagnostic Biomarker
**345-3p**	brain tissue (Day 1 data)	animal	30	collected days 1/3/7/14/28 post-onset	increased (0.688744037 fold)	regulate synapses, cognition, axonogenesis, and ion transmembrane transport	Diagnostic Biomarker
	brain tissue (Day 7 data)	animal	30	collected days 1/3/7/14/28 post-onset	increased (1.358973145 fold)	regulate synapses, cognition, axonogenesis, and ion transmembrane transport	Diagnostic Biomarker
**338-5p**	brain tissue (Day 1 data)	animal	30	collected days 1/3/7/14/28 post-onset	increased (0.641908551 fold)	regulate synapses, cognition, axonogenesis, and ion transmembrane transport	Diagnostic Biomarker
**28a-3p**	brain tissue (Day 1 data)	animal	30	collected days 1/3/7/14/28 post-onset	increased (0.635686838 fold)	regulate synapses, cognition, axonogenesis, and ion transmembrane transport	Diagnostic Biomarker
	brain tissue (Day 7 data)	animal	30	collected days 1/3/7/14/28 post-onset	increased (1.204501793 fold)	regulate synapses, cognition, axonogenesis, and ion transmembrane transport	Diagnostic Biomarker
	brain tissue (Day 14 data)	animal	30	collected days 1/3/7/14/28 post-onset	increased (0.734879066 fold)	regulate synapses, cognition, axonogenesis, and ion transmembrane transport	Diagnostic Biomarker
**30c-2-3p**	brain tissue (Day 1 data)	animal	30	collected days 1/3/7/14/28 post-onset	increased (0.630721789 fold)	regulate synapses, cognition, axonogenesis, and ion transmembrane transport	Diagnostic Biomarker
**103-3p**	brain tissue (Day 1 data)	animal	30	collected days 1/3/7/14/28 post-onset	increased (0.595544317 fold)	regulate synapses, cognition, axonogenesis, and ion transmembrane transport	Diagnostic Biomarker
	brain tissue (Day 28 data)	animal	30	collected days 1/3/7/14/28 post-onset	decreased (-0.781370483 fold)	regulate inflammation, immune responses, and angiogenesis	Diagnostic Biomarker

**146a-5p**	brain tissue (Day 1 data)	animal	30	collected days 1/3/7/14/28 post-onset	increased (0.591775547 fold)	regulate synapses, cognition, axonogenesis, and ion transmembrane transport	Diagnostic Biomarker
	brain tissue (Day 7 data)	animal	30	collected days 1/3/7/14/28 post-onset	increased (2.152369357 fold)	regulate synapses, cognition, axonogenesis, and ion transmembrane transport	Diagnostic Biomarker
	brain tissue (Day 14 data)	animal	30	collected days 1/3/7/14/28 post-onset	increased (2.580930184 fold)	regulate synapses, cognition, axonogenesis, and ion transmembrane transport	Diagnostic Biomarker
	brain tissue (Day 28 data)	animal	30	collected days 1/3/7/14/28 post-onset	increased (2.023713665 fold)	regulate synapses, cognition, axonogenesis, and ion transmembrane transport	Diagnostic Biomarker
**204-5p**	brain tissue (Day 1 data)	animal	30	collected days 1/3/7/14/28 post-onset	increased (0.487967158 fold)	regulate synapses, cognition, axonogenesis, and ion transmembrane transport	Diagnostic Biomarker
**100-5p**	brain tissue (Day 1 data)	animal	30	collected days 1/3/7/14/28 post-onset	increased (0.433544233 fold)	regulate synapses, cognition, axonogenesis, and ion transmembrane transport	Diagnostic Biomarker
**30d-5p**	brain tissue (Day 1 data)	animal	30	collected days 1/3/7/14/28 post-onset	decreased (-0.419863259 fold)	regulate inflammation, immune responses, and angiogenesis	Diagnostic Biomarker
**let-7b-5p**	brain tissue (Day 1 data)	animal	30	collected days 1/3/7/14/28 post-onset	decreased (-0.448218863 fold)	regulate inflammation, immune responses, and angiogenesis	Diagnostic Biomarker
**375-3p**	brain tissue (Day 1 data)	animal	30	collected days 1/3/7/14/28 post-onset	decreased (-0.451250627 fold)	regulate inflammation, immune responses, and angiogenesis	Diagnostic Biomarker
**337-5p**	brain tissue (Day 1 data)	animal	30	collected days 1/3/7/14/28 post-onset	decreased (-0.465506909 fold)	regulate inflammation, immune responses, and angiogenesis	Diagnostic Biomarker
	brain tissue (Day 14 data)	animal	30	collected days 1/3/7/14/28 post-onset	decreased (-0.718409053 fold)	regulate inflammation, immune responses, and angiogenesis	Diagnostic Biomarker
	brain tissue (Day 28 data)	animal	30	collected days 1/3/7/14/28 post-onset	decreased (-1.163818576 fold)	regulate inflammation, immune responses, and angiogenesis	Diagnostic Biomarker
**431-5p**	brain tissue (Day 1 data)	animal	30	collected days 1/3/7/14/28 post-onset	decreased (-0.465983056 fold)	regulate inflammation, immune responses, and angiogenesis	Diagnostic Biomarker
	brain tissue (Day 7 data)	animal	30	collected days 1/3/7/14/28 post-onset	decreased (-0.974552542 fold)	regulate inflammation, immune responses, and angiogenesis	Diagnostic Biomarker
**664-3p**	brain tissue (Day 1 data)	animal	30	collected days 1/3/7/14/28 post-onset	decreased (-0.507311541 fold)	regulate inflammation, immune responses, and angiogenesis	Diagnostic Biomarker
	brain tissue (Day 14 data)	animal	30	collected days 1/3/7/14/28 post-onset	decreased (-0.440561211 fold)	regulate inflammation, immune responses, and angiogenesis	Diagnostic Biomarker
**129-1-3p**	brain tissue (Day 1 data)	animal	30	collected days 1/3/7/14/28 post-onset	decreased (-0.509476106 fold)	regulate inflammation, immune responses, and angiogenesis	Diagnostic Biomarker
	brain tissue (Day 7 data)	animal	30	collected days 1/3/7/14/28 post-onset	decreased (-1.090742854 fold)	regulate inflammation, immune responses, and angiogenesis	Diagnostic Biomarker

		brain tissue (Day 14 data)	animal	30	collected days 1/3/7/14/28 post-onset	decreased (-0.716560022 fold)	regulate inflammation, immune responses, and angiogenesis	Diagnostic Biomarker
		brain tissue (Day 28 data)	animal	30	collected days 1/3/7/14/28 post-onset	decreased (-0.76124312 fold)	regulate inflammation, immune responses, and angiogenesis	Diagnostic Biomarker
**708-5p**		brain tissue (Day 1 data)	animal	30	collected days 1/3/7/14/28 post-onset	decreased (-0.540551904 fold)	regulate inflammation, immune responses, and angiogenesis	Diagnostic Biomarker
**6540-5p**		brain tissue (Day 1 data)	animal	30	collected days 1/3/7/14/28 post-onset	decreased (-0.56788721 fold)	regulate inflammation, immune responses, and angiogenesis	Diagnostic Biomarker
**378a-5p**		brain tissue (Day 1 data)	animal	30	collected days 1/3/7/14/28 post-onset	decreased (-0.573605553 fold)	regulate inflammation, immune responses, and angiogenesis	Diagnostic Biomarker
**330-3p**		brain tissue (Day 1 data)	animal	30	collected days 1/3/7/14/28 post-onset	decreased (-0.576475164 fold)	regulate inflammation, immune responses, and angiogenesis	Diagnostic Biomarker
**23a-3p**		brain tissue (Day 1 data)	animal	30	collected days 1/3/7/14/28 post-onset	decreased (-0.579542711 fold)	regulate inflammation, immune responses, and angiogenesis	Diagnostic Biomarker
**30b-5p**		brain tissue (Day 1 data)	animal	30	collected days 1/3/7/14/28 post-onset	decreased (-0.596052787 fold)	regulate inflammation, immune responses, and angiogenesis	Diagnostic Biomarker
**31-5p**		brain tissue (Day 1 data)	animal	30	collected days 1/3/7/14/28 post-onset	decreased (-0.598357125 fold)	regulate inflammation, immune responses, and angiogenesis	Diagnostic Biomarker
		brain tissue (Day 28 data)	animal	30	collected days 1/3/7/14/28 post-onset	decreased (-0.712712129 fold)	regulate inflammation, immune responses, and angiogenesis	Diagnostic Biomarker
**351-5p**		brain tissue (Day 1 data)	animal	30	collected days 1/3/7/14/28 post-onset	decreased (-0.605324351 fold)	regulate inflammation, immune responses, and angiogenesis	Diagnostic Biomarker
**370-3p**		brain tissue (Day 1 data)	animal	30	collected days 1/3/7/14/28 post-onset	decreased (-0.637169833 fold)	regulate inflammation, immune responses, and angiogenesis	Diagnostic Biomarker
**652-3p**		brain tissue (Day 1 data)	animal	30	collected days 1/3/7/14/28 post-onset	decreased (-0.639262701 fold)	regulate inflammation, immune responses, and angiogenesis	Diagnostic Biomarker
		brain tissue (Day 7 data)	animal	30	collected days 1/3/7/14/28 post-onset	decreased (-1.240538525 fold)	regulate inflammation, immune responses, and angiogenesis	Diagnostic Biomarker
**let-7d-5p**		brain tissue (Day 1 data)	animal	30	collected days 1/3/7/14/28 post-onset	decreased (-0.64009306 fold)	regulate inflammation, immune responses, and angiogenesis	Diagnostic Biomarker
**7a-2-3p**		brain tissue (Day 1 data)	animal	30	collected days 1/3/7/14/28 post-onset	decreased (-0.646195558 fold)	regulate inflammation, immune responses, and angiogenesis	Diagnostic Biomarker
		brain tissue (Day 7 data)	animal	30	collected days 1/3/7/14/28 post-onset	decreased(-0.762927662 fold)	regulate inflammation, immune responses, and angiogenesis	Diagnostic Biomarker
**425-3p**		brain tissue (Day 1 data)	animal	30	collected days 1/3/7/14/28 post-onset	decreased (-0.657687425 fold)	regulate inflammation, immune responses, and angiogenesis	Diagnostic Biomarker

	brain tissue (Day 14 data)	animal	30	collected days 1/3/7/14/28 post-onset	increased (0.716667753 fold)	regulate synapses, cognition, axonogenesis, and ion transmembrane transport	Diagnostic Biomarker
**1981-5p**	brain tissue (Day 1 data)	animal	30	collected days 1/3/7/14/28 post-onset	decreased (-0.66497808 fold)	regulate inflammation, immune responses, and angiogenesis	Diagnostic Biomarker
**674-5p**	brain tissue (Day 1 data)	animal	30	collected days 1/3/7/14/28 post-onset	decreased (-0.676305895 fold)	regulate inflammation, immune responses, and angiogenesis	Diagnostic Biomarker
**7019-3p**	brain tissue (Day 1 data)	animal	30	collected days 1/3/7/14/28 post-onset	decreased (-0.699431561 fold)	regulate inflammation, immune responses, and angiogenesis	Diagnostic Biomarker
	brain tissue (Day 7 data)	animal	30	collected days 1/3/7/14/28 post-onset	decreased (-0.994610984 fold)	regulate inflammation, immune responses, and angiogenesis	Diagnostic Biomarker
**331-3p**	brain tissue (Day 1 data)	animal	30	collected days 1/3/7/14/28 post-onset	decreased (-0.731264176 fold)	regulate inflammation, immune responses, and angiogenesis	Diagnostic Biomarker
	brain tissue (Day 7 data)	animal	30	collected days 1/3/7/14/28 post-onset	decreased (-0.81890619 fold)	regulate inflammation, immune responses, and angiogenesis	Diagnostic Biomarker
	brain tissue (Day 28 data)	animal	30	collected days 1/3/7/14/28 post-onset	decreased (-1.422386657 fold)	regulate inflammation, immune responses, and angiogenesis	Diagnostic Biomarker
**669c-5p**	brain tissue (Day 1 data)	animal	30	collected days 1/3/7/14/28 post-onset	decreased (-0.731771969 fold)	regulate inflammation, immune responses, and angiogenesis	Diagnostic Biomarker
**let-7g-3p**	brain tissue (Day 1 data)	animal	30	collected days 1/3/7/14/28 post-onset	decreased (-0.750048293 fold)	regulate inflammation, immune responses, and angiogenesis	Diagnostic Biomarker
**128-1-5p**	brain tissue (Day 1 data)	animal	30	collected days 1/3/7/14/28 post-onset	decreased (-0.767450565 fold)	regulate inflammation, immune responses, and angiogenesis	Diagnostic Biomarker
	brain tissue (Day 7 data)	animal	30	collected days 1/3/7/14/28 post-onset	decreased (-0.947714313 fold)	regulate inflammation, immune responses, and angiogenesis	Diagnostic Biomarker
	brain tissue (Day 14 data)	animal	30	collected days 1/3/7/14/28 post-onset	decreased (-0.802969883 fold)	regulate inflammation, immune responses, and angiogenesis	Diagnostic Biomarker
	brain tissue (Day 28 data)	animal	30	collected days 1/3/7/14/28 post-onset	decreased (-0.837441843 fold)	regulate inflammation, immune responses, and angiogenesis	Diagnostic Biomarker
**145a-5p**	brain tissue (Day 1 data)	animal	30	collected days 1/3/7/14/28 post-onset	decreased (-0.769466302 fold)	regulate inflammation, immune responses, and angiogenesis	Diagnostic Biomarker
	brain tissue (Day 28 data)	animal	30	collected days 1/3/7/14/28 post-onset	increased (0.721703833 fold)	regulate synapses, cognition, axonogenesis, and ion transmembrane transport	Diagnostic Biomarker
**3093-5p**	brain tissue (Day 1 data)	animal	30	collected days 1/3/7/14/28 post-onset	decreased (-0.770869009 fold)	regulate inflammation, immune responses, and angiogenesis	Diagnostic Biomarker
**935**	brain tissue (Day 1 data)	animal	30	collected days 1/3/7/14/28 post-onset	decreased (-0.782832963 fold)	regulate inflammation, immune responses, and angiogenesis	Diagnostic Biomarker

	brain tissue (Day 7 data)	animal	30	collected days 1/3/7/14/28 post-onset	decreased (-0.900268101 fold)	regulate inflammation, immune responses, and angiogenesis	Diagnostic Biomarker
**673-3p**	brain tissue (Day 1 data)	animal	30	collected days 1/3/7/14/28 post-onset	decreased (-0.783745113 fold)	regulate inflammation, immune responses, and angiogenesis	Diagnostic Biomarker
**3068-5p**	brain tissue (Day 1 data)	animal	30	collected days 1/3/7/14/28 post-onset	decreased (-0.7977288 fold)	regulate inflammation, immune responses, and angiogenesis	Diagnostic Biomarker
**7047-3p**	brain tissue (Day 1 data)	animal	30	collected days 1/3/7/14/28 post-onset	decreased (-0.798485984 fold)	regulate inflammation, immune responses, and angiogenesis	Diagnostic Biomarker
	brain tissue (Day 14 data)	animal	30	collected days 1/3/7/14/28 post-onset	decreased (-0.843464376 fold)	regulate inflammation, immune responses, and angiogenesis	Diagnostic Biomarker
**130b-5p**	brain tissue (Day 1 data)	animal	30	collected days 1/3/7/14/28 post-onset	decreased (-0.883235849 fold)	regulate inflammation, immune responses, and angiogenesis	Diagnostic Biomarker
	brain tissue (Day 7 data)	animal	30	collected days 1/3/7/14/28 post-onset	decreased (-0.9096568 fold)	regulate inflammation, immune responses, and angiogenesis	Diagnostic Biomarker
**8103**	brain tissue (Day 1 data)	animal	30	collected days 1/3/7/14/28 post-onset	decreased (-0.890795004 fold)	regulate inflammation, immune responses, and angiogenesis	Diagnostic Biomarker
**877-3p**	brain tissue (Day 1 data)	animal	30	collected days 1/3/7/14/28 post-onset	decreased (-0.914984796 fold)	regulate inflammation, immune responses, and angiogenesis	Diagnostic Biomarker
**467a-3p**	brain tissue (Day 1 data)	animal	30	collected days 1/3/7/14/28 post-onset	decreased (-0.945976991 fold)	regulate inflammation, immune responses, and angiogenesis	Diagnostic Biomarker
**467d-3p**	brain tissue (Day 1 data)	animal	30	collected days 1/3/7/14/28 post-onset	decreased (-0.945976991 fold)	regulate inflammation, immune responses, and angiogenesis	Diagnostic Biomarker
**671-5p**	brain tissue (Day 1 data)	animal	30	collected days 1/3/7/14/28 post-onset	decreased (-0.998752343 fold)	regulate inflammation, immune responses, and angiogenesis	Diagnostic Biomarker
**328-3p**	brain tissue (Day 1 data)	animal	30	collected days 1/3/7/14/28 post-onset	decreased (-1.029849561 fold)	regulate inflammation, immune responses, and angiogenesis	Diagnostic Biomarker
**501-5p**	brain tissue (Day 1 data)	animal	30	collected days 1/3/7/14/28 post-onset	decreased (-1.094546228 fold)	regulate inflammation, immune responses, and angiogenesis	Diagnostic Biomarker
	brain tissue (Day 28 data)	animal	30	collected days 1/3/7/14/28 post-onset	decreased (-1.327148068 fold)	regulate inflammation, immune responses, and angiogenesis	Diagnostic Biomarker
**let-7b-3p**	brain tissue (Day 1 data)	animal	30	collected days 1/3/7/14/28 post-onset	decreased (-1.162852619 fold)	regulate inflammation, immune responses, and angiogenesis	Diagnostic Biomarker
**669a-3p**	brain tissue (Day 1 data)	animal	30	collected days 1/3/7/14/28 post-onset	decreased (-1.188965553 fold)	regulate inflammation, immune responses, and angiogenesis	Diagnostic Biomarker
	brain tissue (Day 7 data)	animal	30	collected days 1/3/7/14/28 post-onset	decreased (-1.166326509 fold)	regulate inflammation, immune responses, and angiogenesis	Diagnostic Biomarker

**669o-3p**		brain tissue (Day 1 data)	animal	30	collected days 1/3/7/14/28 post-onset	decreased (-1.188965553 fold)	regulate inflammation, immune responses, and angiogenesis	Diagnostic Biomarker
		brain tissue (Day 7 data)	animal	30	collected days 1/3/7/14/28 post-onset	decreased (-1.166326509 fold)	regulate inflammation, immune responses, and angiogenesis	Diagnostic Biomarker
**433-3p**		brain tissue (Day 1 data)	animal	30	collected days 1/3/7/14/28 post-onset	decreased (-1.191416825 fold)	regulate inflammation, immune responses, and angiogenesis	Diagnostic Biomarker
		brain tissue (Day 14 data)	animal	30	collected days 1/3/7/14/28 post-onset	decreased (-0.761379328 fold)	regulate inflammation, immune responses, and angiogenesis	Diagnostic Biomarker
**3059-5p**		brain tissue (Day 1 data)	animal	30	collected days 1/3/7/14/28 post-onset	decreased (-1.196663433 fold)	regulate inflammation, immune responses, and angiogenesis	Diagnostic Biomarker
**29b-2-5p**		brain tissue (Day 1 data)	animal	30	collected days 1/3/7/14/28 post-onset	decreased (-1.216278172 fold)	regulate inflammation, immune responses, and angiogenesis	Diagnostic Biomarker
		brain tissue (Day 7 data)	animal	30	collected days 1/3/7/14/28 post-onset	decreased (-0.935461748 fold)	regulate inflammation, immune responses, and angiogenesis	Diagnostic Biomarker
**139-3p**		brain tissue (Day 1 data)	animal	30	collected days 1/3/7/14/28 post-onset	decreased (-1.28706174 fold)	regulate inflammation, immune responses, and angiogenesis	Diagnostic Biomarker
		brain tissue (Day 7 data)	animal	30	collected days 1/3/7/14/28 post-onset	decreased (-0.951901104 fold)	regulate inflammation, immune responses, and angiogenesis	Diagnostic Biomarker
		brain tissue (Day 14 data)	animal	30	collected days 1/3/7/14/28 post-onset	decreased (-0.73406249 fold)	regulate inflammation, immune responses, and angiogenesis	Diagnostic Biomarker
		brain tissue (Day 28 data)	animal	30	collected days 1/3/7/14/28 post-onset	decreased (-0.749037787 fold)	regulate inflammation, immune responses, and angiogenesis	Diagnostic Biomarker
**let-7e-3p**		brain tissue (Day 1 data)	animal	30	collected days 1/3/7/14/28 post-onset	decreased (-1.299376387 fold)	regulate inflammation, immune responses, and angiogenesis	Diagnostic Biomarker
**1982-3p**		brain tissue (Day 1 data)	animal	30	collected days 1/3/7/14/28 post-onset	decreased (-1.315340227 fold)	regulate inflammation, immune responses, and angiogenesis	Diagnostic Biomarker
**1306-5p**		brain tissue (Day 1 data)	animal	30	collected days 1/3/7/14/28 post-onset	decreased (-1.326184842 fold)	regulate inflammation, immune responses, and angiogenesis	Diagnostic Biomarker
		brain tissue (Day 3 data)	animal	30	collected days 1/3/7/14/28 post-onset	decreased (-1.425748687 fold)	regulate inflammation, immune responses, and angiogenesis	Diagnostic Biomarker
**181a-2-3p**		brain tissue (Day 1 data)	animal	30	collected days 1/3/7/14/28 post-onset	decreased (-1.354211997 fold)	regulate inflammation, immune responses, and angiogenesis	Diagnostic Biomarker
		brain tissue (Day 14 data)	animal	30	collected days 1/3/7/14/28 post-onset	increased (0.79364418 fold)	regulate synapses, cognition, axonogenesis, and ion transmembrane transport	Diagnostic Biomarker
		brain tissue (Day 28 data)	animal	30	collected days 1/3/7/14/28 post-onset	increased (1.335543 fold)	regulate synapses, cognition, axonogenesis, and ion transmembrane transport	Diagnostic Biomarker

**let-7a-2-3p**	brain tissue (Day 1 data)	animal	30	collected days 1/3/7/14/28 post-onset	decreased (-1.362555763 fold)	regulate inflammation, immune responses, and angiogenesis	Diagnostic Biomarker
**150-3p**	brain tissue (Day 1 data)	animal	30	collected days 1/3/7/14/28 post-onset	decreased (-1.653258585 fold)	regulate inflammation, immune responses, and angiogenesis	Diagnostic Biomarker
**1249-3p**	brain tissue (Day 1 data)	animal	30	collected days 1/3/7/14/28 post-onset	decreased (-1.656585524 fold)	regulate inflammation, immune responses, and angiogenesis	Diagnostic Biomarker
**34b-3p**	brain tissue (Day 1 data)	animal	30	collected days 1/3/7/14/28 post-onset	decreased (-1.701672218 fold)	regulate inflammation, immune responses, and angiogenesis	Diagnostic Biomarker
	brain tissue (Day 7 data)	animal	30	collected days 1/3/7/14/28 post-onset	increased (0.874860531 fold)	regulate synapses, cognition, axonogenesis, and ion transmembrane transport	Diagnostic Biomarker
	brain tissue (Day 14 data)	animal	30	collected days 1/3/7/14/28 post-onset	increased (0.671481002 fold)	regulate synapses, cognition, axonogenesis, and ion transmembrane transport	Diagnostic Biomarker
**326-3p**	brain tissue (Day 1 data)	animal	30	collected days 1/3/7/14/28 post-onset	decreased (-1.805043207 fold)	regulate inflammation, immune responses, and angiogenesis	Diagnostic Biomarker
**6962-3p**	brain tissue (Day 1 data)	animal	30	collected days 1/3/7/14/28 post-onset	decreased (-2.275789189 fold)	regulate inflammation, immune responses, and angiogenesis	Diagnostic Biomarker
	brain tissue (Day 3 data)	animal	30	collected days 1/3/7/14/28 post-onset	decreased (-2.569171577 fold)	regulate inflammation, immune responses, and angiogenesis	Diagnostic Biomarker
**12191-3p**	brain tissue (Day 1 data)	animal	30	collected days 1/3/7/14/28 post-onset	decreased (-2.340735981 fold)	regulate inflammation, immune responses, and angiogenesis	Diagnostic Biomarker
	brain tissue (Day 3 data)	animal	30	collected days 1/3/7/14/28 post-onset	decreased (-2.182983999 fold)	regulate inflammation, immune responses, and angiogenesis	Diagnostic Biomarker
**6982-3p**	brain tissue (Day 1 data)	animal	30	collected days 1/3/7/14/28 post-onset	decreased (-2.844894713 fold)	regulate inflammation, immune responses, and angiogenesis	Diagnostic Biomarker
**1970c-3p**	brain tissue (Day 1 data)	animal	30	collected days 1/3/7/14/28 post-onset	decreased (-4.172212683 fold)	regulate inflammation, immune responses, and angiogenesis	Diagnostic Biomarker
	brain tissue (Day 7 data)	animal	30	collected days 1/3/7/14/28 post-onset	decreased (-5.703837546 fold)	regulate inflammation, immune responses, and angiogenesis	Diagnostic Biomarker
**669b-5p**	brain tissue (Day 1 data)	animal	30	collected days 1/3/7/14/28 post-onset	decreased (-4.433984804 fold)	regulate inflammation, immune responses, and angiogenesis	Diagnostic Biomarker
**6920-3p**	brain tissue (Day 1 data)	animal	30	collected days 1/3/7/14/28 post-onset	decreased (-4.460169754 fold)	regulate inflammation, immune responses, and angiogenesis	Diagnostic Biomarker
**20b-5p**	brain tissue (Day 1 data)	animal	30	collected days 1/3/7/14/28 post-onset	decreased (-4.503481889 fold)	regulate inflammation, immune responses, and angiogenesis	Diagnostic Biomarker
**5122**	brain tissue (Day 1 data)	animal	30	collected days 1/3/7/14/28 post-onset	decreased (-4.700758282 fold)	regulate inflammation, immune responses, and angiogenesis	Diagnostic Biomarker

		brain tissue (Day 28 data)	animal	30	collected days 1/3/7/14/28 post-onset	increased (2.795493933 fold)	regulate synapses, cognition, axonogenesis, and ion transmembrane transport	Diagnostic Biomarker
**1943-3p**		brain tissue (Day 1 data)	animal	30	collected days 1/3/7/14/28 post-onset	decreased (-4.776608301 fold)	regulate inflammation, immune responses, and angiogenesis	Diagnostic Biomarker
**6481**		brain tissue (Day 3 data)	animal	30	collected days 1/3/7/14/28 post-onset	increased (7.725744531 fold)	regulate synapses, cognition, axonogenesis, and ion transmembrane transport	Diagnostic Biomarker
**6539**		brain tissue (Day 3 data)	animal	30	collected days 1/3/7/14/28 post-onset	increased (6.294388937 fold)	regulate synapses, cognition, axonogenesis, and ion transmembrane transport	Diagnostic Biomarker
**497a-3p**		brain tissue (Day 3 data)	animal	30	collected days 1/3/7/14/28 post-onset	increased (5.68529454 fold)	regulate synapses, cognition, axonogenesis, and ion transmembrane transport	Diagnostic Biomarker
**6537-3p**		brain tissue (Day 3 data)	animal	30	collected days 1/3/7/14/28 post-onset	increased (5.109811618 fold)	regulate synapses, cognition, axonogenesis, and ion transmembrane transport	Diagnostic Biomarker
**12206-5p**		brain tissue (Day 3 data)	animal	30	collected days 1/3/7/14/28 post-onset	increased (5.103316388 fold)	regulate synapses, cognition, axonogenesis, and ion transmembrane transport	Diagnostic Biomarker
**141-5p**		brain tissue (Day 3 data)	animal	30	collected days 1/3/7/14/28 post-onset	increased (4.248386424 fold)	regulate synapses, cognition, axonogenesis, and ion transmembrane transport	Diagnostic Biomarker
**876-5p**		brain tissue (Day 3 data)	animal	30	collected days 1/3/7/14/28 post-onset	increased (3.763552601 fold)	regulate synapses, cognition, axonogenesis, and ion transmembrane transport	Diagnostic Biomarker
**32-5p**		brain tissue (Day 3 data)	animal	30	collected days 1/3/7/14/28 post-onset	increased (3.506284451 fold)	regulate synapses, cognition, axonogenesis, and ion transmembrane transport	Diagnostic Biomarker
**503-5p**		brain tissue (Day 3 data)	animal	30	collected days 1/3/7/14/28 post-onset	increased (3.377036218 fold)	regulate synapses, cognition, axonogenesis, and ion transmembrane transport	Diagnostic Biomarker
**429-3p**		brain tissue (Day 3 data)	animal	30	collected days 1/3/7/14/28 post-onset	increased (3.227036173 fold)	regulate synapses, cognition, axonogenesis, and ion transmembrane transport	Diagnostic Biomarker
		brain tissue (Day 7 data)	animal	30	collected days 1/3/7/14/28 post-onset	increased (1.315963046 fold)	regulate synapses, cognition, axonogenesis, and ion transmembrane transport	Diagnostic Biomarker
		brain tissue (Day 28 data)	animal	30	collected days 1/3/7/14/28 post-onset	increased (1.447421844 fold)	regulate synapses, cognition, axonogenesis, and ion transmembrane transport	Diagnostic Biomarker
**208b-3p**		brain tissue (Day 3 data)	animal	30	collected days 1/3/7/14/28 post-onset	increased (2.902074647 fold)	regulate synapses, cognition, axonogenesis, and ion transmembrane transport	Diagnostic Biomarker
**182-5p**		brain tissue (Day 3 data)	animal	30	collected days 1/3/7/14/28 post-onset	increased (2.565791484 fold)	regulate synapses, cognition, axonogenesis, and ion transmembrane transport	Diagnostic Biomarker
		brain tissue (Day 7 data)	animal	30	collected days 1/3/7/14/28 post-onset	increased(3.5823544 fold)	regulate synapses, cognition, axonogenesis, and ion transmembrane transport	Diagnostic Biomarker
**200c-3p**		brain tissue (Day 3 data)	animal	30	collected days 1/3/7/14/28 post-onset	increased (2.470820265 fold)	regulate synapses, cognition, axonogenesis, and ion transmembrane transport	Diagnostic Biomarker

	brain tissue (Day 7 data)	animal	30	collected days 1/3/7/14/28 post-onset	increased (3.813045972 fold)	regulate synapses, cognition, axonogenesis, and ion transmembrane transport	Diagnostic Biomarker
	brain tissue (Day 28 data)	animal	30	collected days 1/3/7/14/28 post-onset	increased (2.212339352 fold)	regulate synapses, cognition, axonogenesis, and ion transmembrane transport	Diagnostic Biomarker
**362-3p**	brain tissue (Day 3 data)	animal	30	collected days 1/3/7/14/28 post-onset	increased (2.370682295 fold)	regulate synapses, cognition, axonogenesis, and ion transmembrane transport	Diagnostic Biomarker
**190a-5p**	brain tissue (Day 3 data)	animal	30	collected days 1/3/7/14/28 post-onset	increased (2.338587194 fold)	regulate synapses, cognition, axonogenesis, and ion transmembrane transport	Diagnostic Biomarker
**183-3p**	brain tissue (Day 3 data)	animal	30	collected days 1/3/7/14/28 post-onset	increased (2.338158594 fold)	regulate synapses, cognition, axonogenesis, and ion transmembrane transport	Diagnostic Biomarker
**200a-5p**	brain tissue (Day 3 data)	animal	30	collected days 1/3/7/14/28 post-onset	increased (2.332503775 fold)	regulate synapses, cognition, axonogenesis, and ion transmembrane transport	Diagnostic Biomarker
	brain tissue (Day 7 data)	animal	30	collected days 1/3/7/14/28 post-onset	increased (2.697597302 fold)	regulate synapses, cognition, axonogenesis, and ion transmembrane transport	Diagnostic Biomarker
**15b-3p**	brain tissue (Day 3 data)	animal	30	collected days 1/3/7/14/28 post-onset	increased (2.150608973 fold)	regulate synapses, cognition, axonogenesis, and ion transmembrane transport	Diagnostic Biomarker
	brain tissue (Day 7 data)	animal	30	collected days 1/3/7/14/28 post-onset	increased (2.288187243 fold)	regulate synapses, cognition, axonogenesis, and ion transmembrane transport	Diagnostic Biomarker
	brain tissue (Day 14 data)	animal	30	collected days 1/3/7/14/28 post-onset	increased (1.336646092 fold)	regulate synapses, cognition, axonogenesis, and ion transmembrane transport	Diagnostic Biomarker
**542-3p**	brain tissue (Day 3 data)	animal	30	collected days 1/3/7/14/28 post-onset	increased (2.141082304 fold)	regulate synapses, cognition, axonogenesis, and ion transmembrane transport	Diagnostic Biomarker
**300-5p**	brain tissue (Day 3 data)	animal	30	collected days 1/3/7/14/28 post-onset	increased (2.069230326 fold)	regulate synapses, cognition, axonogenesis, and ion transmembrane transport	Diagnostic Biomarker
**544-5p**	brain tissue (Day 3 data)	animal	30	collected days 1/3/7/14/28 post-onset	increased (2.039932322 fold)	regulate synapses, cognition, axonogenesis, and ion transmembrane transport	Diagnostic Biomarker
**301a-3p**	brain tissue (Day 3 data)	animal	30	collected days 1/3/7/14/28 post-onset	increased (2.023084518 fold)	regulate synapses, cognition, axonogenesis, and ion transmembrane transport	Diagnostic Biomarker
	brain tissue (Day 14 data)	animal	30	collected days 1/3/7/14/28 post-onset	increased (0.464751991 fold)	regulate synapses, cognition, axonogenesis, and ion transmembrane transport	Diagnostic Biomarker
**let-7f-2-3p**	brain tissue (Day 3 data)	animal	30	collected days 1/3/7/14/28 post-onset	increased (1.98641243 fold)	regulate synapses, cognition, axonogenesis, and ion transmembrane transport	Diagnostic Biomarker
**29b-3p**	brain tissue (Day 3 data)	animal	30	collected days 1/3/7/14/28 post-onset	increased (1.867498432 fold)	regulate synapses, cognition, axonogenesis, and ion transmembrane transport	Diagnostic Biomarker
**30e-5p**	brain tissue (Day 3 data)	animal	30	collected days 1/3/7/14/28 post-onset	increased (1.735100062 fold)	regulate synapses, cognition, axonogenesis, and ion transmembrane transport	Diagnostic Biomarker

**let-7a-1-3p**	brain tissue (Day 3 data)	animal	30	collected days 1/3/7/14/28 post-onset	increased (1.6695754 fold)	regulate synapses, cognition, axonogenesis, and ion transmembrane transport	Diagnostic Biomarker
**let-7c-2-3p**	brain tissue (Day 3 data)	animal	30	collected days 1/3/7/14/28 post-onset	increased (1.6695754 fold)	regulate synapses, cognition, axonogenesis, and ion transmembrane transport	Diagnostic Biomarker
**3968**	brain tissue (Day 3 data)	animal	30	collected days 1/3/7/14/28 post-onset	increased (1.625369344 fold)	regulate synapses, cognition, axonogenesis, and ion transmembrane transport	Diagnostic Biomarker
	brain tissue (Day 28 data)	animal	30	collected days 1/3/7/14/28 post-onset	increased (0.997317884 fold)	regulate synapses, cognition, axonogenesis, and ion transmembrane transport	Diagnostic Biomarker
**34b-5p**	brain tissue (Day 3 data)	animal	30	collected days 1/3/7/14/28 post-onset	increased (1.58770699 fold)	regulate synapses, cognition, axonogenesis, and ion transmembrane transport	Diagnostic Biomarker
	brain tissue (Day 7 data)	animal	30	collected days 1/3/7/14/28 post-onset	increased (1.62701082 fold)	regulate synapses, cognition, axonogenesis, and ion transmembrane transport	Diagnostic Biomarker
**7a-5p**	brain tissue (Day 3 data)	animal	30	collected days 1/3/7/14/28 post-onset	increased (1.586500984 fold)	regulate synapses, cognition, axonogenesis, and ion transmembrane transport	Diagnostic Biomarker
**101a-3p**	brain tissue (Day 3 data)	animal	30	collected days 1/3/7/14/28 post-onset	increased (1.558231349 fold)	regulate synapses, cognition, axonogenesis, and ion transmembrane transport	Diagnostic Biomarker
**101c**	brain tissue (Day 3 data)	animal	30	collected days 1/3/7/14/28 post-onset	increased (1.557842002 fold)	regulate synapses, cognition, axonogenesis, and ion transmembrane transport	Diagnostic Biomarker
**101b-3p**	brain tissue (Day 3 data)	animal	30	collected days 1/3/7/14/28 post-onset	increased (1.543573762 fold)	regulate synapses, cognition, axonogenesis, and ion transmembrane transport	Diagnostic Biomarker
**136-3p**	brain tissue (Day 3 data)	animal	30	collected days 1/3/7/14/28 post-onset	increased (1.501436196 fold)	regulate synapses, cognition, axonogenesis, and ion transmembrane transport	Diagnostic Biomarker
**380-3p**	brain tissue (Day 3 data)	animal	30	collected days 1/3/7/14/28 post-onset	increased (1.492327417 fold)	regulate synapses, cognition, axonogenesis, and ion transmembrane transport	Diagnostic Biomarker
**376c-3p**	brain tissue (Day 3 data)	animal	30	collected days 1/3/7/14/28 post-onset	increased (1.474112272 fold)	regulate synapses, cognition, axonogenesis, and ion transmembrane transport	Diagnostic Biomarker
	brain tissue (Day 28 data)	animal	30	collected days 1/3/7/14/28 post-onset	decreased (-0.816338811 fold)	regulate inflammation, immune responses, and angiogenesis	Diagnostic Biomarker
**29c-3p**	brain tissue (Day 3 data)	animal	30	collected days 1/3/7/14/28 post-onset	increased (1.436245264 fold)	regulate synapses, cognition, axonogenesis, and ion transmembrane transport	Diagnostic Biomarker
**29a-5p**	brain tissue (Day 3 data)	animal	30	collected days 1/3/7/14/28 post-onset	increased (1.390213878 fold)	regulate synapses, cognition, axonogenesis, and ion transmembrane transport	Diagnostic Biomarker
**384-3p**	brain tissue (Day 3 data)	animal	30	collected days 1/3/7/14/28 post-onset	increased (1.365259861 fold)	regulate synapses, cognition, axonogenesis, and ion transmembrane transport	Diagnostic Biomarker
**380-5p**	brain tissue (Day 3 data)	animal	30	collected days 1/3/7/14/28 post-onset	increased (1.360769528 fold)	regulate synapses, cognition, axonogenesis, and ion transmembrane transport	Diagnostic Biomarker

**219a-5p**	brain tissue (Day 3 data)	animal	30	collected days 1/3/7/14/28 post-onset	increased (1.337775543 fold)	regulate synapses, cognition, axonogenesis, and ion transmembrane transport	Diagnostic Biomarker
	brain tissue (Day 7 data)	animal	30	collected days 1/3/7/14/28 post-onset	increased (1.277696792 fold)	regulate synapses, cognition, axonogenesis, and ion transmembrane transport	Diagnostic Biomarker
	brain tissue (Day 28 data)	animal	30	collected days 1/3/7/14/28 post-onset	decreased (-1.102553316 fold)	regulate inflammation, immune responses, and angiogenesis	Diagnostic Biomarker
**7b-3p**	brain tissue (Day 3 data)	animal	30	collected days 1/3/7/14/28 post-onset	increased (1.328140229 fold)	regulate synapses, cognition, axonogenesis, and ion transmembrane transport	Diagnostic Biomarker
**137-3p**	brain tissue (Day 3 data)	animal	30	collected days 1/3/7/14/28 post-onset	increased (1.318894138 fold)	regulate synapses, cognition, axonogenesis, and ion transmembrane transport	Diagnostic Biomarker
**140-5p**	brain tissue (Day 3 data)	animal	30	collected days 1/3/7/14/28 post-onset	increased (1.2958596 fold)	regulate synapses, cognition, axonogenesis, and ion transmembrane transport	Diagnostic Biomarker
**540-5p**	brain tissue (Day 3 data)	animal	30	collected days 1/3/7/14/28 post-onset	increased (1.201188351 fold)	regulate synapses, cognition, axonogenesis, and ion transmembrane transport	Diagnostic Biomarker
**301b-3p**	brain tissue (Day 3 data)	animal	30	collected days 1/3/7/14/28 post-onset	increased (1.151560668 fold)	regulate synapses, cognition, axonogenesis, and ion transmembrane transport	Diagnostic Biomarker
	brain tissue (Day 7 data)	animal	30	collected days 1/3/7/14/28 post-onset	increased (1.0678271 fold)	regulate synapses, cognition, axonogenesis, and ion transmembrane transport	Diagnostic Biomarker
**673-5p**	brain tissue (Day 3 data)	animal	30	collected days 1/3/7/14/28 post-onset	decreased (-1.094147335 fold)	regulate inflammation, immune responses, and angiogenesis	Diagnostic Biomarker
	brain tissue (Day 7 data)	animal	30	collected days 1/3/7/14/28 post-onset	decreased (-1.4128878 fold)	regulate inflammation, immune responses, and angiogenesis	Diagnostic Biomarker
**871-3p**	brain tissue (Day 3 data)	animal	30	collected days 1/3/7/14/28 post-onset	decreased (-2.086306521 fold)	regulate inflammation, immune responses, and angiogenesis	Diagnostic Biomarker
**466h-3p**	brain tissue (Day 3 data)	animal	30	collected days 1/3/7/14/28 post-onset	decreased (-2.581088526 fold)	regulate inflammation, immune responses, and angiogenesis	Diagnostic Biomarker
**5617-3p**	brain tissue (Day 3 data)	animal	30	collected days 1/3/7/14/28 post-onset	decreased (-2.958450737 fold)	regulate inflammation, immune responses, and angiogenesis	Diagnostic Biomarker
**1193-5p**	brain tissue (Day 3 data)	animal	30	collected days 1/3/7/14/28 post-onset	decreased (-3.847206074 fold)	regulate inflammation, immune responses, and angiogenesis	Diagnostic Biomarker
**504-3p**	brain tissue (Day 3 data)	animal	30	collected days 1/3/7/14/28 post-onset	decreased (-4.201639471 fold)	regulate inflammation, immune responses, and angiogenesis	Diagnostic Biomarker
	brain tissue (Day 14 data)	animal	30	collected days 1/3/7/14/28 post-onset	decreased (-3.94547767 fold)	regulate inflammation, immune responses, and angiogenesis	Diagnostic Biomarker
**5620-5p**	brain tissue (Day 3 data)	animal	30	collected days 1/3/7/14/28 post-onset	decreased (-4.620715474 fold)	regulate inflammation, immune responses, and angiogenesis	Diagnostic Biomarker

	brain tissue (Day 7 data)	animal	30	collected days 1/3/7/14/28 post-onset	decreased (-4.631850796 fold)	regulate inflammation, immune responses, and angiogenesis	Diagnostic Biomarker
**365-2-5p**	brain tissue (Day 7 data)	animal	30	collected days 1/3/7/14/28 post-onset	increased (5.800532723 fold)	regulate synapses, cognition, axonogenesis, and ion transmembrane transport	Diagnostic Biomarker
**326-5p**	brain tissue (Day 7 data)	animal	30	collected days 1/3/7/14/28 post-onset	increased (5.505798746 fold)	regulate synapses, cognition, axonogenesis, and ion transmembrane transport	Diagnostic Biomarker
**219c-3p**	brain tissue (Day 7 data)	animal	30	collected days 1/3/7/14/28 post-onset	increased (5.071822409 fold)	regulate synapses, cognition, axonogenesis, and ion transmembrane transport	Diagnostic Biomarker
**5100**	brain tissue (Day 7 data)	animal	30	collected days 1/3/7/14/28 post-onset	increased (4.58870291 fold)	regulate synapses, cognition, axonogenesis, and ion transmembrane transport	Diagnostic Biomarker
**7669-3p**	brain tissue (Day 7 data)	animal	30	collected days 1/3/7/14/28 post-onset	increased (4.466529361 fold)	regulate synapses, cognition, axonogenesis, and ion transmembrane transport	Diagnostic Biomarker
**142a-3p**	brain tissue (Day 7 data)	animal	30	collected days 1/3/7/14/28 post-onset	increased (4.167943149 fold)	regulate synapses, cognition, axonogenesis, and ion transmembrane transport	Diagnostic Biomarker
	brain tissue (Day 14 data)	animal	30	collected days 1/3/7/14/28 post-onset	increased (3.646440019 fold)	regulate synapses, cognition, axonogenesis, and ion transmembrane transport	Diagnostic Biomarker
	brain tissue (Day 28 data)	animal	30	collected days 1/3/7/14/28 post-onset	increased (2.555179049 fold)	regulate synapses, cognition, axonogenesis, and ion transmembrane transport	Diagnostic Biomarker
**16-2-3p**	brain tissue (Day 7 data)	animal	30	collected days 1/3/7/14/28 post-onset	increased (4.112532337 fold)	regulate synapses, cognition, axonogenesis, and ion transmembrane transport	Diagnostic Biomarker
**214-5p**	brain tissue (Day 7 data)	animal	30	collected days 1/3/7/14/28 post-onset	increased (3.814225992 fold)	regulate synapses, cognition, axonogenesis, and ion transmembrane transport	Diagnostic Biomarker
**32-3p**	brain tissue (Day 7 data)	animal	30	collected days 1/3/7/14/28 post-onset	increased (3.397238809 fold)	regulate synapses, cognition, axonogenesis, and ion transmembrane transport	Diagnostic Biomarker
**214-3p**	brain tissue (Day 7 data)	animal	30	collected days 1/3/7/14/28 post-onset	increased (3.396387987 fold)	regulate synapses, cognition, axonogenesis, and ion transmembrane transport	Diagnostic Biomarker
	brain tissue (Day 14 data)	animal	30	collected days 1/3/7/14/28 post-onset	increased (3.770372058 fold)	regulate synapses, cognition, axonogenesis, and ion transmembrane transport	Diagnostic Biomarker
	brain tissue (Day 28 data)	animal	30	collected days 1/3/7/14/28 post-onset	increased (3.404672348 fold)	regulate synapses, cognition, axonogenesis, and ion transmembrane transport	Diagnostic Biomarker
**199a-3p**	brain tissue (Day 7 data)	animal	30	collected days 1/3/7/14/28 post-onset	increased (3.256863572 fold)	regulate synapses, cognition, axonogenesis, and ion transmembrane transport	Diagnostic Biomarker
	brain tissue (Day 14 data)	animal	30	collected days 1/3/7/14/28 post-onset	increased (1.934066954 fold)	regulate synapses, cognition, axonogenesis, and ion transmembrane transport	Diagnostic Biomarker
	brain tissue (Day 28 data)	animal	30	collected days 1/3/7/14/28 post-onset	increased (1.084849708 fold)	regulate synapses, cognition, axonogenesis, and ion transmembrane transport	Diagnostic Biomarker

**199b-3p**	brain tissue (Day 7 data)	animal	30	collected days 1/3/7/14/28 post-onset	increased (3.256863572 fold)	regulate synapses, cognition, axonogenesis, and ion transmembrane transport	Diagnostic Biomarker
	brain tissue (Day 14 data)	animal	30	collected days 1/3/7/14/28 post-onset	increased (1.934066954 fold)	regulate synapses, cognition, axonogenesis, and ion transmembrane transport	Diagnostic Biomarker
	brain tissue (Day 28 data)	animal	30	collected days 1/3/7/14/28 post-onset	increased (1.084849708 fold)	regulate synapses, cognition, axonogenesis, and ion transmembrane transport	Diagnostic Biomarker
**7083-5p**	brain tissue (Day 7 data)	animal	30	collected days 1/3/7/14/28 post-onset	increased (2.933135222 fold)	regulate synapses, cognition, axonogenesis, and ion transmembrane transport	Diagnostic Biomarker
**702-5p**	brain tissue (Day 7 data)	animal	30	collected days 1/3/7/14/28 post-onset	increased (2.407018175 fold)	regulate synapses, cognition, axonogenesis, and ion transmembrane transport	Diagnostic Biomarker
**410-5p**	brain tissue (Day 7 data)	animal	30	collected days 1/3/7/14/28 post-onset	increased (2.203763864 fold)	regulate synapses, cognition, axonogenesis, and ion transmembrane transport	Diagnostic Biomarker
**1199-5p**	brain tissue (Day 7 data)	animal	30	collected days 1/3/7/14/28 post-onset	increased (2.003381801 fold)	regulate synapses, cognition, axonogenesis, and ion transmembrane transport	Diagnostic Biomarker
	brain tissue (Day 14 data)	animal	30	collected days 1/3/7/14/28 post-onset	increased (3.796706029 fold)	regulate synapses, cognition, axonogenesis, and ion transmembrane transport	Diagnostic Biomarker
	brain tissue (Day 28 data)	animal	30	collected days 1/3/7/14/28 post-onset	increased (3.743904693 fold)	regulate synapses, cognition, axonogenesis, and ion transmembrane transport	Diagnostic Biomarker
**450b-5p**	brain tissue (Day 7 data)	animal	30	collected days 1/3/7/14/28 post-onset	increased (1.685175922 fold)	regulate synapses, cognition, axonogenesis, and ion transmembrane transport	Diagnostic Biomarker
**296-3p**	brain tissue (Day 7 data)	animal	30	collected days 1/3/7/14/28 post-onset	increased (1.567263734 fold)	regulate synapses, cognition, axonogenesis, and ion transmembrane transport	Diagnostic Biomarker
	brain tissue (Day 14 data)	animal	30	collected days 1/3/7/14/28 post-onset	increased (1.032884172 fold)	regulate synapses, cognition, axonogenesis, and ion transmembrane transport	Diagnostic Biomarker
**152-3p**	brain tissue (Day 7 data)	animal	30	collected days 1/3/7/14/28 post-onset	increased (1.452834839 fold)	regulate synapses, cognition, axonogenesis, and ion transmembrane transport	Diagnostic Biomarker
	brain tissue (Day 14 data)	animal	30	collected days 1/3/7/14/28 post-onset	increased (1.164102485 fold)	regulate synapses, cognition, axonogenesis, and ion transmembrane transport	Diagnostic Biomarker
**669a-5p**	brain tissue (Day 7 data)	animal	30	collected days 1/3/7/14/28 post-onset	increased (1.358222496 fold)	regulate synapses, cognition, axonogenesis, and ion transmembrane transport	Diagnostic Biomarker
**669p-5p**	brain tissue (Day 7 data)	animal	30	collected days 1/3/7/14/28 post-onset	increased (1.358222496 fold)	regulate synapses, cognition, axonogenesis, and ion transmembrane transport	Diagnostic Biomarker
**8112**	brain tissue (Day 7 data)	animal	30	collected days 1/3/7/14/28 post-onset	increased (1.353718807 fold)	regulate synapses, cognition, axonogenesis, and ion transmembrane transport	Diagnostic Biomarker
	brain tissue (Day 14 data)	animal	30	collected days 1/3/7/14/28 post-onset	increased (0.706983733 fold)	regulate synapses, cognition, axonogenesis, and ion transmembrane transport	Diagnostic Biomarker

	brain tissue (Day 28 data)	animal	30	collected days 1/3/7/14/28 post-onset	increased (1.310757966 fold)	regulate synapses, cognition, axonogenesis, and ion transmembrane transport	Diagnostic Biomarker
**34c-5p**	brain tissue (Day 7 data)	animal	30	collected days 1/3/7/14/28 post-onset	increased(1.195129913 fold)	regulate synapses, cognition, axonogenesis, and ion transmembrane transport	Diagnostic Biomarker
	brain tissue (Day 14 data)	animal	30	collected days 1/3/7/14/28 post-onset	increased (1.498479614 fold)	regulate synapses, cognition, axonogenesis, and ion transmembrane transport	Diagnostic Biomarker
**25-3p**	brain tissue (Day 7 data)	animal	30	collected days 1/3/7/14/28 post-onset	increased (1.101737152 fold)	regulate synapses, cognition, axonogenesis, and ion transmembrane transport	Diagnostic Biomarker
**34c-3p**	brain tissue (Day 7 data)	animal	30	collected days 1/3/7/14/28 post-onset	increased (1.098547271 fold)	regulate synapses, cognition, axonogenesis, and ion transmembrane transport	Diagnostic Biomarker
**152-5p**	brain tissue (Day 7 data)	animal	30	collected days 1/3/7/14/28 post-onset	increased (1.01746973 fold)	regulate synapses, cognition, axonogenesis, and ion transmembrane transport	Diagnostic Biomarker
	brain tissue (Day 14 data)	animal	30	collected days 1/3/7/14/28 post-onset	increased (0.923853134 fold)	regulate synapses, cognition, axonogenesis, and ion transmembrane transport	Diagnostic Biomarker
**350-3p**	brain tissue (Day 7 data)	animal	30	collected days 1/3/7/14/28 post-onset	increased (0.936665397 fold)	regulate synapses, cognition, axonogenesis, and ion transmembrane transport	Diagnostic Biomarker
**130b-3p**	brain tissue (Day 7 data)	animal	30	collected days 1/3/7/14/28 post-onset	increased (0.901855076 fold)	regulate synapses, cognition, axonogenesis, and ion transmembrane transport	Diagnostic Biomarker
**7220-5p**	brain tissue (Day 7 data)	animal	30	collected days 1/3/7/14/28 post-onset	increased (0.894013968 fold)	regulate synapses, cognition, axonogenesis, and ion transmembrane transport	Diagnostic Biomarker
**15b-5p**	brain tissue (Day 7 data)	animal	30	collected days 1/3/7/14/28 post-onset	increased (0.889900994 fold)	regulate synapses, cognition, axonogenesis, and ion transmembrane transport	Diagnostic Biomarker
	brain tissue (Day 14 data)	animal	30	collected days 1/3/7/14/28 post-onset	increased (1.130929921 fold)	regulate synapses, cognition, axonogenesis, and ion transmembrane transport	Diagnostic Biomarker
	brain tissue (Day 28 data)	animal	30	collected days 1/3/7/14/28 post-onset	increased (1.409219993 fold)	regulate synapses, cognition, axonogenesis, and ion transmembrane transport	Diagnostic Biomarker
**322-5p**	brain tissue (Day 7 data)	animal	30	collected days 1/3/7/14/28 post-onset	increased (0.846017468 fold)	regulate synapses, cognition, axonogenesis, and ion transmembrane transport	Diagnostic Biomarker
	brain tissue (Day 14 data)	animal	30	collected days 1/3/7/14/28 post-onset	increased (0.74324827 fold)	regulate synapses, cognition, axonogenesis, and ion transmembrane transport	Diagnostic Biomarker
**28c**	brain tissue (Day 7 data)	animal	30	collected days 1/3/7/14/28 post-onset	increased (0.840165764 fold)	regulate synapses, cognition, axonogenesis, and ion transmembrane transport	Diagnostic Biomarker
**28a-5p**	brain tissue (Day 7 data)	animal	30	collected days 1/3/7/14/28 post-onset	increased (0.837193016 fold)	regulate synapses, cognition, axonogenesis, and ion transmembrane transport	Diagnostic Biomarker
**let-7j**	brain tissue (Day 7 data)	animal	30	collected days 1/3/7/14/28 post-onset	increased (0.742127141 fold)	regulate synapses, cognition, axonogenesis, and ion transmembrane transport	Diagnostic Biomarker

**330-5p**	brain tissue (Day 7 data)	animal	30	collected days 1/3/7/14/28 post-onset	decreased (-0.725271812 fold)	regulate inflammation, immune responses, and angiogenesis	Diagnostic Biomarker
**133a-3p**	brain tissue (Day 7 data)	animal	30	collected days 1/3/7/14/28 post-onset	decreased (-0.745791698 fold)	regulate inflammation, immune responses, and angiogenesis	Diagnostic Biomarker
**154-5p**	brain tissue (Day 7 data)	animal	30	collected days 1/3/7/14/28 post-onset	decreased (-0.776238623 fold)	regulate inflammation, immune responses, and angiogenesis	Diagnostic Biomarker
	brain tissue (Day 28 data)	animal	30	collected days 1/3/7/14/28 post-onset	decreased (-0.730665844 fold)	regulate inflammation, immune responses, and angiogenesis	Diagnostic Biomarker
**666-5p**	brain tissue (Day 7 data)	animal	30	collected days 1/3/7/14/28 post-onset	decreased (-0.802772786 fold)	regulate inflammation, immune responses, and angiogenesis	Diagnostic Biomarker
**29a-3p**	brain tissue (Day 7 data)	animal	30	collected days 1/3/7/14/28 post-onset	decreased (-0.816301185 fold)	regulate inflammation, immune responses, and angiogenesis	Diagnostic Biomarker
**410-3p**	brain tissue (Day 7 data)	animal	30	collected days 1/3/7/14/28 post-onset	decreased (-0.857815966 fold)	regulate inflammation, immune responses, and angiogenesis	Diagnostic Biomarker
**181c-5p**	brain tissue (Day 7 data)	animal	30	collected days 1/3/7/14/28 post-onset	decreased (-0.86474074 fold)	regulate inflammation, immune responses, and angiogenesis	Diagnostic Biomarker
**665-3p**	brain tissue (Day 7 data)	animal	30	collected days 1/3/7/14/28 post-onset	decreased (-0.895382337 fold)	regulate inflammation, immune responses, and angiogenesis	Diagnostic Biomarker
	brain tissue (Day 14 data)	animal	30	collected days 1/3/7/14/28 post-onset	decreased (-0.696548381 fold)	regulate inflammation, immune responses, and angiogenesis	Diagnostic Biomarker
	brain tissue (Day 28 data)	animal	30	collected days 1/3/7/14/28 post-onset	decreased (-0.886789058 fold)	regulate inflammation, immune responses, and angiogenesis	Diagnostic Biomarker
**1843b-3p**	brain tissue (Day 7 data)	animal	30	collected days 1/3/7/14/28 post-onset	decreased (-0.932764918 fold)	regulate inflammation, immune responses, and angiogenesis	Diagnostic Biomarker
	brain tissue (Day 14 data)	animal	30	collected days 1/3/7/14/28 post-onset	decreased (-0.564413446 fold)	regulate inflammation, immune responses, and angiogenesis	Diagnostic Biomarker
**409-5p**	brain tissue (Day 7 data)	animal	30	collected days 1/3/7/14/28 post-onset	decreased (-0.949062113 fold)	regulate inflammation, immune responses, and angiogenesis	Diagnostic Biomarker
**346-5p**	brain tissue (Day 7 data)	animal	30	collected days 1/3/7/14/28 post-onset	decreased (-0.952075682 fold)	regulate inflammation, immune responses, and angiogenesis	Diagnostic Biomarker
**329-3p**	brain tissue (Day 7 data)	animal	30	collected days 1/3/7/14/28 post-onset	decreased (-0.954479089 fold)	regulate inflammation, immune responses, and angiogenesis	Diagnostic Biomarker
**139-5p**	brain tissue (Day 7 data)	animal	30	collected days 1/3/7/14/28 post-onset	decreased (-0.9630055 fold)	regulate inflammation, immune responses, and angiogenesis	Diagnostic Biomarker
	brain tissue (Day 14 data)	animal	30	collected days 1/3/7/14/28 post-onset	decreased (-0.595584919 fold)	regulate inflammation, immune responses, and angiogenesis	Diagnostic Biomarker

**667-3p**	brain tissue (Day 7 data)	animal	30	collected days 1/3/7/14/28 post-onset	decreased (-1.062595041 fold)	regulate inflammation, immune responses, and angiogenesis	Diagnostic Biomarker
**668-3p**	brain tissue (Day 7 data)	animal	30	collected days 1/3/7/14/28 post-onset	decreased (-1.074084326 fold)	regulate inflammation, immune responses, and angiogenesis	Diagnostic Biomarker
	brain tissue (Day 14 data)	animal	30	collected days 1/3/7/14/28 post-onset	decreased (-0.593851702 fold)	regulate inflammation, immune responses, and angiogenesis	Diagnostic Biomarker
**344d-3p**	brain tissue (Day 7 data)	animal	30	collected days 1/3/7/14/28 post-onset	decreased (-1.087839454 fold)	regulate inflammation, immune responses, and angiogenesis	Diagnostic Biomarker
**127-3p**	brain tissue (Day 7 data)	animal	30	collected days 1/3/7/14/28 post-onset	decreased (-1.096305821 fold)	regulate inflammation, immune responses, and angiogenesis	Diagnostic Biomarker
**137-5p**	brain tissue (Day 7 data)	animal	30	collected days 1/3/7/14/28 post-onset	decreased (-1.113993518 fold)	regulate inflammation, immune responses, and angiogenesis	Diagnostic Biomarker
**671-3p**	brain tissue (Day 7 data)	animal	30	collected days 1/3/7/14/28 post-onset	decreased (-1.199687211 fold)	regulate inflammation, immune responses, and angiogenesis	Diagnostic Biomarker
**758-3p**	brain tissue (Day 7 data)	animal	30	collected days 1/3/7/14/28 post-onset	decreased (-1.213563397 fold)	regulate inflammation, immune responses, and angiogenesis	Diagnostic Biomarker
**323-5p**	brain tissue (Day 7 data)	animal	30	collected days 1/3/7/14/28 post-onset	decreased (-1.248957391 fold)	regulate inflammation, immune responses, and angiogenesis	Diagnostic Biomarker
**3102-3p**	brain tissue (Day 7 data)	animal	30	collected days 1/3/7/14/28 post-onset	decreased (-1.249790363 fold)	regulate inflammation, immune responses, and angiogenesis	Diagnostic Biomarker
**764-5p**	brain tissue (Day 7 data)	animal	30	collected days 1/3/7/14/28 post-onset	decreased (-1.30860474 fold)	regulate inflammation, immune responses, and angiogenesis	Diagnostic Biomarker
	brain tissue (Day 14 data)	animal	30	collected days 1/3/7/14/28 post-onset	decreased (-0.767232242 fold)	regulate inflammation, immune responses, and angiogenesis	Diagnostic Biomarker
**543-5p**	brain tissue (Day 7 data)	animal	30	collected days 1/3/7/14/28 post-onset	decreased (-1.431915775 fold)	regulate inflammation, immune responses, and angiogenesis	Diagnostic Biomarker
**365-3p**	brain tissue (Day 7 data)	animal	30	collected days 1/3/7/14/28 post-onset	decreased (-1.474815821 fold)	regulate inflammation, immune responses, and angiogenesis	Diagnostic Biomarker
**3572-3p**	brain tissue (Day 7 data)	animal	30	collected days 1/3/7/14/28 post-onset	decreased (-1.552910891 fold)	regulate inflammation, immune responses, and angiogenesis	Diagnostic Biomarker
**744-5p**	brain tissue (Day 7 data)	animal	30	collected days 1/3/7/14/28 post-onset	decreased (-1.5623474 fold)	regulate inflammation, immune responses, and angiogenesis	Diagnostic Biomarker
	brain tissue (Day 28 data)	animal	30	collected days 1/3/7/14/28 post-onset	increased (0.948004775 fold)	regulate synapses, cognition, axonogenesis, and ion transmembrane transport	Diagnostic Biomarker
**485-5p**	brain tissue (Day 7 data)	animal	30	collected days 1/3/7/14/28 post-onset	decreased (-1.589973407 fold)	regulate inflammation, immune responses, and angiogenesis	Diagnostic Biomarker

**377-5p**	brain tissue (Day 7 data)	animal	30	collected days 1/3/7/14/28 post-onset	decreased (-1.63428193 fold)	regulate inflammation, immune responses, and angiogenesis	Diagnostic Biomarker
	brain tissue (Day 14 data)	animal	30	collected days 1/3/7/14/28 post-onset	decreased (-1.017356086 fold)	regulate inflammation, immune responses, and angiogenesis	Diagnostic Biomarker
**193b-3p**	brain tissue (Day 7 data)	animal	30	collected days 1/3/7/14/28 post-onset	decreased (-1.63433938 fold)	regulate inflammation, immune responses, and angiogenesis	Diagnostic Biomarker
	brain tissue (Day 14 data)	animal	30	collected days 1/3/7/14/28 post-onset	decreased (-1.588347829 fold)	regulate inflammation, immune responses, and angiogenesis	Diagnostic Biomarker
	brain tissue (Day 28 data)	animal	30	collected days 1/3/7/14/28 post-onset	decreased (-1.715611956 fold)	regulate inflammation, immune responses, and angiogenesis	Diagnostic Biomarker
**3078-5p**	brain tissue (Day 7 data)	animal	30	collected days 1/3/7/14/28 post-onset	decreased (-1.64980255 fold)	regulate inflammation, immune responses, and angiogenesis	Diagnostic Biomarker
	brain tissue (Day 14 data)	animal	30	collected days 1/3/7/14/28 post-onset	decreased (-0.841949669 fold)	regulate inflammation, immune responses, and angiogenesis	Diagnostic Biomarker
**582-3p**	brain tissue (Day 7 data)	animal	30	collected days 1/3/7/14/28 post-onset	decreased (-1.777037688 fold)	regulate inflammation, immune responses, and angiogenesis	Diagnostic Biomarker
**467d-5p**	brain tissue (Day 7 data)	animal	30	collected days 1/3/7/14/28 post-onset	decreased (-1.895258076 fold)	regulate inflammation, immune responses, and angiogenesis	Diagnostic Biomarker
**6944-3p**	brain tissue (Day 7 data)	animal	30	collected days 1/3/7/14/28 post-onset	decreased (-2.151386768 fold)	regulate inflammation, immune responses, and angiogenesis	Diagnostic Biomarker
**146b-3p**	brain tissue (Day 7 data)	animal	30	collected days 1/3/7/14/28 post-onset	decreased (-2.29813493 fold)	regulate inflammation, immune responses, and angiogenesis	Diagnostic Biomarker
**6936-3p**	brain tissue (Day 7 data)	animal	30	collected days 1/3/7/14/28 post-onset	decreased (-3.060923409 fold)	regulate inflammation, immune responses, and angiogenesis	Diagnostic Biomarker
**181d-3p**	brain tissue (Day 7 data)	animal	30	collected days 1/3/7/14/28 post-onset	decreased (-3.349812973 fold)	regulate inflammation, immune responses, and angiogenesis	Diagnostic Biomarker
**6952-5p**	brain tissue (Day 7 data)	animal	30	collected days 1/3/7/14/28 post-onset	decreased (-3.598973137 fold)	regulate inflammation, immune responses, and angiogenesis	Diagnostic Biomarker
**7008-3p**	brain tissue (Day 7 data)	animal	30	collected days 1/3/7/14/28 post-onset	decreased (-3.599980881 fold)	regulate inflammation, immune responses, and angiogenesis	Diagnostic Biomarker
**365-1-5p**	brain tissue (Day 7 data)	animal	30	collected days 1/3/7/14/28 post-onset	decreased (-3.746991074 fold)	regulate inflammation, immune responses, and angiogenesis	Diagnostic Biomarker
**7224-3p**	brain tissue (Day 7 data)	animal	30	collected days 1/3/7/14/28 post-onset	decreased (-3.820542263 fold)	regulate inflammation, immune responses, and angiogenesis	Diagnostic Biomarker
	brain tissue (Day 28 data)	animal	30	collected days 1/3/7/14/28 post-onset	increased (1.759368212 fold)	regulate synapses, cognition, axonogenesis, and ion transmembrane transport	Diagnostic Biomarker

**7044-3p**		brain tissue (Day 7 data)	animal	30	collected days 1/3/7/14/28 post-onset	decreased (-3.98877876 fold)	regulate inflammation, immune responses, and angiogenesis	Diagnostic Biomarker
**1188-3p**		brain tissue (Day 7 data)	animal	30	collected days 1/3/7/14/28 post-onset	decreased (-4.408489953 fold)	regulate inflammation, immune responses, and angiogenesis	Diagnostic Biomarker
**344g-3p**		brain tissue (Day 7 data)	animal	30	collected days 1/3/7/14/28 post-onset	decreased (-4.543569984 fold)	regulate inflammation, immune responses, and angiogenesis	Diagnostic Biomarker
**3070-5p**		brain tissue (Day 7 data)	animal	30	collected days 1/3/7/14/28 post-onset	decreased (-5.349249381 fold)	regulate inflammation, immune responses, and angiogenesis	Diagnostic Biomarker
**3094-5p**		brain tissue (Day 7 data)	animal	30	collected days 1/3/7/14/28 post-onset	decreased (-5.378654359 fold)	regulate inflammation, immune responses, and angiogenesis	Diagnostic Biomarker
**7022-3p**		brain tissue (Day 7 data)	animal	30	collected days 1/3/7/14/28 post-onset	decreased (-5.547009493 fold)	regulate inflammation, immune responses, and angiogenesis	Diagnostic Biomarker
**21b**		brain tissue (Day 14 data)	animal	30	collected days 1/3/7/14/28 post-onset	increased (5.839898825 fold)	regulate synapses, cognition, axonogenesis, and ion transmembrane transport	Diagnostic Biomarker
**21c**		brain tissue (Day 14 data)	animal	30	collected days 1/3/7/14/28 post-onset	increased (5.839898825 fold)	regulate synapses, cognition, axonogenesis, and ion transmembrane transport	Diagnostic Biomarker
**466i-5p**		brain tissue (Day 14 data)	animal	30	collected days 1/3/7/14/28 post-onset	increased (5.579266535 fold)	regulate synapses, cognition, axonogenesis, and ion transmembrane transport	Diagnostic Biomarker
**7676-3p**		brain tissue (Day 14 data)	animal	30	collected days 1/3/7/14/28 post-onset	increased (5.44521705 fold)	regulate synapses, cognition, axonogenesis, and ion transmembrane transport	Diagnostic Biomarker
**7661-3p**		brain tissue (Day 14 data)	animal	30	collected days 1/3/7/14/28 post-onset	increased (5.288707498 fold)	regulate synapses, cognition, axonogenesis, and ion transmembrane transport	Diagnostic Biomarker
**7053-3p**		brain tissue (Day 14 data)	animal	30	collected days 1/3/7/14/28 post-onset	increased (4.946241321 fold)	regulate synapses, cognition, axonogenesis, and ion transmembrane transport	Diagnostic Biomarker
**3079-5p**		brain tissue (Day 14 data)	animal	30	collected days 1/3/7/14/28 post-onset	increased (4.930648122 fold)	regulate synapses, cognition, axonogenesis, and ion transmembrane transport	Diagnostic Biomarker
		brain tissue (Day 28 data)	animal	30	collected days 1/3/7/14/28 post-onset	increased (4.993598394 fold)	regulate synapses, cognition, axonogenesis, and ion transmembrane transport	Diagnostic Biomarker
**7061-5p**		brain tissue (Day 14 data)	animal	30	collected days 1/3/7/14/28 post-onset	increased (4.805584797 fold)	regulate synapses, cognition, axonogenesis, and ion transmembrane transport	Diagnostic Biomarker
**7677-3p**		brain tissue (Day 14 data)	animal	30	collected days 1/3/7/14/28 post-onset	increased (4.773389532 fold)	regulate synapses, cognition, axonogenesis, and ion transmembrane transport	Diagnostic Biomarker
**8094**		brain tissue (Day 14 data)	animal	30	collected days 1/3/7/14/28 post-onset	increased (4.759633561 fold)	regulate synapses, cognition, axonogenesis, and ion transmembrane transport	Diagnostic Biomarker
**6955-5p**		brain tissue (Day 14 data)	animal	30	collected days 1/3/7/14/28 post-onset	increased (4.659463975 fold)	regulate synapses, cognition, axonogenesis, and ion transmembrane transport	Diagnostic Biomarker

**7219-3p**		brain tissue (Day 14 data)	animal	30	collected days 1/3/7/14/28 post-onset	increased (4.275058451 fold)	regulate synapses, cognition, axonogenesis, and ion transmembrane transport	Diagnostic Biomarker
**10a-3p**		brain tissue (Day 14 data)	animal	30	collected days 1/3/7/14/28 post-onset	increased (4.238051366 fold)	regulate synapses, cognition, axonogenesis, and ion transmembrane transport	Diagnostic Biomarker
		brain tissue (Day 28 data)	animal	30	collected days 1/3/7/14/28 post-onset	increased (4.656688202 fold)	regulate synapses, cognition, axonogenesis, and ion transmembrane transport	Diagnostic Biomarker
**7035-3p**		brain tissue (Day 14 data)	animal	30	collected days 1/3/7/14/28 post-onset	increased (3.329347605 fold)	regulate synapses, cognition, axonogenesis, and ion transmembrane transport	Diagnostic Biomarker
**7075-3p**		brain tissue (Day 14 data)	animal	30	collected days 1/3/7/14/28 post-onset	increased (2.780179846 fold)	regulate synapses, cognition, axonogenesis, and ion transmembrane transport	Diagnostic Biomarker
**1933-3p**		brain tissue (Day 14 data)	animal	30	collected days 1/3/7/14/28 post-onset	increased (2.577308324 fold)	regulate synapses, cognition, axonogenesis, and ion transmembrane transport	Diagnostic Biomarker
**216a-3p**		brain tissue (Day 14 data)	animal	30	collected days 1/3/7/14/28 post-onset	increased (2.444178202 fold)	regulate synapses, cognition, axonogenesis, and ion transmembrane transport	Diagnostic Biomarker
**483-3p**		brain tissue (Day 14 data)	animal	30	collected days 1/3/7/14/28 post-onset	increased (2.046699312 fold)	regulate synapses, cognition, axonogenesis, and ion transmembrane transport	Diagnostic Biomarker
		brain tissue (Day 28 data)	animal	30	collected days 1/3/7/14/28 post-onset	increased (2.207079066 fold)	regulate synapses, cognition, axonogenesis, and ion transmembrane transport	Diagnostic Biomarker
**511-3p**		brain tissue (Day 14 data)	animal	30	collected days 1/3/7/14/28 post-onset	increased (1.862008027 fold)	regulate synapses, cognition, axonogenesis, and ion transmembrane transport	Diagnostic Biomarker
**7688-5p**		brain tissue (Day 14 data)	animal	30	collected days 1/3/7/14/28 post-onset	increased (1.280779237 fold)	regulate synapses, cognition, axonogenesis, and ion transmembrane transport	Diagnostic Biomarker
**339-5p**		brain tissue (Day 14 data)	animal	30	collected days 1/3/7/14/28 post-onset	increased (1.075025538 fold)	regulate synapses, cognition, axonogenesis, and ion transmembrane transport	Diagnostic Biomarker
		brain tissue (Day 28 data)	animal	30	collected days 1/3/7/14/28 post-onset	increased (0.92297809 fold)	regulate synapses, cognition, axonogenesis, and ion transmembrane transport	Diagnostic Biomarker
**8114**		brain tissue (Day 14 data)	animal	30	collected days 1/3/7/14/28 post-onset	increased (1.073705696 fold)	regulate synapses, cognition, axonogenesis, and ion transmembrane transport	Diagnostic Biomarker
		brain tissue (Day 28 data)	animal	30	collected days 1/3/7/14/28 post-onset	increased (1.069906222 fold)	regulate synapses, cognition, axonogenesis, and ion transmembrane transport	Diagnostic Biomarker
**378d**		brain tissue (Day 14 data)	animal	30	collected days 1/3/7/14/28 post-onset	increased (0.887699794 fold)	regulate synapses, cognition, axonogenesis, and ion transmembrane transport	Diagnostic Biomarker
**339-3p**		brain tissue (Day 14 data)	animal	30	collected days 1/3/7/14/28 post-onset	increased (0.858401418 fold)	regulate synapses, cognition, axonogenesis, and ion transmembrane transport	Diagnostic Biomarker
		brain tissue (Day 28 data)	animal	30	collected days 1/3/7/14/28 post-onset	increased (1.186105766 fold)	regulate synapses, cognition, axonogenesis, and ion transmembrane transport	Diagnostic Biomarker

**92a-3p**		brain tissue (Day 14 data)	animal	30	collected days 1/3/7/14/28 post-onset	increased (0.817122862 fold)	regulate synapses, cognition, axonogenesis, and ion transmembrane transport	Diagnostic Biomarker
**203-3p**		brain tissue (Day 14 data)	animal	30	collected days 1/3/7/14/28 post-onset	increased (0.681081804 fold)	regulate synapses, cognition, axonogenesis, and ion transmembrane transport	Diagnostic Biomarker
**219a-2-3p**		brain tissue (Day 14 data)	animal	30	collected days 1/3/7/14/28 post-onset	increased (0.666543355 fold)	regulate synapses, cognition, axonogenesis, and ion transmembrane transport	Diagnostic Biomarker
**423-3p**		brain tissue (Day 14 data)	animal	30	collected days 1/3/7/14/28 post-onset	increased (0.661310734 fold)	regulate synapses, cognition, axonogenesis, and ion transmembrane transport	Diagnostic Biomarker
		brain tissue (Day 28 data)	animal	30	collected days 1/3/7/14/28 post-onset	increased (1.122691733 fold)	regulate synapses, cognition, axonogenesis, and ion transmembrane transport	Diagnostic Biomarker
**93-5p**		brain tissue (Day 14 data)	animal	30	collected days 1/3/7/14/28 post-onset	increased (0.65972582 fold)	regulate synapses, cognition, axonogenesis, and ion transmembrane transport	Diagnostic Biomarker
**574-3p**		brain tissue (Day 14 data)	animal	30	collected days 1/3/7/14/28 post-onset	increased (0.655713201 fold)	regulate synapses, cognition, axonogenesis, and ion transmembrane transport	Diagnostic Biomarker
**15a-5p**		brain tissue (Day 14 data)	animal	30	collected days 1/3/7/14/28 post-onset	increased (0.595092684 fold)	regulate synapses, cognition, axonogenesis, and ion transmembrane transport	Diagnostic Biomarker
**125b-1-3p**		brain tissue (Day 14 data)	animal	30	collected days 1/3/7/14/28 post-onset	increased (0.593688222 fold)	regulate synapses, cognition, axonogenesis, and ion transmembrane transport	Diagnostic Biomarker
		brain tissue (Day 28 data)	animal	30	collected days 1/3/7/14/28 post-onset	increased (1.003822539 fold)	regulate synapses, cognition, axonogenesis, and ion transmembrane transport	Diagnostic Biomarker
**195a-5p**		brain tissue (Day 14 data)	animal	30	collected days 1/3/7/14/28 post-onset	increased (0.559296618 fold)	regulate synapses, cognition, axonogenesis, and ion transmembrane transport	Diagnostic Biomarker
**378c**		brain tissue (Day 14 data)	animal	30	collected days 1/3/7/14/28 post-onset	increased (0.553713638 fold)	regulate synapses, cognition, axonogenesis, and ion transmembrane transport	Diagnostic Biomarker
**298-5p**		brain tissue (Day 14 data)	animal	30	collected days 1/3/7/14/28 post-onset	increased (0.502233822 fold)	regulate synapses, cognition, axonogenesis, and ion transmembrane transport	Diagnostic Biomarker
**3086-5p**		brain tissue (Day 14 data)	animal	30	collected days 1/3/7/14/28 post-onset	decreased (-0.526380868 fold)	regulate inflammation, immune responses, and angiogenesis	Diagnostic Biomarker
**138-1-3p**		brain tissue (Day 14 data)	animal	30	collected days 1/3/7/14/28 post-onset	decreased (-0.53644236 fold)	regulate inflammation, immune responses, and angiogenesis	Diagnostic Biomarker
**598-3p**		brain tissue (Day 14 data)	animal	30	collected days 1/3/7/14/28 post-onset	decreased (-0.609168396 fold)	regulate inflammation, immune responses, and angiogenesis	Diagnostic Biomarker
		brain tissue (Day 28 data)	animal	30	collected days 1/3/7/14/28 post-onset	decreased (-1.395428745 fold)	regulate inflammation, immune responses, and angiogenesis	Diagnostic Biomarker
**128-2-5p**		brain tissue (Day 14 data)	animal	30	collected days 1/3/7/14/28 post-onset	decreased (-0.701383857 fold)	regulate inflammation, immune responses, and angiogenesis	Diagnostic Biomarker

**337-3p**	brain tissue (Day 14 data)	animal	30	collected days 1/3/7/14/28 post-onset	decreased (-0.76621179 fold)	regulate inflammation, immune responses, and angiogenesis	Diagnostic Biomarker
**let-7i-3p**	brain tissue (Day 14 data)	animal	30	collected days 1/3/7/14/28 post-onset	decreased (-0.809799794 fold)	regulate inflammation, immune responses, and angiogenesis	Diagnostic Biomarker
	brain tissue (Day 28 data)	animal	30	collected days 1/3/7/14/28 post-onset	decreased (-1.396561717 fold)	regulate inflammation, immune responses, and angiogenesis	Diagnostic Biomarker
**449a-5p**	brain tissue (Day 14 data)	animal	30	collected days 1/3/7/14/28 post-onset	decreased (-1.103742153 fold)	regulate inflammation, immune responses, and angiogenesis	Diagnostic Biomarker
**505-5p**	brain tissue (Day 14 data)	animal	30	collected days 1/3/7/14/28 post-onset	decreased (-1.120403922 fold)	regulate inflammation, immune responses, and angiogenesis	Diagnostic Biomarker
**344c-3p**	brain tissue (Day 14 data)	animal	30	collected days 1/3/7/14/28 post-onset	decreased (-1.938040399 fold)	regulate inflammation, immune responses, and angiogenesis	Diagnostic Biomarker
**1927**	brain tissue (Day 14 data)	animal	30	collected days 1/3/7/14/28 post-onset	decreased (-2.777286754 fold)	regulate inflammation, immune responses, and angiogenesis	Diagnostic Biomarker
**6769b-3p**	brain tissue (Day 14 data)	animal	30	collected days 1/3/7/14/28 post-onset	decreased (-3.152517822 fold)	regulate inflammation, immune responses, and angiogenesis	Diagnostic Biomarker
**7668-3p**	brain tissue (Day 28 data)	animal	30	collected days 1/3/7/14/28 post-onset	increased (5.972697465 fold)	regulate synapses, cognition, axonogenesis, and ion transmembrane transport	Diagnostic Biomarker
**5107-5p**	brain tissue (Day 28 data)	animal	30	collected days 1/3/7/14/28 post-onset	increased (5.526831929 fold)	regulate synapses, cognition, axonogenesis, and ion transmembrane transport	Diagnostic Biomarker
**511-5p**	brain tissue (Day 28 data)	animal	30	collected days 1/3/7/14/28 post-onset	increased (5.241051656 fold)	regulate synapses, cognition, axonogenesis, and ion transmembrane transport	Diagnostic Biomarker
**5114**	brain tissue (Day 28 data)	animal	30	collected days 1/3/7/14/28 post-onset	increased (4.987536654 fold)	regulate synapses, cognition, axonogenesis, and ion transmembrane transport	Diagnostic Biomarker
**1956**	brain tissue (Day 28 data)	animal	30	collected days 1/3/7/14/28 post-onset	increased (4.593842089 fold)	regulate synapses, cognition, axonogenesis, and ion transmembrane transport	Diagnostic Biomarker
**6997-5p**	brain tissue (Day 28 data)	animal	30	collected days 1/3/7/14/28 post-onset	increased (3.378765837 fold)	regulate synapses, cognition, axonogenesis, and ion transmembrane transport	Diagnostic Biomarker
**1247-5p**	brain tissue (Day 28 data)	animal	30	collected days 1/3/7/14/28 post-onset	increased (2.000510101 fold)	regulate synapses, cognition, axonogenesis, and ion transmembrane transport	Diagnostic Biomarker
**3074-5p**	brain tissue (Day 28 data)	animal	30	collected days 1/3/7/14/28 post-onset	increased (1.87633388 fold)	regulate synapses, cognition, axonogenesis, and ion transmembrane transport	Diagnostic Biomarker
**542-5p**	brain tissue (Day 28 data)	animal	30	collected days 1/3/7/14/28 post-onset	increased (1.795195316 fold)	regulate synapses, cognition, axonogenesis, and ion transmembrane transport	Diagnostic Biomarker
**205-5p**	brain tissue (Day 28 data)	animal	30	collected days 1/3/7/14/28 post-onset	increased (1.31563489 fold)	regulate synapses, cognition, axonogenesis, and ion transmembrane transport	Diagnostic Biomarker

**455-3p**		brain tissue (Day 28 data)	animal	30	collected days 1/3/7/14/28 post-onset	increased (1.307081559 fold)	regulate synapses, cognition, axonogenesis, and ion transmembrane transport	Diagnostic Biomarker
**30c-1-3p**		brain tissue (Day 28 data)	animal	30	collected days 1/3/7/14/28 post-onset	increased (0.737444462 fold)	regulate synapses, cognition, axonogenesis, and ion transmembrane transport	Diagnostic Biomarker
**1839-3p**		brain tissue (Day 28 data)	animal	30	collected days 1/3/7/14/28 post-onset	decreased (-0.661098951 fold)	regulate inflammation, immune responses, and angiogenesis	Diagnostic Biomarker
**218-5p**		brain tissue (Day 28 data)	animal	30	collected days 1/3/7/14/28 post-onset	decreased (-0.761054659 fold)	regulate inflammation, immune responses, and angiogenesis	Diagnostic Biomarker
**493-5p**		brain tissue (Day 28 data)	animal	30	collected days 1/3/7/14/28 post-onset	decreased (-0.766472138 fold)	regulate inflammation, immune responses, and angiogenesis	Diagnostic Biomarker
**1839-5p**		brain tissue (Day 28 data)	animal	30	collected days 1/3/7/14/28 post-onset	decreased (-0.767956989 fold)	regulate inflammation, immune responses, and angiogenesis	Diagnostic Biomarker
**212-3p**		brain tissue (Day 28 data)	animal	30	collected days 1/3/7/14/28 post-onset	decreased (-0.772327627 fold)	regulate inflammation, immune responses, and angiogenesis	Diagnostic Biomarker
**370-5p**		brain tissue (Day 28 data)	animal	30	collected days 1/3/7/14/28 post-onset	decreased (-0.91410213 fold)	regulate inflammation, immune responses, and angiogenesis	Diagnostic Biomarker
**344-3p**		brain tissue (Day 28 data)	animal	30	collected days 1/3/7/14/28 post-onset	decreased (-0.924381141 fold)	regulate inflammation, immune responses, and angiogenesis	Diagnostic Biomarker
**344b-3p**		brain tissue (Day 28 data)	animal	30	collected days 1/3/7/14/28 post-onset	decreased (-0.931041256 fold)	regulate inflammation, immune responses, and angiogenesis	Diagnostic Biomarker
**124-5p**		brain tissue (Day 28 data)	animal	30	collected days 1/3/7/14/28 post-onset	decreased (-0.938184195 fold)	regulate inflammation, immune responses, and angiogenesis	Diagnostic Biomarker
**362-5p**		brain tissue (Day 28 data)	animal	30	collected days 1/3/7/14/28 post-onset	decreased (-1.030547377 fold)	regulate inflammation, immune responses, and angiogenesis	Diagnostic Biomarker
**592-5p**		brain tissue (Day 28 data)	animal	30	collected days 1/3/7/14/28 post-onset	decreased (-1.070589811 fold)	regulate inflammation, immune responses, and angiogenesis	Diagnostic Biomarker
**33-3p**		brain tissue (Day 28 data)	animal	30	collected days 1/3/7/14/28 post-onset	decreased (-1.131330939 fold)	regulate inflammation, immune responses, and angiogenesis	Diagnostic Biomarker
**299a-3p**		brain tissue (Day 28 data)	animal	30	collected days 1/3/7/14/28 post-onset	decreased (-1.179481463 fold)	regulate inflammation, immune responses, and angiogenesis	Diagnostic Biomarker
**299b-3p**		brain tissue (Day 28 data)	animal	30	collected days 1/3/7/14/28 post-onset	decreased (-1.181687654 fold)	regulate inflammation, immune responses, and angiogenesis	Diagnostic Biomarker
**383-3p**		brain tissue (Day 28 data)	animal	30	collected days 1/3/7/14/28 post-onset	decreased (-1.885964907 fold)	regulate inflammation, immune responses, and angiogenesis	Diagnostic Biomarker
